# The effects of acarbose treatment on cardiovascular risk factors in impaired glucose tolerance and diabetic patients: a systematic review and dose–response meta-analysis of randomized clinical trials

**DOI:** 10.3389/fnut.2023.1084084

**Published:** 2023-08-01

**Authors:** Mohammad Zamani, Mahlagha Nikbaf-Shandiz, Yasaman Aali, Niloufar Rasaei, Mahtab Zarei, Farideh Shiraseb, Omid Asbaghi

**Affiliations:** ^1^Department of Clinical Nutrition, School of Nutritional Sciences and Dietetics, Tehran University of Medical Sciences, Tehran, Iran; ^2^Student Research Committee, Tabriz University of Medical Sciences, Tabriz, Iran; ^3^Department of Community Nutrition, School of Nutritional Sciences and Dietetics, Tehran University of Medical Sciences (TUMS), Tehran, Iran; ^4^Department of Cellular and Molecular Nutrition, School of Nutritional Sciences and Dietetics, Tehran University of Medical Sciences (TUMS), Tehran, Iran; ^5^Cancer Research Center, Shahid Beheshti University of Medical Sciences, Tehran, Iran; ^6^Student Research Committee, Shahid Beheshti University of Medical Sciences, Tehran, Iran

**Keywords:** acarbose, cardiovascular risk factors, systematic review, meta-analysis, diabetic patients

## Abstract

Acarbose (ACB) seems to be an effective drug in the management of cardiovascular risk factors. However, no previous meta-analysis of randomized controlled trials (RCTs) has been done to evaluate the effects of ACB on cardiovascular risk factors on impaired glucose tolerance (IGT), type 2 diabetes mellitus (T2D), and type 1 diabetes mellitus (T1D). We comprehensively searched electronic databases including Scopus, Web of Science, and PubMed for RCTs for related keywords up to September 2022. A random-effects model was used to estimate the weighted mean difference (WMD) and 95% confidence interval (CI). The pooled analysis demonstrated that ACB treatment had a significant effect on fasting blood glucose (FBG) (WMD = −3.55 mg/dL; 95%CI: −6.29, −0.81; *p* = 0.011), fasting insulin (WMD = −6.73 pmoL/L; 95%CI: −10.37, −3.10; *p* < 0.001), HbA1c [WMD = −0.32%; 95%CI: −0.45, −0.20; *p* < 0.001], body weight (WMD = −1.25 kg; 95%CI: −1.79, −0.75; *p* < 0.001), body mass index (BMI) (WMD = −0.64 kg/m^2^; 95%CI: −0.92, −0.37; *p* < 0.001), tumor necrosis factor-alpha (TNF-α) (WMD = −2.70 pg/mL, 95%CI: −5.25, −0.16; *p* = 0.037), leptin (WMD = −1.58 ng/mL; 95%CI: −2.82, −0.35; *p* = 0.012), alanine transaminase (ALT) (WMD = 0.71 U/L; 95%CI: −0.31, 1.85; *p* = 0.164), triglyceride (TG) (WMD = −13.89 mg/dL; 95%CI: −20.69, −7.09; *p* < 0.001), total cholesterol (TC) (WMD = −2.26 mg/dL; 95%CI: −4.18, −0.34; *p* = 0.021), systolic blood pressure (SBP) (WMD = −1.29 mmHg; 95%CI: −2.44, −0.15; *p* = 0.027), and diastolic blood pressure (DBP) (WMD = 0.02 mmHg; 95%CI: −0.41, 0.45; *p* = 0.925) in an intervention group, compared with a placebo group. The non-linear dose–response analysis showed that ACB reduces the TC in trial duration by >50 weeks, and 180 mg/day is more effective for the decrement of CRP. ACB can improve lipid profiles, glycemic indices, anthropometric indices, and inflammatory markers in T2D, T1D, and IGT patients.

## Introduction

Cardiovascular diseases (CVDs) are the leading cause of global mortality (1) that impose a considerable economic burden on both governments and individuals (2). CVDs are primarily associated with several key risk factors, including elevated systolic blood pressure (SBP), increased fasting plasma glucose (FPG) levels, elevated low-density lipoprotein (LDL) cholesterol, and a high body mass index (BMI) (1). Compared with adults without diabetes, individuals with diabetes experience a 2- to 4-fold increase in cardiovascular rise (3). The increased risk of mortality in diabetes patients is mainly due to CVDs (3). Diabetes has become a pressing global issue, particularly with the rise of type 2 diabetes (T2D), which contributes significantly to mortality and disability rates (4), and is more prevalent (5) compared with type 1. In addition to T2D, another concern is impaired glucose tolerance (IGT) (5, 6). Diabetes is also linked to dyslipidemia (7), elevated liver enzymes (8), elevated inflammatory factors (9), polycystic ovarian syndrome (10, 11), and overweight or obesity (12, 13). Some factors can modify the relationship between diabetes and CVDs such as lifestyle (14), physical activity (15), dietary intake (16–18), and pharmacotherapy (19).

Acarbose (ACB), a pseudo-tetrasaccharide, is classified as an α-glucosidase inhibitor (20) that has shown comparable efficacy to metformin in the management of diabetes (21). The strong binding affinity of ACB to α-glucosidase enzymes inhibits the absorption of polysaccharides from the intestine (20). The findings of a significant multicenter placebo-controlled trial conducted by Chiasson et al. demonstrated that the intake of acarbose (ACB) can effectively reduce the occurrence of major cardiovascular events among patients with impaired glucose tolerance (IGT) (22). A meta-analysis of 8 RCTs by Mannucci et al. reported that the evidence is insufficient to conclude any beneficial effect of α-glucosidase-inhibiting (AGI) drugs on major cardiovascular events in T2D patients (23). Another meta-analysis of 66 RCTs in 2021 by Alssema et al. supported the acute reduction in postprandial glucose and postprandial insulin following AGI drug intake in diabetic and non-diabetic individuals. A meta-analysis of seven studies conducted by Yu et al. provided evidence supporting the beneficial effect of acarbose (ACB) therapy in reducing triglyceride (TG) levels among non-diabetic patients who are overweight or obese. This suggests the potential usefulness of ACB in managing TG levels in this population (24). Another study by Schnell et al. pooled the data from 10 previous studies and concluded that ACB treatment can reduce body weight independent of glycemic control in patients with diabetes (25). Hu et al. assessed the preventive effect of ACB monotherapy on T2D incidence by a meta-analysis of 8 RCTs in 2015. Interestingly, this preventive effect seems to be superior in Eastern populations with prediabetes compared with Western populations (26). However, few studies focused on the effect of ACB in T1D patients. However, a pooled analysis of seven trials conducted by Liu et al. revealed promising results. The addition of ACB to insulin therapy demonstrated improvements in overall glucose control among T1D patients, including reductions in HbA1c levels, mean blood glucose, fasting blood glucose (FBG), postprandial glucose (PPG), and glucose variability. These findings suggest that ACB may have a positive effect on glycemic management in T1D patients when used in combination with insulin therapy (27).

Considering the heterogeneity and inconsistent results of previous reports, as well as the absence of a comprehensive meta-analysis examining the cardiovascular risk factors associated with ACB treatment, the objective of this study is to conduct a conclusive dose–response meta-analysis. The aim is to comprehensively assess the impact of ACB treatment on various cardiovascular risk factors in patients with diabetes and impaired glucose tolerance (IGT). By employing a comprehensive cardiovascular risk assessment approach, this study seeks to provide a more robust and conclusive analysis of the effects of ACB treatment in this patient population.

## Methods

Preferred reporting items for systematic reviews and meta-analyses (PRISMA) were used in this study (28). This study is registered at PROSPERO (CRD42022355832).

### Search strategy

We have performed a systematic literature search of articles in scientific databases, namely PubMed, Scopus, and Web of Science published up to September 2022 to find any relevant RCTs about the effect of ACB treatment on CVD risk factors. To search for items related to ACB and CVD risk factors, we used PICO (Participant: T2D, T1D, and IGT patients; Intervention: ACB; Comparison/Control: control group; Outcome: CVD risk factor). The keywords used for searching are as follows: (Acarbose) AND (Intervention OR “intervention study” OR “intervention studies” OR “controlled trial” OR randomized OR random OR randomly OR placebo OR “clinical trial” OR “RCT” OR blinded OR “double blind” OR “double blinded” OR trial OR “clinical trial” OR trials OR “pragmatic clinical trial” OR “cross-over studies” OR “cross-over” OR “cross-over study” OR “parallel study” OR “parallel trial”). Google Scholar and reference lists of the included studies and previous review studies were checked to avoid missing relevant articles (Supplementary material 1).

### Study selection

We included studies with the following criteria: (1) randomized controlled clinical trials (parallel or crossover); (2) human studies; (3) adults (≥18 years) with T1D, T2D, or IGT; (4) mean ± standard deviation or effect size reported for outcomes; and (5) examined the effect of ACB intake on CVD risk factors including serum TG, total cholesterol (TC), low-density lipoprotein (LDL), high-density lipoprotein (HDL), FBG, hemoglobin A1c (HbA1c), serum insulin, HOMA-IR, systolic blood pressure (SBP), diastolic blood pressure (DBP), c reactive protein (CRP), interleukin 6 (IL-6), *tumor necrosis factor* (TNF-α), adiponectin, leptin, weight, waist circumference (WC), body mass index (BMI), aspartate transaminase (AST), alanine transaminase (ALT), and *alkaline phosphatase* (ALP). We excluded animal and *in vitro* studies, studies on children and adolescents, gray literature, reviews, conference abstracts, editorials, books, and RCTs that did not have control/placebo groups. We imposed no restrictions on the time, date, length, and language of studies and the dosage of ACB treatment. Two authors (OA and MZ) independently screened the title and abstracts of the included studies for the first screening and the full texts for the second-level screening. They extracted results and assessed the studies’ qualifications. Any uncertainty regarding the inclusion of studies was resolved through discussion.

### Data extraction

The full texts of all the included studies were studied separately, and the following information was extracted by two investigators (OA and MZ): name of the first author, year of publication, country, type of clinical trial, participant characteristics (mean age, BMI, and sex), duration of intervention, randomization, blinding, sample size, the number of participants in the intervention and control groups, form and dosage of ACB, the health status of participants, and outcome values. All ACB intake doses were converted to mg/day.

### Quality assessment

To assess the quality of the included studies, we used the Cochrane Collaboration tool (29). The included studies were screened for any source of bias including random sequence generation, allocation concealment, participant and staff blindness, outcome assessor blinding, incomplete outcome data, selective reporting, and other biases. Finally, three groups of high, moderate, and low risk of bias were defined. Two authors (OA and MZ) separately assessed the quality of the research articles, and any conflicting opinions were settled through discussion.

### Statistical analysis

Statistical analyses were conducted using Stata version 11.0 (Stata Corp, College Station, TX). All tests were two-tailed with *p*-values <0.05 considered statistically significant. Pooled weighted mean difference (WMD) was calculated to assess the existing heterogeneity using a random-effects model (30). We calculated mean differences in our outcomes from baseline to the post-intervention between the ACB-treated and control groups. The standard deviation (SD) of the mean difference was calculated using the following formula: SD = square root [(SD at baseline)^2^+ (SD at the end of study)^2^ − (2 *r* × SD at baseline ×SD at the end of study)] (31). In studies reporting standard errors (SEs), 95% confidence intervals (CIs), or interquartile ranges (IQRs), we used the following Hozo et al.’s formula to transform these values into SDs: SD = SE × √*n* (*n* = the number of individuals in each group) (32). A correlation coefficient of 0.8 was used for r (33). A subgroup analysis was performed to determine the source of heterogeneity. Subgroups were selected based on the required minimum number of studies according to the criteria provided by Fu et al. (34). There should be at least 6 to 10 studies for continuous subgroup variables and a minimum of 4 studies for categorical subgroup variables (34, 35). Subgroup analyses were performed separately for normal or abnormal levels of each analyzed parameter, glycemic status (T1D, T2D, IGT), different doses (more or less than 300 mg/day), different durations (more or less than 24 weeks), and ethnicity (Eastern/Western). The *I*^2^ or Cochrane’s Q test was used to measure statistical heterogeneity (36), with values greater than 40% indicating strong heterogeneity (37). To detect any publication bias, the funnel plot, Begg’s rank correlation, and Egger’s regression tests were used (38, 39). The leave-one-out method (i.e., deleting one trail at a time and recalculating the impact size) was used to examine the impact of each study on the pooled effect size. Sensitivity analysis was carried out to determine how many inferences were dependent on a particular sample. To identify and mitigate the effects of the publishing bias, we employed the trim-and-fill method (40). The possible impact of ACB (mg/d) dosage and duration on liver enzymes was evaluated using meta-regression. Additionally, we employed a non-linear dose–response analysis to synthesize the associated dose–response data from several research for the dose–response analysis between ACB intake and CVD risk factors (41, 42).

### Certainty assessment

As previously mentioned, the certainty of evidence in the included research was examined and summarized using the GRADE (Grading of Recommendations Assessment, Development, and Evaluation) technique (43).

## Results

### The flow of study selection

**Figure 1:**
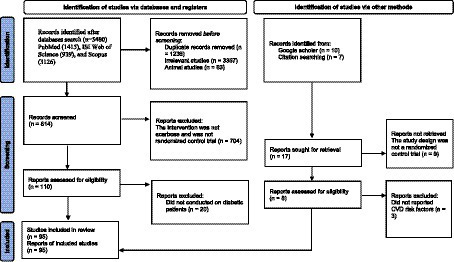
Flowchart of study selection for inclusion trials in the systematic review.

We presented the flowchart in Figure 1 and described the selection process and the references retrieved from the database in this figure. We identified a total of 5,480 studies in the first step of the electronic databases search. We excluded duplicated (*n* = 1,236) and irrelevant studies (*n* = 3,367) and animal studies (*n* = 63). Then, 814 studies were evaluated based on titles and abstracts. Among these, 704 studies were excluded because the intervention was not acarbose and it was not a randomized control trial. Then, 110 full-text relevant articles were reviewed. Among these, 20 studies were excluded because they were conducted on non-diabetic subjects. Eventually, 90 articles were identified. On the other hand, five studies were identified through a manual search and a review of reference lists. Finally, 95 studies were included in the qualitative synthesis. Therefore, we included a total of 95 studies (21, 44–137) in the present systematic review and meta-analysis, and their characteristics are presented in Table 1.

**Table 1:** Characteristic of included studies in the meta-analysis.

Studies	Country	Study design	Participant	Sample size and sex	Sample size		Trial duration (week)	Means age		Means BMI		Intervention		Adverse events
					IG	CG		IG	CG	IG	CG	Acarbose (mg/d)	Control group	
Akazawa et al. (1982)	China	Parallel, R, PC	Type 2 patients	M/F; 24	10	14	7	20–79	20–79	NR	NR	300	Glucomannan	NR
Scott et al. (1984)	New Zealand	Crossover, R, PC	Type 2 patients	M/F: 18	18	18	4	55.5 ± 7.1	55.5 ± 7.1	NR	NR	300	Placebo	NR
Hanefeld et al. (1991)	Germany	Parallel, R, PC, DB	Type 2 patients	M/F: 94	47	47	24	60 ± 16.5	59 ± 16.5	27.4 ± 7.85	27.7 ± 8.5	300	Placebo	Flatulence, abdominal distension, and diarrhea
Hotta et al. (1993)	Japan	Parallel, R, PC, DB	Type 2 patients	M/F: 37	19	18	24	49.8 ± 17.5	47.9 ± 18	23.5 ± 4.15	22.9 ± 4.4	300	Placebo	NR
Jenney et al. (1993)	Australia	Crossover, R, PC, DB	Type 2 patients	M/F: 6	6	6	12	60.3 ± 2.5	60.3 ± 2.5	NR	NR	75	Placebo	NR
Coniff et al. (1994)	USA	Parallel, R, PC, DB	Type 2 patients	M/F: 189	91	98	12	56 ± 9.5	55.8 ± 10	32 ± 16.75	31.5 ± 12.3	300	Placebo	Abdominal pain, nausea, diarrhea, and flatulence
Hoffman et al. (1994)	Germany	Parallel, R, PC, DB	Type 2 patients	M/F: 58	28	30	24	58.8 ± 6.9	56.9 ± 6.7	26.5 ± 1.6	26.8 ± 1.5	300	Placebo	NR
Coniff et al. (1995)	USA	Parallel, R, PC, DB	Type 2 patients	M/F: 207	103	104	24	NR	NR	NR	NR	300	Placebo	Diarrhea and flatulence
Wolever et al. (1995)	Canada	Parallel, R, PC, DB	Type 2 patients	M/F: 85	41	44	52	54.4 ± 11.5	57.6 ± 9.7	31.9 ± 6.3	29.7 ± 4.5	400	Placebo	No side effect
Chiasson et al. (1996)	Canada	Parallel, R, PC, DB	Impaired glucose tolerance	M/F: 18	8	10	16	56.1 ± 8.7	55.4 ± 8.7	32.2 ± 6.9	29.3 ± 2.7	150	Placebo	No side effect
Bayraktar et al. (1996)	Turkey	Crossover, R, PC	Type 2 patients	M/F: 18	18	18	8	49	49	NR	NR	300	Metformin	NR
Noda et al. (1997)	Japan	Parallel, R, PC	Type 2 patients	M/F: 20	14	6	24	56 ± 8	53 ± 9	22.9 ± 0.8	27 ± 2.5	300	Control group	NR
Hoffmann et al. (1997)	Germany	Parallel, R, PC, DB	Type 2 patients	M/F: 63	31	32	24	58.9 ± 9.4	60.2 ± 8.6	26.4 ± 2.7	26.3 ± 2.2	300	Placebo	NR
Costa et al. (1997)	Spain	Parallel, R, PC, DB	Type 2 patients	M/F: 65	36	29	24	60.2 ± 8.4	61.7 ± 9	28.7 ± 4.2	27.4 ± 3	300	Placebo	Constipation, nausea, diarrhea, and flatulence
Chan et al. (1998)	China	Parallel, R, PC, DB	Type 2 patients	M/F: 126	63	63	24	52.8 ± 10.2	54 ± 10	25.4 ± 3.9	25.6 ± 3.8	300	Placebo	No side effect
Guagnano et al. (1998)	Italy	Parallel, R, PC	Type 2 patients	M/F: 34	17	17	12	62.58 ± 9.63	62.41 ± 9.79	30.21 ± 5.62	30.15 ± 5.41	300	Control group	Flatulence, abdominal cramps, and diarrhea
Bayraktar et al. (1998)	Turkey	Parallel, R, PC	Type 2 patients	F: 50	25	25	12	38.12 ± 11.25	37.08 ± 9.5	34.83 ± 5.05	37.26 ± 5.9	300	Control group	NR
Soonthornpun et al. (1998)	Thailand	Crossover, R, PC, DB	Type 2 patients	M/F: 15	15	15	12	57.5 ± 2.6	57.5 ± 2.6	NR	NR	300	Placebo	Mild and tolerable gastrointestinal problems
Lam et al. (1998)	China	Parallel, R, PC, DB	Type 2 patients	M/F: 89	45	44	24	57.8 ± 9.1	56.9 ± 7.12	24.8 ± 3.5	24.1 ± 2.8	300	Placebo	NR
Buchanan et al. (1998)	UK	Parallel, R, PC, DB	Type 2 patients	M/F: 20	9	11	16	60.1 ± 6.8	57.6 ± 8.2	NR	NR	350	Placebo	Diarrhea and flatulence
Sels et al. (1998)	Netherlands	Crossover, R, PC	Type 1 patients	M/F: 62	62	62	8	38.3 ± 23	35.3 ± 23	NR	NR	300	Placebo	Flatulence, diarrhea, and abdominal pain
Fischer et al. (1998) (A)	Germany	Parallel, R, PC, DB	Type 2 patients	M/F: 167	86	81	24	58.5 ± 8.4	52.7 ± 8.7	27.3 ± 3.5	26.9 ± 2.9	75	Placebo	Flatulence and meteorism
Fischer et al. (1998) (B)	Germany	Parallel, R, PC, DB	Type 2 patients	M/F: 169	88	81	24	55.5 ± 9.6	52.7 ± 9.7	27.6 ± 3.5	26.9 ± 2.9	150	Placebo	Flatulence and meteorism
Fischer et al. (1998) (C)	Germany	Parallel, R, PC, DB	Type 2 patients	M/F: 159	78	81	24	56.8 ± 9.4	52.7 ± 9.7	27.6 ± 3.7	25.9 ± 2.9	300	Placebo	Flatulence and meteorism
Fischer et al. (1998) (D)	Germany	Parallel, R, PC, DB	Type 2 patients	M/F: 168	87	81	24	59.4 ± 8.6	52.7 ± 8.7	27.2 ± 3.3	26.9 ± 2.9	600	Placebo	Flatulence and meteorism
Standl et al. (1999)	Germany	Parallel, R, PC, DB	Type 2 patients	M/F: 481	24	24	24	59.3 ± 8.5	62.9 ± 9.4	25.2 ± 2.2	24.1 ± 2	600	Placebo	NR
López‐Alvarenga et al. (1999)	Mexico	Crossover, R, PC, DB	Type 2 patients	M/F: 17	17	17	12	56.7 ± 7.7	51.75 ± 7.2	27.5 ± 2.6	25.7 ± 1.8	300	Placebo	Gastrointestinal problems
Holman et al. (1999)	United Kingdom	Parallel, R, PC, DB	Type 2 patients	M/F: 1,946	973	973	156	60 ± 9	60 ± 9	29.8 ± 5.6	29.6 ± 5.7	300	Placebo	No side effect
Salman et al. (2000)	Turkey	Parallel, R, PC	Type 2 patients	M/F: 57	27	30	24	52.6 ± 9.1	56.1 ± 8.7	30.2 ± 3.8	29.2 ± 2.8	300	Gliclazide	Flatulence, abdominal pain, and diarrhea
Meneilly et al. (2000)	Canada	Parallel, R, PC, DB	Type 2 patients	M/F: 45	22	23	52	68 ± 4.5	70 ± 4.6	28 ± 4.5	29 ± 4.6	300	Placebo	No side effect
Halimi et al. (2000)	France	Parallel, R, PC, DB	Type 2 patients	M/F: 129	59	70	24	56 ± 9.2	55 ± 10	30.1 ± 3.3	29.7 ± 3.3	300	Placebo	No side effect
Ko et al. (2001)	China	Parallel, R, PC	Type 2 patients	M/F: 57	27	30	52	58.5 ± 9.9	59.1 ± 12.5	24.3 ± 3.8	24.9 ± 3.4	300	Insulin	Flatulence, diarrhea, and abdominal colic
Gentile et al. (2001)	Italy	Parallel, R, PC, DB	Type 2 patients	M/F; 100	52	48	28	NR	NR	27.8 ± 15	27.8 ± 15	300	Placebo	NR
Takei et al. (2001)	Japan	Parallel, R, PC	Type 2 patients	M/F: 15	6	9	12	56.7 ± 10.6	57.7 ± 10	28.2 ± 3.8	27.1 ± 2.4	150	Control group	NR
Hanefeld et al. (2002)	Germany	Parallel, R, PC, DB	Type 2 patients	M/F: 19	11	8	16	60.4 ± 3.9	59 ± 4.8	27.5 ± 2.4	27.2 ± 3.3	300	Placebo	NR
Vichayanrat et al. (2002)	Thailand	Crossover, R, PC	Type 2 patients	M/F: 30	30	30	8	55 ± 11.6	55 ± 11.6	21.1 ± 3.6	21.1 ± 3.6	300	Voglibose	NR
Rosenthal et al. (2002)	Germany	Parallel, R, PC	Type 2 patients	M/F: 76	39	37	24	57.4 ± 8.6	57.7 ± 10.5	29.1 ± 4.3	28.8 ± 4.3	300	Glibenclamide	No side effect
Göke et al. (2002)	Germany	Parallel, R, PC	Type 2 patients	M/F: 265	136	129	26	58.8 ± 9.1	58.9 ± 9.1	30.8 ± 4.4	30.9 ± 5.3	300	Pioglitazone	No side effect
Rosenbaum et al. (2002)	Brazil	Parallel, R, PC, DB	Type 2 patients	M/F: 40	20	20	22	59.8 ± 8.2	62 ± 9.7	30.3 ± 2.9	31.7 ± 3.9	300	Placebo	Increased liver enzymes, cardiac failure, and gastrointestinal problems
Fischer et al. (2003)	Germany	Parallel, R, PC, DB	Type 2 patients	M/F: 50	25	25	16	59.4 ± 28	58.6 ± 31.5	27.3 ± 4	27 ± 3.5	300	Placebo	No side effect
Pan et al. (2003)	China	Parallel, R, PC, DB	Impaired glucose tolerance	M/F: 252	125	127	16	53.4 ± 8.63	55.6 ± 8.31	25.6 ± 2.99	25.8 ± 3.22	150	Placebo	Gastrointestinal problems
Josse et al. (2003)	Canada	Parallel, R, PC, DB	Type 2 patients	M/F: 192	93	99	52	69.7 ± 5	70.3 ± 5	28.6 ± 4	28.3 ± 4	150	Placebo	No side effect
Lin et al. (2003)	Taiwan	Parallel, R, PC, DB	Type 2 patients	M/F: 64	32	32	24	57.7 ± 7.3	55.4 ± 8.5	24.8 ± 3	25.1 ± 2.8	300	Placebo	Gastrointestinal problems
Bachmann et al. (2003)	Germany	Parallel, R, PC, DB	Type 2 patients	M/F: 330	164	166	78	63.8 ± 7.1	63.3 ± 7.2	29 ± 3.1	29 ± 2.9	300	Placebo	NR
Hwu et al. (2003)	Taiwan	Parallel, R, PC, DB	Type 2 patients	M/F: 107	54	53	18	58.1 ± 8.4	54.7 ± 8.6	24.2 ± 3.5	23.9 ± 3.7	300	Placebo	NR
Yajima et al. (2004)	Japan	Parallel, R, PC	Type 2 patients	M/F: 22	11	11	12	58.7 ± 7.5	56.10 ± 7.6	25 ± 2.65	26.1 ± 2.9	300	Metformin	NR
Watanabe et al. (2004)	Japan	Parallel, R, PC	Type 2 patients	M/F: 20	10	10	4	56.2 ± 5.9	54.2 ± 4.2	26.7 ± 2.5	23.3 ± 3	300	Voglibose	NR
van de Laar et al. (2004)	Netherlands	Parallel, R, PC, DB	Type 2 patients	M/F: 96	48	48	8	59.3 ± 7.5	57.8 ± 7.3	29.1 ± 4.6	29 ± 4.8	300	Tolbutamide	Flatulence, diarrhea, abdominal pain or nausea, headache
Göke et al. (2004)	Germany	Parallel, R, PC	Type 2 patients	M/F: 140	71	69	26	58.9 ± 9.1	58.9 ± 9.1	30.9 ± 4.9	30.9 ± 4.9	300	Pioglitazone	NR
Gentile et al. (2005)	Italy	Crossover, R, PC, DB	Type 2 patients	M/F: 107	107	107	8	59.3 ± 6.4	59.3 ± 6.4	27.4 ± 1.6	27.4 ± 1.6	300	Control group	No side effect
Inoue et al. (2006)	Japan	Parallel, R, PC	Impaired glucose tolerance	M/F: 40	20	20	12	NR	NR	27.5 ± 3.8	27.5 ± 4	300	Placebo	NR
Suzuki et al. (2006)	Japan	Parallel, R, PC	Type 2 patients	M/F: 330	16	17	24	67.9 ± 9.9	68.8 ± 12	25 ± 2.8	25.6 ± 4	150	Colestimide	NR
Wagner et al. (2006)	Sweden	Parallel, R, PC	Type 2 patients	M/F: 31	14	17	12	57 ± 3.5	54 ± 4	28.7 ± 3.3	28.7 ± 4.7	300	Control group	NR
Schnell et al. (2007)	Germany	Parallel, R, PC, DB	Type 2 patients	M/F: 163	82	81	20	61.5 ± 8.9	62.3 ± 7.4	30.4 ± 4.2	29.9 ± 4.5	300	Placebo	Gastrointestinal problems
Yilmaz et al. (2007)	Turkey	Parallel, R, PC	Type 2 patients	M/F: 34	15	19	24	62.6 ± 6.6	61.5 ± 12	31.3 ± 3.7	28.2 ± 5.9	300	Control group	NR
Gao et al. (2007)	China	Crossover, R, PC	Type 2 patients	M/F: 16	16	16	4	49.4 ± 6.4	49.4 ± 6.4	NR	NR	50	Nateglinide	No side effect
Oyama et al. (2008)	Japan	Parallel, R, PC	Type 2 patients	M/F: 84	41	43	52	65 ± 6	63 ± 4	23.4 ± 2.5	23.1 ± 3.2	300	Control group	NR
Hasegawa et al. (2008)	Japan	Parallel, R, PC	Type 2 patients	M/F: 24	13	11	12	56.3 ± 6.5	56.1 ± 6.6	23.4 ± 3.3	23.5 ± 3.3	300	Control group	NR
Nijpels et al. (2008)	Netherlands	Parallel, R, PC, DB	Impaired glucose tolerance	M/F: 118	60	58	156	58.5 ± 7.9	56.5 ± 7	28.4 ± 3.9	29.5 ± 3.8	300	Placebo	Gastrointestinal problems
Pan et al. (2008)	China	Parallel, R, PC, DB	Type 2 patients	M/F: 661	220	441	24	51.9 ± 10.3	51.8 ± 10.1	25.8 ± 3.5	26.4 ± 3.6	300	Vildagliptin	NR
Derosa et al. (2009)	Italy	Parallel, R, PC, DB	Type 2 patients	M/F: 274	136	138	24	56 ± 6	56 ± 7	26.57 ± 0.7	26.85 ± 0.7	300	Pioglitazone	NR
Derosa et al. (2009)	Italy	Parallel, R, PC, DB	Type 2 patients	M/F: 103	52	51	15	55 ± 11	53 ± 9	26.7 ± 0.7	27.2 ± 0.9	300	Repaglinide	NR
Hanefeld et al, (2009)	Germany	Parallel, R, PC, DB	Type 2 patients	M/F: 87	42	45	16	62.33 ± 8.7	59.92 ± 10.05	31.02 ± 5.12	30.28 ± 3.7	300	Placebo	NR
Jayaram et al. (2010)	Indiana	Parallel, R, PC	Type 2 patients	M/F: 229	115	114	12	49.33 ± 7.7	49.01 ± 8.45	27.11 ± 1.77	27.3 ± 1.63	150	Control group	No side effect
Bao et al. (2010)	China	Parallel, R, PC	Type 2 patients	M/F: 46	24	22	8	54.7	52.6	25.28 ± 3.33	25.47 ± 2.99	100	Control group	No side effect
Koyasu et al. (2010)	Japan	Parallel, R, PC	Type 2 patients	M/F: 81	42	39	52	66.1 ± 8.6	66.5 ± 8	24.9 ± 2.7	24.5 ± 3.3	150	Control group	NR
Derosa et al. (2011)	Italy	Parallel, R, PC, DB	Type 2 patients	M/F: 188	96	92	28	56 ± 7	56 ± 7	26.6 ± 0.8	26.8 ± 0.9	300	Control group	NR
Rudovich et al. (2011) (B)	Germany	Crossover, R, PC, DB	Impaired glucose tolerance	M/F: 27	27	27	12	60.2 ± 1.8	60.2 ± 1.8	31.5 ± 4.6	31.5 ± 4.6	300	Placebo	NR
Rudovich et al. (2011) (C)	Germany	Crossover, R, PC, DB	Type 2 patients	M/F: 252	25	25	12	60.7 ± 9.4	60.7 ± 9.4	31.9 ± 5.5	31.9 ± 5.5	300	Placebo	NR
Wang et al. (2011)	Taiwan	Parallel, R, PC	Type 2 patients	M/F: 51	28	23	16	52.8 ± 8.2	54.7 ± 8.3	25.9 ± 3	25.3 ± 3.8	150	Glibenclamide	No side effect
Derosa et al. (2011)	Italy	Parallel, R, PC, DB	Type 2 patients	M/F: 188	96	92	24	56 ± 7	56 ± 7	26.6 ± 0.8	26.8 ± 0.9	300	Placebo	NR
Hirano et al. (2012)	Japan	Parallel, R, PC	Type 2 patients	M/F: 44	22	22	24	65 ± 10	65 ± 11	25 ± 3.9	24.9 ± 3.8	300	Control group	NR
Nakhaee et al. (2013)	Iran	Parallel, R, PC, DB	Type 2 patients	M/F: 40	19	21	20	30.3 ± 1.9	31.7 ± 2	30.3 ± 0.6	29.8 ± 0.5	300	Placebo	NR
Zheng et al. (2013)	China	Parallel, R, PC	Type 2 patients	M/F: 40	20	20	4	50.3 ± 10.3	49.8 ± 9.1	25.1 ± 3	24.7 ± 3.2	150	Nateglinide	NR
Patel et al. (2013)	Indiana	Parallel, R, PC, DB	Type 2 patients	M/F: 162	81	81	52	53.6 ± 11.1	53.6 ± 11.7	35.2 ± 7.3	35.3 ± 7.1	300	Placebo	NR
Wang et al. (2013)	China	Parallel, R, PC	Type 2 patients	M/F: 57	27	30	24	54.7 ± 8.9	55.89 ± 10.5	NR	NR	300	Gliclazide	NR
Li et al. (2013)	China	Parallel, R, PC	Type 2 patients	M/F: 39	20	19	12	58.6 ± 11.1	54.6 ± 8.6	25.9 ± 2.6	26.7 ± 2.9	150	Nateglinide	NR
Lee et al. (2014)	Korea	Parallel, R, PC	Type 2 patients	M/F: 121	59	62	24	58.36 ± 8.59	58.73 ± 10.09	24.7 ± 3.29	24.99 ± 3.09	300	Voglibose	Gastrointestinal problems
Sugihara et al. (2014)	Japan	Parallel, R, PC	Type 2 patients	M/F: 44	22	22	12	61.8 ± 13.7	66.6 ± 13	28.6 ± 2.7	28.7 ± 3.1	300	Control group	No side effect
Chen et al. (2014)	Taiwan	Parallel, R, PC	Type 2 patients	M/F: 51	28	23	16	53.7 ± 8.2	54.7 ± 8.3	25.6 ± 3.3	25.3 ± 3.8	150	Glibenclamide	NR
Yang et al. (2014)	China	Parallel, R, PC	Type 2 patients	M/F: 711	361	350	48	50.6 ± 9.2	50.2 ± 9.3	25.5 ± 2.7	25.7 ± 2.6	300	Metformin	Gastrointestinal problems, infections, and infestations, metabolism and nutrition disorders, nervous system disorders, musculoskeletal and connective tissue disorders
Su et al. (2015)	China	Parallel, R, PC	Type 2 patients	M/F: 95	59	36	4	55.7 ± 11	56.5 ± 10.2	27.21 ± 4.25	26.73 ± 3.11	150	Control group	NR
Zhou et al. (2015)	China	Parallel, R, PC	Type 2 patients	M/F: 103	52	51	2	53.8 ± 9.3	53.9 ± 10.2	24.88 ± 2.69	25.15 ± 2.92	150	Nateglinide	NR
Sun et al. (2016)	China	Parallel, R, PC	Type 2 patients	M/F: 108	54	54	24	53 ± 8	52 ± 6	27.07 ± 1.97	27.02 ± 1.85	300	Metformin	Abdominal distension and diarrhea
Pan et al. (2016)	China	Parallel, R, PC	Type 2 patients	M/F: 762	382	380	48	50.59 ± 9.19	50.44 ± 9.34	25.6 ± 2.57	25.67 ± 2.58	300	Metformin	NR
Yun et al. (2016)	China	Parallel, R, PC	Impaired glucose tolerance	M/F: 135	67	68	120	62.24 ± 5.16	61.62 ± 4.58	26.05 ± 3.24	25.82 ± 2.45	150	Control group	Gastrointestinal problems
Chen et al. (2016)	Taiwan	Parallel, R, PC	Type 2 patients	M/F: 60	30	30	24	67.2 ± 7.6	66.3 ± 8.8	30.1 ± 18.4	26 ± 3.4	150	Pioglitazone	NR
Li et al. (2016)	China	Parallel, R, PC, DB	Type 2 patients	M/F: 38	15	23	24	57 ± 6.7	56 ± 9.71	25.47 ± 2.61	25.67 ± 2.74	150	SZ-A	Gastrointestinal problems
Ziaee et al. (2017)	Iran	Crossover, R, PC	Type 1 patients	M/F: 40	40	40	24	19.31 ± 1.25	19.31 ± 1.25	23.96 ± 1.7	23.21 ± 1.4	300	Metformin	NR
Shi et al. (2017)	China	Parallel, R, PC	Type 2 patients	M/F: 36	18	18	12	38.7 ± 10.3	44.4 ± 11.1	31.13 ± 2.54	31.48 ± 3.09	300	Control group	NR
Du et al. (2017)	China	Parallel, R, PC	Type 2 patients	M/F: 481	243	238	24	56.5 ± 10.81	54.7 ± 10.51	26.3 ± 3.49	26.4 ± 3.47	300	Saxagliptin	NR
Wu et al. (2017)	China	Parallel, R, PC, DB	Type 2 patients	M/F: 272	80	192	16	57.93 ± 10.25	55.96 ± 10.06	24.66 ± 2.8	25.03 ± 267	150	Metformin	NR
Yang et al. (2019)	Korea	Parallel, R, PC, DB	Type 2 patients	M/F: 131	66	65	24	60.89 ± 8.9	56.55 ± 10.6	25.05 ± 4	25.39 ± 3.6	300	Control group	NR
Sanjari et al. (2019) (A)	Iran	Parallel, R, PC, TB	Type 2 patients	M/F: 16	8	8	2	52.4 ± 5.5	47.8 ± 8.1	29.8 ± 5.1	26.8 ± 4.3	100	Placebo	NR
Sanjari et al. (2019) (B)	Iran	Parallel, R, PC, TB	Type 2 patients	M/F: 14	7	7	2	40.7 ± 8.7	33.2 ± 6.6	31.1 ± 7.8	25.8 ± 4.6	100	Placebo	NR
Mo et al. (2019)	China	Parallel, R, PC	Type 2 patients	M/F: 70	34	36	52	51.38 ± 9.61	51.31 ± 9.02	24.64 ± 2.83	25.04 ± 2.68	300	Metformin	NR
Li et al. (2019)	China	Parallel, R, PC	Type 2 patients	M/F: 144	72	72	52	68.41 ± 4.46	68.92 ± 4.75	NR	NR	300	Control group	NR
Gao et al. (2020)	China	Parallel, R, PC	Type 2 patients	M/F: 124	62	62	12	63 ± 5.25	60 ± 5.5	25.6 ± 2.6	26.42 ± 2.76	150	Metformin	Gastrointestinal problems
Ren et al. (2022)	China	Parallel, R, PC	Type 2 patients	M/F: 88	48	40	15	51.21 ± 6.53	50.53 ± 6.96	22.98 ± 2.57	23.26 ± 2.12	150	Metformin	Edema, nausea, gastrointestinal discomfort, and hypoglycemia
Gao et al. (2022)	China	Parallel, R, PC	Type 2 patients	M/F:1,088	363	725	16	60.5 ± 7.2	59.3 ± 7.5	25.8 ± 3	25.7 ± 3.4	300	Alogliptin	Constipation, nausea, diarrhea, and flatulence

IG, intervention group; CG, control group; DB, double-blinded; SB, single-blinded; PC, placebo-controlled; CO, controlled; RA, randomized; NR, not reported; F, Female; M, Male; NR, not reported.

### Study characteristics

The publication years of the studies ranged from 1982 to 2022 and originated in China (21, 44, 58, 60, 70, 77, 94, 99, 105–107, 111–114, 116, 117, 119, 120, 123–127, 129, 132), New Zealand (45), Germany (56, 62–64, 74–76, 78, 84, 89, 97, 100, 130, 131), Australia (47), United States (48, 49), Canada (50, 51), Japan (46, 55, 71, 82, 85, 88, 91, 92, 98, 103, 110, 137), Turkey (52, 53, 66, 90), Italy (59, 69, 86, 95, 96, 101, 128), Mexico (65), United Kingdom (136), Sweden (87), Indiana (107), Taiwan (79, 81, 102, 109, 115), Iran (118, 121), Korea (108, 122), Spain (54), Netherlands (61, 83, 93), France (68), Thailand (72), Brazil (73), and Sweden (87). We showed the study design characteristics in Table 1. The WMD and 95%CI of TG (mg/dL), TC (mg/dL), LDL (mg/dL), HDL (mg/dL), FBG (mg/dL), insulin (pmol/L), HbA1c (%), HOMA-IR, SBP (mmHg), DBP (mmHg), CRP (mg/L), IL-6 (pg/mL), TNF-α (pg/mL), adiponectin (ng/mL), leptin (ng/mL), weight (kg), BMI (kg/m^2^), WC (cm), ALT (U/L), AST (U/L), and ALP (U/L) and their changes are presented in Supplementary Figures S2A–U, respectively.

There was 84 parallel (21, 44, 46, 48–52, 54–56, 58–60, 62–64, 66–71, 73–85, 87–99, 101, 103–117, 119–128) and 11 cross-over studies (45, 47, 53, 57, 61, 65, 72, 86, 100, 118, 129). The mean age and baseline BMI of included studies ranged from 19.31 to 69.7 years and 21.1 to 35.2 kg/m^2^ in the intervention group, respectively. The treatment duration of included studies ranged from 2 to 156 weeks. The daily dosage of ACB treatment ranged from 75 to 600 mg. One study included only female participants and 94 included both sexes.

Studies included participants with T2D (21, 44–50, 52–60, 62–76, 78–92, 94–113, 115–117, 119–127, 129–136), type 1 diabetes mellitus (T1D) (61, 118), and impaired glucose tolerance (51, 77, 93, 100, 114, 137).

In the investigation by Rudovich et al. (100), two types of participants (IGT and T2D subjects) participated both females and males so two arms were considered for this study. Furthermore, Sanjari et al. (121) had two types of participants [healthy subjects (*n* = 14) and T2D patients (*n* = 14)] participated in both females and males so we considered two arms for this study. In the investigation by Fischer et al. (63), one type of participant (T2D) participated in both females and males with different dose interventions (75, 150, 300, and 600 mg/d) so four arms were considered for this study.

Out of the 95 RCTs, there were 81 effect sizes for the effect of ACB treatment on FBG (mg/dL), 39 effect sizes on serum insulin (pmol/L), 77 effect sizes on serum HbA1c (%), 17 effect sizes on HOMA-IR, 36 effect sizes on body weight, 34 effect sizes on BMI, 6 effect sizes on WC, 9 effect sizes on ALT (U/L), 7 effect sizes on AST (U/L), and 3 effect sizes on ALP (U/L). Out of the 95 RCTs, there were 6, 7, 3, 5, and 3 effect sizes for CRP, IL-6, TNF-α, adiponectin, and leptin, respectively. Furthermore, there were 59, 54, 43, 53, 29, and 29 effect sizes for TG, TC, LDL, HDL, SBP, and DBP, respectively.

### Adverse events

Information on adverse effects was mentioned in the studies of Soonthornpun et al. (57) (mild and tolerable gastrointestinal problems), Coniff et al. (49) (abdominal pain, nausea, diarrhea, and flatulence), Costa et al. (54) (constipation, nausea, diarrhea, and flatulence), Fischer et al. (63) (flatulence and meteorism), Josse et al. (80) (constipation, nausea, diarrhea, and flatulence), Li et al. (113) (gastrointestinal problems), Lin et al. (79) (gastrointestinal problems), Nijpels et al. (93) (gastrointestinal problems), Van de laar et al. (83) (flatulence, diarrhea, abdominal pain or nausea, and headache), Sels et al. (61) (flatulence, diarrhea, and abdominal pain), Ren et al. (124) (edema, nausea, gastrointestinal discomfort, and hypoglycemia), Gao et al. (125) (constipation, nausea, diarrhea, and flatulence), Yang et al. (21) (gastrointestinal problems, infections and infestations, metabolism and nutrition disorders, nervous system disorders, and musculoskeletal and connective tissue disorders), Coniff et al. (48) (diarrhea and flatulence), Gao et al. (123) (gastrointestinal problems), Goke et al. (76) (increased liver enzymes, cardiac failure, and gastrointestinal problems), Guagnano et al. (59) (flatulence, abdominal cramps, and diarrhea), Ko et al. (70) (flatulence, diarrhea, and abdominal colic), Lee et al. (108) (gastrointestinal problems), Lopez-Alvarenga (65) (gastrointestinal problems), Pan et al. (77) (gastrointestinal problems), Salman et al. (66) (flatulence, abdominal pain, and diarrhea), Schnell et al. (89) (gastrointestinal problems), Sun et al. (116) (abdominal distension and diarrhea), Wang et al. (102) (abdominal distension and low back pain), Yun et al. (114) (gastrointestinal problems), and Hanefeld et al. (130) (flatulence, abdominal distension, and diarrhea). The adverse events are presented in Table 1.

## Qualitative data assessment

We assessed the qualitative data based on the Cochrane risk-of-bias assessment tool. Six studies had a moderate risk of bias (73, 83, 93, 97, 120, 133), and 89 studies had a high risk of bias (21, 44–72, 74–82, 84–92, 96, 98–119, 121–132, 134–137). The qualitative data assessment is presented in Table 2.

**Table 2:** Quality assessment (a summary of the risk of bias according to Cochrane criteria).

Studies	Random sequence generation	Allocation concealment	Selective reporting	Other sources of bias	Blinding (participants and personnel)	Blinding (outcome assessment)	Incomplete outcome data	General risk of bias
Akazawa et al. (1982)	U	H	H	H	H	H	L	H
Scott et al. (1984)	L	H	H	H	H	H	L	H
Hanefeld et al. (1991)	L	H	H	H	L	U	L	H
Hotta et al. (1993)	L	H	H	H	L	U	L	H
Jenney et al. (1993)	L	H	H	H	L	U	L	H
Coniff et al. (1994)	L	H	H	H	L	U	H	H
Hoffman et al. (1994)	L	H	H	H	L	U	H	H
Coniff et al. (1995)	L	H	H	H	L	U	H	H
Wolever et al. (1995)	L	H	H	H	L	U	L	H
Chiasson et al. (1996)	L	H	H	H	L	U	L	H
Bayraktar et al. (1996)	U	H	H	H	H	H	L	H
Noda et al. (1997)	L	H	H	H	H	H	L	H
Hoffmann et al. (1997)	L	H	H	H	L	U	L	H
Costa et al. (1997)	L	H	H	H	L	U	L	H
Chan et al. (1998)	L	H	H	H	L	U	H	H
Guagnano et al. (1998)	L	H	H	H	H	H	L	H
Bayraktar et al. (1998)	U	H	H	H	H	H	L	H
Soonthornpun et al. (1998)	L	H	H	H	L	U	L	H
Lam et al. (1998)	L	H	H	H	L	U	H	H
Buchanan et al. (1998)	U	H	H	H	H	H	L	H
Sels et al. (1998)	L	H	H	H	H	H	L	H
Fischer et al. (1998)	L	H	H	H	L	U	L	H
Standl et al. (1999)	L	H	H	H	H	H	L	H
López‐Alvarenga et al. (1999)	L	H	H	H	L	U	L	H
Holman et al. (1999)	U	H	H	H	L	U	H	H
Salman et al. (2000)	L	H	H	H	H	H	L	H
Meneilly et al. (2000)	L	H	H	H	L	U	H	H
Halimi et al. (2000)	L	H	H	H	L	U	L	H
Ko et al. (2001)	L	H	H	H	H	H	L	H
Gentile et al. (2001)	L	H	H	H	L	U	L	H
Takei et al. (2001)	L	H	H	H	H	H	L	H
Hanefeld et al. (2002)	L	H	H	H	L	U	L	H
Vichayanrat et al. (2002)	L	H	H	H	H	H	L	H
Rosenthal et al. (2002)	L	H	H	H	H	H	L	H
Göke et al. (2002)	L	L	H	H	H	H	H	H
Rosenbaum et al. (2002)	L	H	L	H	L	U	L	M
Fischer et al. (2003)	L	H	H	H	L	U	L	H
Pan et al. (2003)	L	H	H	H	L	H	L	H
Josse et al. (2003)	L	H	H	H	L	U	H	H
Lin et al. (2003)	L	H	H	H	L	U	L	H
Bachmann et al. (2003)	L	H	H	H	L	U	L	H
Hwu et al. (2003)	L	H	H	H	L	U	H	H
Yajima et al. (2004)	L	H	H	H	H	H	L	H
Watanabe et al. (2004)	L	H	H	H	H	H	L	H
van de Laar et al. (2004)	L	L	H	H	L	U	L	M
Göke et al. (2004)	L	U	H	H	H	H	H	H
Gentile et al. (2005)	U	H	H	H	L	U	H	H
Inoue et al. (2006)	U	H	H	H	H	H	L	H
Suzuki et al. (2006)	L	H	H	H	H	H	L	H
Wagner et al. (2006)	L	H	H	H	H	h	L	H
Schnell et al. (2007)	L	H	H	H	L	U	L	H
Yilmaz et al. (2007)	L	H	H	H	H	H	L	H
Gao et al. (2007)	L	H	L	H	H	H	L	H
Oyama et al. (2008)	L	H	H	H	H	H	H	H
Hasegawa et al. (2008)	L	H	H	H	H	H	L	H
Nijpels et al. (2008)	L	L	H	H	L	U	L	M
Pan et al. (2008)	L	L	H	H	L	U	L	H
Derosa et al. (2009)	L	H	H	H	L	U	L	H
Derosa et al. (2009)	L	H	H	H	L	U	L	H
Hanefeld et al, (2009)	L	L	H	H	L	U	L	M
Jayaram et al. (2010)	L	H	H	H	H	H	L	H
Bao et al. (2010)	L	L	H	H	H	H	L	H
Koyasu et al. (2010)	L	H	H	H	H	H	L	H
Derosa et al. (2011)	L	H	H	H	L	U	L	H
Rudovich et al. (2011)	L	H	H	H	L	U	L	H
Wang et al. (2011)	L	L	H	H	H	H	L	H
Derosa et al. (2011)	L	H	H	H	L	U	L	H
Hirano et al. (2012)	L	H	H	H	H	H	L	H
Nakhaee et al. (2013)	L	L	H	H	L	U	H	M
Zheng et al. (2013)	L	H	H	H	H	H	L	H
Patel et al. (2013)	L	H	H	H	L	U	H	H
Wang et al. (2013)	L	H	H	H	H	H	L	H
Li et al. (2013)	L	H	H	H	H	H	L	H
Lee et al. (2014)	L	H	H	H	H	H	H	H
Sugihara et al. (2014)	L	L	H	H	H	H	L	H
Chen et al. (2014)	L	L	H	H	H	H	L	H
Yang et al. (2014)	U	L	H	H	H	H	H	H
Su et al. (2015)	L	H	H	H	H	H	L	H
Zhou et al. (2015)	L	H	L	H	H	H	L	H
Sun et al. (2016)	L	L	H	H	H	H	L	H
Pan et al. (2016)	L	H	H	H	H	H	L	H
Yun et al. (2016)	L	L	H	H	H	H	L	H
Chen et al. (2016)	U	L	H	H	H	H	H	H
Li et al. (2016)	L	H	H	H	L	U	H	H
Ziaee et al. (2017)	L	H	H	H	H	H	L	H
Shi et al. (2017)	L	H	H	H	H	H	L	H
Du et al. (2017)	L	L	H	H	H	H	L	H
Wu et al. (2017)	L	L	H	H	L	U	L	M
Yang et al. (2019)	L	L	H	H	L	U	H	H
Sanjari et al. (2019)	L	H	H	H	L	L	L	H
Mo et al. (2019)	L	H	H	H	H	H	L	H
Li et al. (2019)	L	H	H	H	H	H	L	H
Gao et al. (2020)	L	L	H	H	H	H	H	H
Ren et al. (2022)	L	H	H	H	H	H	L	H
Gao et al. (2022)	L	H	H	H	H	H	L	H

General low risk < 2 high risk, general moderate risk = 2 high risk, general high risk > 2 high risk.

### Effect of ACB intake on TG (mg/dL) and subgroup analysis

**Figure 2:**
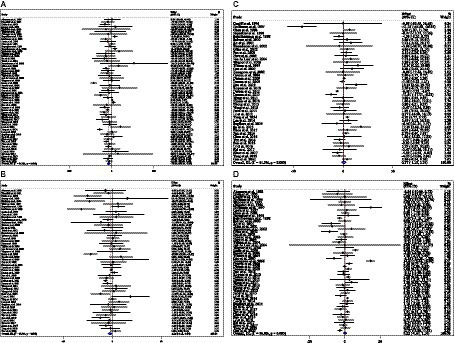
Forest plot detailing weighted mean difference and 95% confidence intervals (CIs) for the effect of acarbose consumption on **(A)** TG (mg/dL); **(B)** TC (mg/dL); **(C)** LDL (mg/dL); and **(D)** HDL (mg/dL).

Combining 59 effect sizes from 59 studies (*n* total = 6,214, *n* IG = 3,111, *n* CG = 3,103) has shown that ACB treatment had a significant reduction effect on TG (mg/dL) (WMD = −13.89 mg/dL; 95%CI: −20.69, −7.09; *p* < 0.001; *I*^2^ = 86.0%, *p* < 0.001) (Figure 2A). ACB consumption lowered TG in all subgroups except those with trials lasting less than 24 weeks, according to the subgroup analyses (WMD = −9.65 mg/dL; 95%CI: −22.59, 3.29; *p* = 0.144; *I*^2^ = 88.3%, *p* < 0.001) (Figure 2A). ACB intake had a reduction effect on TG (mg/dL) in prediabetes (WMD = −22.17 mg/dL; 95%CI: −43.90 to −0.45; *p* = 0.045; *I*^2^ = 35.5%, *p* = 0.212) and T2D patients (WMD = −13.05 mg/dL; 95%CI: −19.61 to −6.48; *p* < 0.001; *I*^2^ = 82.9%, *p* < 0.001). ACB consumption lowered TG in Eastern (WMD = −14.37 mg/dL; 95%CI: −21.94 to −6.81; *p* < 0.001; *I*^2^ = 89.0%, *p* < 0.001) and Western status (WMD = −14.15 mg/dL; 95%CI: −27.06 to −1.25; *p* = 0.031; *I*^2^ = 22.6%, *p* = 0.203). Between-study heterogeneity disappeared in studies with prediabetes participants (*I*^2^ = 35.5%, *p* = 0.212), participants with Western status (*I*^2^ = 22.6%, *p* = 0.203), and with an intervention dose of <300 mg (*I*^2^ = 15.6%, *p* = 0.271) (Table 3).

**Table 3:** Subgroup analyses of acarbose on diabetes in patients with T2D and impaired glucose tolerance patients.

	No	WMD (95%CI)	*p*-value	Heterogeneity		
				*p* heterogeneity	*I*^2^ (%)	*p* between sub-groups
Subgroup analyses of acarbose on serum TG (mg/dL)						
Overall effect	59	−13.89 (−20.69, −7.09)	<0.001	<0.001	86.0	
Baseline TG (mg/dL)						
<150	22	−9.78 (−17.32, −2.23)	0.011	<0.001	76.2	0.302
≥150	37	−16.39 (−26.42, −6.35)	0.001	<0.001	84.8	
Trial duration (week)						
<24	28	−9.65 (−22.59, 3.29)	0.144	<0.001	88.3	0.363
≥24	31	−16.70 (−24.67, −8.73)	<0.001	<0.001	83.2	
Intervention dose (mg/day)						
<300	17	−17.36 (−24.18, −10.55)	<0.001	0.271	15.6	0.489
≥300	42	−13.56 (−21.92, −5.19)	0.001	<0.001	89.2	
Glycemic state						
Prediabetes	3	−22.17 (−43.90, −0.45)	0.045	0.212	35.5	0.431
T2D	45	−13.05 (−19.61, −6.48)	<0.001	<0.001	82.9	
Ethnic status						
Eastern	44	−14.37 (−21.94, −6.81)	<0.001	<0.001	89.0	0.977
Western	15	−14.15 (−27.06, −1.25)	0.031	0.203	22.6	
Subgroup analyses of acarbose on serum TC (mg/dL)						
Overall effect	54	−2.26 (−4.18, −0.34)	0.021	<0.001	68.0	
Baseline TC (mg/dL)						
<200	24	−3.07 (−5.72, −0.41)	0.023	<0.001	75.7	0.426
≥200	30	−1.49 (−4.32, 1.33)	0.301	0.001	51.7	
Trial duration (week)						
<24	27	−0.25 (−3.34, 2.84)	0.872	<0.001	58.2	0.074
≥24	27	−3.88 (−6.40, −1.37)	0.002	<0.001	72.2	
Intervention dose (mg/day)						
<300	16	0.35 (−2.14, 2.84)	0.783	0.497	0.0	0.038
≥300	38	−3.25 (−5.56, −0.94)	0.006	<0.001	74.5	
Glycemic state						
Prediabetes	2	−4.15 (−9.36, 1.05)	0.118	0.299	7.4	0.390
T2D	41	−1.70 (−3.72, 0.31)	0.098	<0.001	64.9	
Ethnic status						
Eastern	42	−2.29 (−4.29, −0.30)	0.024	<0.001	68.9	0.883
Western	12	−2.87 (−10.25, 4.50)	0.446	<0.001	67.1	
Subgroup analyses of acarbose on serum LDL (mg/dL)						
Overall effect	43	0.77 (−1.19, 2.73)	0.440	<0.001	81.3	
Baseline LDL (mg/dL)						
<100	6	−2.31 (−7.79, 3.16)	0.407	0.005	69.9	0.216
≥100	37	1.39 (−0.73, 3.51)	0.200	<0.001	82.2	
Trial duration (week)						
<24	20	2.49 (−0.01, 5.00)	0.051	0.028	41.4	0.107
≥24	23	−0.54 (−3.25, 2.17)	0.696	<0.001	88.3	
Intervention dose (mg/day)						
<300	13	3.79 (0.76, 6.83)	0.014	0.067	40.0	0.026
≥300	30	−0.57 (−2.92, 1.78)	0.635	<0.001	85.0	
Glycemic state						
Prediabetes	2	−7.61 (−18.77, 3.54)	0.181	0.106	61.8	0.142
T2D	37	0.90 (−1.16, 2.97)	0.393	<0.001	82.1	
Ethnic status						
Eastern	37	1.00 (−0.95, 2.95)	0.315	<0.001	80.8	0.441
Western	6	−4.93 (−19.92, 10.04)	0.518	<0.001	86.3	
Subgroup analyses of acarbose on serum HDL (mg/dL)						
Overall effect	53	0.21 (−0.66, 1.10)	0.629	<0.001	86.8	
Baseline HDL (mg/dL)						
<40	7	−0.09 (−1.45, 1.26)	0.890	0.991	0.0	0.709
≥40	46	0.22 (−0.73, 1.17)	0.651	<0.001	88.5	
Trial duration (week)						
<24	26	0.01 (−2.00, 2.03)	0.988	<0.001	91.7	0.700
≥24	27	0.44 (−0.33, 1.22)	0.266	<0.001	67.2	
Intervention dose (mg/day)						
<300	15	−1.36 (−2.49, −0.24)	0.017	0.040	42.7	0.004
≥300	38	0.86 (−0.17, 1.90)	0.102	<0.001	87.8	
Glycemic state						
Prediabetes	3	0.78 (−3.56, 5.13)	0.725	0.681	0.0	0.976
T2D	40	0.71 (−0.22, 1.64)	0.134	<0.001	87.1	
Ethnic status						
Eastern	42	0.18 (−0.74, 1.11)	0.694	<0.001	88.4	0.774
Western	11	0.74 (−2.96, 4.45)	0.694	<0.001	73.1	
Subgroup analyses of acarbose on serum FBG (mg/dL)						
Overall effect	81	−3.55 (−6.29, −0.81)	0.011	<0.001	93.1	
Baseline FBG (mg/dL)						
<100	6	−8.78 (−15.98, −1.58)	0.017	<0.001	87.7	0.176
≥100	74	−3.41 (−6.34, −0.48)	0.022	<0.001	93.3	
Trial duration (week)						
<24	39	−3.03 (−6.80, 0.72)	0.114	<0.001	87.7	0.729
≥24	42	−3.99 (−7.89, −0.09)	0.045	<0.001	95.0	
Intervention dose (mg/day)						
<300	19	0.57 (−4.58, 5.72)	0.828	<0.001	87.0	0.082
≥300	62	−4.82 (−8.06, −1.58)	0.004	<0.001	94.0	
Glycemic state						
Prediabetes	6	−8.78 (−15.98, −1.58)	0.017	<0.001	87.7	0.176
T2D	74	−3.41 (−6.34, −0.48)	0.022	<0.001	93.3	
Ethnic status						
Eastern	55	−2.74 (−6.03, 0.54)	0.102	<0.001	94.7	0.348
Western	26	−5.54 (−10.36, −0.71)	0.024	<0.001	81.0	
Subgroup analyses of acarbose on serum insulin (pmol/L)						
Overall effect	39	−6.73 (−10.37, −3.10)	<0.001	<0.001	87.3	
Trial duration (week)						
<24	20	−10.42 (−18.29, −2.55)	0.009	<0.001	85.0	0.168
≥24	19	−4.06 (−8.51, 0.39)	0.074	<0.001	89.6	
Intervention dose (mg/day)						
<300	7	−7.78 (−13.66, −1.90)	0.009	<0.001	8.0	0.738
≥300	32	−6.56 (−10.59, −2.53)	0.001	<0.001	89.3	
Glycemic state						
Prediabetes	5	−10.97 (−22.69, 0.75)	0.067	0.500	0.0	0.531
T2D	31	−7.03 (−10.84, −3.21)	<0.001	<0.001	89.6	
Ethnic status						
Eastern	21	−5.26 (−9.50, −1.02)	0.015	<0.001	91.9	0.196
Western	18	−11.65 (−20.38, −2.93)	0.009	<0.001	63.4	
Subgroup analyses of acarbose on serum HbA1c (%)						
Overall effect	77	−0.32 (−0.45, −0.20)	<0.001	<0.001	96.3	
Trial duration (week)						
<24	34	−0.27 (−0.49, −0.05)	0.018	<0.001	94.2	0.447
≥24	43	−0.37 (−0.53, −0.21)	<0.001	<0.001	97.0	
Intervention dose (mg/day)						
<300	17	−0.13 (−0.29, 0.03)	0.132	<0.001	88.0	0.024
≥300	60	−0.39 (−0.54, −0.23)	<0.001	<0.001	96.9	
Glycemic state						
Prediabetes	2	0.26 (−0.11, 0.65)	0.171	0.709	0.0	0.006
T2D	67	−0.29 (−0.43, −0.16)	<0.001	<0.001	96.3	
Ethnic status						
Eastern	50	−0.28 (−0.43, −0.13)	<0.001	<0.001	97.1	0.326
Western	27	−0.41 (−0.61, −0.21)	<0.001	<0.001	89.6	
Subgroup analyses of acarbose on HOMA-IR						
Overall effect	17	−0.10 (−0.57, 0.36)	0.670	<0.001	95.9	
Trial duration (week)						
<24	8	−0.15 (−1.07, 0.75)	0.736	<0.001	94.5	0.813
≥24	9	−0.02 (−0.59, 0.53)	0.922	<0.001	96.2	
Intervention dose (mg/day)						
<300	5	0.21 (−0.51, 0.94)	0.565	0.003	75.1	0.324
≥300	12	−0.25 (−0.81, 0.31)	0.386	<0.001	97.0	
Subgroup analyses of acarbose on SBP (mmHg)						
Overall effect	29	−1.29 (−2.44, −0.15)	0.027	<0.001	86.7	
Baseline SBP (mmHg)						
<130	10	0.40 (0.11, 0.69)	0.006	0.476	0.0	0.005
≥130	18	−2.49 (−4.48, −0.50)	0.014	<0.001	90.4	
Trial duration (week)						
<24	13	−3.22 (−6.48, 0.04)	0.053	<0.001	89.8	0.078
≥24	16	−0.13 (−1.21, 0.95)	0.815	<0.001	79.5	
Intervention dose (mg/day)						
<300	8	−0.17 (−2.94, 2.59)	0.902	0.001	72.1	0.371
≥300	21	−1.57 (−2.88, −0.26)	0.019	<0.001	88.9	
Ethnic status						
Eastern	23	−1.28 (−2.50, −0.06)	0.040	<0.001	88.2	0.773
Western	6	−1.98 (−6.64, 2.67)	0.403	<0.001	78.5	
Subgroup analyses of acarbose on DBP (mmHg)						
Overall effect	29	0.02 (−0.41, 0.45)	0.925	0.013	40.8	
Baseline DBP (mmHg)						
<80	12	−0.17 (−0.52, 0.18)	0.350	0.520	0.0	0.302
≥80	16	0.22 (−0.43, 0.88)	0.504	0.029	44.3	
Trial duration (week)						
<24	13	−0.49 (−1.07, 0.09)	0.097	0.720	0.0	0.076
≥24	16	0.23 (0.04, 0.73)	0.408	0.006	53.3	
Intervention dose (mg/day)						
<300	8	−1.13 (−1.86, −0.41)	0.002	0.397	4.2	<0.001
≥300	21	0.38 (0.04, 0.73)	0.028	0.280	13.8	
Ethnic status						
Eastern	23	−0.01 (−0.48, 0.45)	0.955	0.004	49.1	0.600
Western	6	0.38 (−1.01, 1.77)	0.593	0.558	0.0	
Subgroup analyses of acarbose on serum CRP (mg/L)						
Overall effect	6	−0.15 (−0.37, 0.07)	0.185	0.021	62.5	
Trial duration (week)						
<24	2	0.10 (−0.54, 0.73)	0.760	0.435	0.0	0.421
≥24	4	−0.18 (−0.42, 0.06)	0.150	0.009	74.3	
Subgroup analyses of acarbose on serum IL-6 (pg/mL)						
Overall effect	7	−0.20 (−0.50, 0.09)	0.179	0.009	64.7	
Trial duration (week)						
<24	4	−0.47 (−1.50, 0.55)	0.367	0.008	74.6	0.608
≥24	3	−0.19 (−0.44, 0.05)	0.124	0.076	61.2	
Ethnic status						
Eastern	5	−0.26 (−0.65, 0.12)	0.183	0.008	70.9	0.645
Western	2	−0.08 (−0.76, 0.60)	0.817	0.078	67.9	
Subgroup analyses of acarbose on serum TNF-α (pg/mL)						
Overall effect	3	−2.70 (−5.25, −0.16)	0.037	0.064	63.7	
Subgroup analyses of acarbose on serum adiponectin (ng/mL)						
Overall effect	5	0.95 (−0.22, 2.13)	0.112	0.005	72.9	
Subgroup analyses of acarbose on serum leptin (ng/mL)						
Overall effect	3	−1.58 (−2.82, −0.35)	0.012	0.523	0.0	
Subgroup analyses of acarbose on weight (kg)						
Overall effect	36	−1.25 (−1.79, −0.75)	<0.001	<0.001	56.7	
Trial duration (week)						
<24	16	−1.93 (−3.31, −0.54)	0.006	0.005	54.6	0.224
≥24	20	−1.01 (−1.50, −0.53)	<0.001	0.002	54.0	
Intervention dose (mg/day)						
<300	6	−1.58 (−2.43, −0.73)	<0.001	0.847	0.0	0.517
≥300	30	−1.24 (−1.82, −0.66)	<0.001	<0.001	62.4	
Glycemic state						
Prediabetes	2	−1.63 (−4.97, 1.70)	0.338	0.986	0.0	0.797
T2D	27	−1.18 (−1.72, −0.65)	<0.001	<0.001	57.5	
Ethnic status						
Eastern	23	−1.01 (−1.39, −0.62)	<0.001	0.186	20.6	0.375
Western	13	−1.71 (−3.23, −0.19)	0.027	<0.001	76.2	
Subgroup analyses of acarbose on BMI (kg/m^2^)						
Overall effect	34	−0.64 (−0.92, −0.37)	<0.001	<0.001	91.9	
Trial duration (week)						
<24	20	−0.96 (−1.56, −0.37)	0.001	<0.001	94.1	0.041
≥24	14	−0.30 (−0.52, −0.07)	0.009	<0.001	81.1	
Intervention dose (mg/day)						
<300	9	−0.24 (−0.59, 0.10)	0.171	0.016	57.5	0.033
≥300	25	−0.77 (−1.11, −0.43)	<0.001	<0.001	93.2	
Glycemic state						
Prediabetes	2	−0.46 (−2.20, 1.26)	0.596	0.945	0.0	0.831
T2DM	28	−0.65 (−0.95, −0.36)	<0.001	<0.001	92.4	
Ethnic status						
Eastern	28	−0.50 (−0.75, −0.24)	<0.001	<0.001	90.6	0.497
Western	6	−1.36 (−3.83, 1.11)	0.280	<0.001	94.0	
Subgroup analyses of acarbose on WC (cm)						
Overall effect	6	−1.55 (−3.14, 0.04)	0.056	0.019	62.9	
Intervention dose (g/day)						
<24	3	−3.55 (−7.52, 0.42)	0.080	0.036	70.0	0.130
≥24	3	−0.44 (−1.12, 0.23)	0.203	0.852	0.0	
Subgroup analyses of acarbose on ALT (U/L)						
Overall effect	9	0.76 (−0.31, 1.85)	0.164	<0.001	92.2	
Trial duration (week)						
<24	5	1.91 (−1.01, 4.84)	0.200	0.068	54.2	0.420
≥24	4	−0.01 (−3.70, 3.66)	0.992	0.039	64.2	
Intervention dose (mg/day)						
<300	4	4.53 (0.71, 8.36)	0.020	0.338	11.0	0.043
≥300	5	0.41 (−0.69, 1.53)	0.460	<0.001	95.8	
Ethnic status						
Eastern	7	0.76 (−0.37, 1.89)	0.190	<0.001	94.0	0.957
Western	2	0.92 (−4.82, 6.66)	0.753	0.068	69.9	
Subgroup analyses of acarbose on AST(U/L)						
Overall effect	7	−0.57 (−2.45, 1.30)	0.550	<0.001	99.3	
Trial duration (week)						
<24	3	0.17 (−5.04, 5.39)	0.948	<0.001	88.9	0.832
≥24	4	−0.41 (−1.81, 0.99)	0.568	0.115	49.4	
Intervention dose (mg/day)						
<300	3	1.81 (−1.71, 5.34)	0.313	0.095	57.6	0.100
≥300	4	−1.69 (−3.96, 0.57)	0.143	<0.001	99.6	
Subgroup analyses of acarbose on ALP(U/L)						
Overall effect	3	1.97 (−5.67, 9.61)	0.613	0.544	0.0	

ALP, alkaline phosphatase; ALT, alanine transaminase; AST, aspartate transaminase; BMI, body mass index; CI, confidence interval; CRP, c-reactive protein; FBG, fasting blood glucose; HbA1c, hemoglobin A1c; HDL, high-density lipoprotein; HOMA-IR, homeostatic model assessment for insulin resistance; LDL, low-density lipoprotein; DBP, diastolic blood pressure; SBP, systolic blood pressure; TC, total cholesterol, TG, triglyceride; WC, waist circumference; WMD, weighted mean differences; IL-6, interleukin 6.

### Effect of ACB intake on TC (mg/dL) and subgroup analysis

In total, 54 effect sizes from 54 trials (*n* total = 4,954, *n* IG = 2,489, *n* CG = 2,465) were considered in this analysis. After consuming ACB, pooled effect sizes showed a substantial decrease in TC (mg/dL) (WMD = −2.26 mg/dL; 95%CI: −4.18, −0.34; *p* = 0.021; *I*^2^ = 68.0%, *p* < 0.001) (Figure 2B). ACB significantly impacted TC in high-dose interventions (≥300 mg/day), according to the subgroup analyses (WMD = −3.25 mg/dL; 95%CI: −5.56, −0.94; *p* = 0.006; *I*^2^ = 74.5%, *p* < 0.001), and in studies with ≥24 weeks of intervention (WMD = −3.88 mg/dL; 95%CI: −6.40, −1.37; *p* = 0.002; *I*^2^ = 72.2%, *p* < 0.001) (Table 3). Other subgroup analyses based on health status and baseline TC also showed that ACB significantly reduced the TC in individuals with baseline TC < 200 (WMD = −3.07 mg/dL; 95%CI: −5.72, −0.41; *p* = 0.023; *I*^2^ = 75.7%, *p* < 0.001). ACB consumption lowered TC in Eastern status (WMD = −2.29 mg/dL; 95%CI: −4.29 to −0.30; *p* = 0.024; *I*^2^ = 68.9%, *p* < 0.001).

When trials utilized less than 300 mg of ACB (*I*^2^ = 0.0%, *p* = 0.497) in prediabetes patients (*I*^2^ = 7.4%, *p* = 0.299), between-study heterogeneity was eliminated.

### Effect of ACB intake on LDL (mg/dL) and subgroup analysis

In total, 43 effect sizes from 43 trials (*n* total = 5,358, *n* IG = 2,692, *n* CG = 2,666) were considered in this analysis. Overall, we observed no difference in LDL (mg/dL) reduction between the intervention and control groups (WMD = 0.77 mg/dL; 95%CI: −1.19, 2.73; *p* = 0.440; *I*^2^ = 81.3%, *p* < 0.001) (Figure 2C). Subgroup analyses conducted have shown that ACB treatment had an increased effect on LDL with an intervention dose of <300 mg/day (WMD = 3.79 mg/dL; 95%CI: 0.76 to 6.83; *p* = 0.014; *I*^2^ = 40.0%, *p* = 0.067). Between-study heterogeneity was eliminated in studies with prediabetes participants (*I^2^* = 61.8%, *p* = 0.106) and dose of <300 mg/day (*I*^2^ = 40.0%, *p* = 0.067) (Table 3).

### Effect of ACB intake on HDL (mg/dL) and subgroup analysis

In total, 53 effect sizes from 53 trials (*n* total = 5,670, *n* IG = 2,845, *n* CG = 2,825) were considered in this analysis. Changes in HDL (mg/dL) were assessed. The variations in HDL (mg/dL) when compared with controls were not significant (WMD = 0.21 mg/dL; 95%CI: −0.66, 1.10; *p* = 0.629; *I*^2^ = 86.8%, *p* < 0.001) (Figure 2D). Subgroup analyses conducted have shown that ACB treatment had a reduction effect on HDL with an intervention dose of <300 mg/day (WMD = −1.36 mg/dL; 95%CI: −2.49 to −0.24; *p* = 0.017; *I*^2^ = 42.7%, *p* = 0.040). Between-study heterogeneity was eliminated in studies with baseline HDL of <40 mg/dL (*I*^2^ = 0.0%, *p* = 0.991) and prediabetes patients (*I*^2^ = 0.0%, *p* = 0.681) (Table 3).

### Effect of ACB intake on FBG (mg/dL) and subgroup analysis

**Figure 3:**
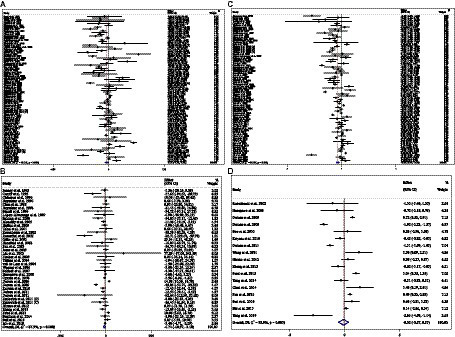
Forest plot detailing weighted mean difference and 95% confidence intervals (CIs) for the effect of acarbose consumption on **(A)** FBG (mg/dL); **(B)** insulin (pmol/L); **(C)** HbA1c (%); and **(D)** HOMA-IR.

Combining 81 effect sizes from 78 studies [*n* total = 10,008, *n* intervention group (IG) = 4,788, *n* control group (CG) = 5,220] has shown that ACB treatment had a significant effect on FBG (mg/dL) in an intervention group, compared with a placebo group (WMD = −3.55 mg/dL; 95%CI: −6.29 to −0.81; *p* = 0.01; *I*^2^ = 93.1%, *p* < 0.001) (Figure 3A). Subgroup analyses conducted have shown that ACB treatment had a reduction effect on FBG (mg/dL) in any baseline FBG (<100 mg/dL and ≥ 100 mg/dL) [(WMD = −8.78 mg/dL; 95%CI: −15.98 to −1.58; *p* = 0.017; *I*^2^ = 87.7%, *p* < 0.001) and (WMD = −3.41 mg/dL; 95%CI: −6.34 to −0.48; *p* = 0.022; *I*^2^ = 93.3%, *p* < 0.001), respectively]; ACB treatment had a reduction effect on FBG (mg/dL) in a trial duration of ≥24 weeks (WMD = −3.99 mg/dL; 95%CI: −7.89 to −0.09; *p* = 0.045; *I*^2^ = 95.0%, *p* = <0.001); ACB treatment had a reduction effect on FBG (mg/dL) with an intervention dose of ≥300 mg/day (WMD = −4.82 mg/dL; 95%CI: −8.06 to −1.58; *p* = 0.004; *I*^2^ = 94.0%, *p* < 0.001); ACB intake had a reduction effect on FBG (mg/dL) in prediabetes (WMD = −8.78 mg/dL; 95%CI: −15.98 to −1.58; *p* = 0.017; *I*^2^ = 87.7%, *p* < 0.001) and T2D patients (WMD = −3.41 mg/dL; 95%CI: −6.34 to −0.48; *p* = 0.022; *I*^2^ = 93.3%, *p* < 0.001); and ACB intake had a reduction effect on FBG (mg/dL) in Western status (WMD = −5.54 mg/dL; 95%CI: −10.36 to −0.71; *p* = 0.024; *I*^2^ = 81.0%, *p* < 0.001). Subgroup analyses indicated a significant between-study heterogeneity in all subgroups (Table 3).

### Effect of ACB intake on serum insulin (pmol/L) and subgroup analysis

Combining 39 effect sizes from 37 studies (*n* total = 3,561, *n* IG = 1,775, *n* CG = 1,786) has shown that ACB treatment had a significant effect on serum insulin (pmol/L) in an intervention group, compared with a placebo group (WMD = −6.73 pmoL/L; 95%CI: −1.37 to −3.10; *p* < 0.001; *I*^2^ = 87.3%, *p* < 0.001) (Figure 3B). Subgroup analyses conducted have shown that ACB treatment had a reduction effect on serum insulin (pmol/L) in the trial duration of <24 weeks (WMD = −10.42 pmoL/L; 95%CI: −18.29 to −2.55; *p* = 0.009; *I*^2^ = 85%, *p* < 0.001); ACB treatment had a reduction effect on serum insulin (pmol/L) in any trial dose (<300 mg/day and ≥ 300 mg/day) [(WMD = −7.78 pmoL/L; 95%CI: −13.66 to −1.90; *p* = 0.009; *I*^2^ = 8.0%, *p* < 0.001) and (WMD = −6.56 pmoL/L; 95%CI: −10.59 to −2.53; *p* = 0.001; *I*^2^ = 89.3%, *p* < 0.001), respectively]; ACB intake had a reduction effect on insulin (pmol/L) in T2D patients (WMD = −7.03 pmoL/L; 95%CI: −10.48 to −3.21; *p* < 0.001; *I*^2^ = 89.6%, *p* < 0.001); and ACB intake had a reduction effect on insulin in Eastern status (WMD = −5.26 pmoL/L; 95%CI: −9.50 to −1.02; *p* = 0.015; *I*^2^ = 91.9%, *p* < 0.001) and Western status (WMD = −11.65 pmoL/L; 95%CI: −20.38 to −2.93; *p* = 0.009; *I*^2^ = 63.4%, *p* < 0.001). Subgroup analyses indicated a significant between-study heterogeneity in all subgroups except in patients with prediabetes (*I*^2^ = 0.0%, *p* = 0.500) (Table 3).

### Effect of ACB intake on serum HbA1c (%) and subgroup analysis

Combining 77 effect sizes from 74 studies (*n* total = 10,459, *n* IG = 4,904, *n* CG = 5,555) has shown that ACB treatment had a significant effect on serum HbA1c (%) in an intervention group, compared with a placebo group (WMD = −0.32%; 95%CI: −0.45 to −0.20; *p* < 0.001; *I*^2^ = 96.3%, *p* < 0.001) (Figure 3C). Subgroup analyses conducted have shown that ACB treatment had a reduction effect on HbA1c (%) in any trial duration (<24 weeks and ≥ 24 weeks) [(WMD = −0.27%; 95%CI: −0.49 to −0.05; *p* = 0.018; *I*^2^ = 94.2%, *p* < 0.001) and (WMD = −0.37%; 95%CI: −0.53 to −0.21; *p* < 0.001; *I*^2^ = 97.0%, *p* < 0.001), respectively]; ACB treatment had a reduction effect on HbA1c (%) with an intervention dose of ≥300 mg/day (WMD = −0.39%; 95%CI: −0.54 to −0.23; *p* < 0.001; *I*^2^ = 96.9%, *p* < 0.001); ACB intake had a reduction effect on HbA1c (%) in T2D patients (WMD = −0.29%; 95%CI: −0.43 to −0.16; *p* < 0.001; *I*^2^ = 96.3%, *p* < 0.001); and ACB intake had a reduction effect on HbA1c in Eastern status (WMD = −0.28%; 95%CI: −0.43 to −0.13; *p* < 0.001; *I*^2^ = 97.1%, *p* < 0.001) and Western status (WMD = −0.41%; 95%CI: −0.61 to −0.21; *p* < 0.001; *I*^2^ = 89.6%, *p* < 0.001). Subgroup analyses indicated a significant between-study heterogeneity in all subgroups except in prediabetic patients (*I*^2^ = 0.0%, *p* = 0.709) (Table 3).

### Effect of ACB intake on HOMA-IR and subgroup analysis

Combining 17 effect sizes from 17 studies (*n* total = 2,852, *n* IG = 1,443, *n* CG = 1,409) has shown that ACB treatment had no significant effect on serum HOMA-IR in an intervention group, compared with a placebo group (WMD = −0.10; 95%CI: −0.57 to −0.36; *p* = 0.670; *I*^2^ = 95.9%, *p* < 0.001) (Figure 3D). ACB intake had a reduction effect on HOMA-IR in Eastern status (WMD = −1.28; 95%CI: −2.50 to −0.06; *p* = 0.040; *I*^2^ = 88.2%, *p* < 0.001). Subgroup analyses indicated a significant between-study heterogeneity in all subgroups (Table 3).

### Effect of ACB intake on SBP (mmHg) and subgroup analysis

**Figure 4:**
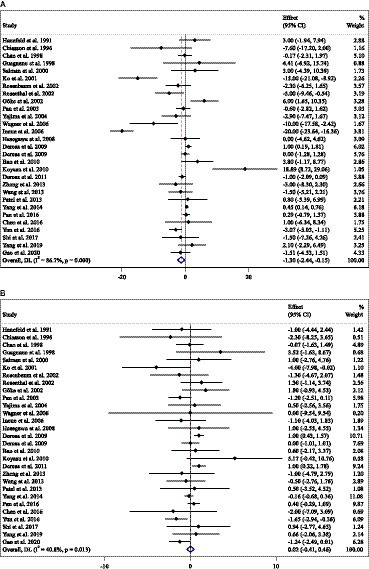
Forest plot detailing weighted mean difference and 95% confidence intervals (CIs) for the effect of acarbose consumption on **(A)** SBP (mmHg) and **(B)** DBP (mmHg).

In total, 29 effect sizes from 29 trials (*n* total = 4,046, *n* IG = 2,031, *n* CG = 2,015) were considered in this analysis. Changes in SBP (mmHg) were assessed. The variations in SBP (mmHg), when compared with controls, were significant (WMD = −1.29 mmHg; 95%CI: −2.44, −0.15; *p* = 0.027; *I*^2^ = 86.7%, *p* < 0.001) (Figure 4A). Subgroup analyses conducted have shown that ACB treatment had an increased effect on SBP in any baseline SBP (<130 and ≥ 130 mmHg) [(WMD = 0.40 mmHg; 95%CI: 0.11 to 0.69; *p* = 0.006; *I*^2^ = 0.0%, *p* = 0.476) and (WMD = −2.49 mmHg; 95%CI: −4.48 to −0.50; *p* = 0.014; *I*^2^ = 90.4%, *p* < 0.001), respectively] (Table 3). ACB significantly impacted on SBP with an intervention dose of ≥300 mg/day (WMD = −1.57 mmHg; 95%CI: −2.88 to −0.26; *p* = 0.019; *I*^2^ = 88.9%, *p* < 0.001). Between-study heterogeneity was eliminated in studies with baseline SBP of <130 mmHg (*I*^2^ = 0.0%, *p* = 0.476) (Table 3).

### Effect of ACB intake on DBP (mmHg) and subgroup analysis

In total, 29 effect sizes from 29 trials (*n* total = 4,046, *n* IG = 2,031, *n* CG = 2,015) were considered in this analysis. Changes in DBP (mmHg) were assessed. The variations in DBP (mmHg) when compared with controls were not significant (WMD = 0.02 mmHg; 95%CI: −0.41, 0.45; *p* = 0.925; *I*^2^ = 40.8%, *p* = 0.013) (Figure 4B). Subgroup analyses conducted have shown that ACB treatment had a significant effect on DBP in any intervention dose (<300 and ≥ 300 mg) [(WMD = −1.13 mmHg; 95%CI: −1.86 to −0.41; *p* = 0.002; *I*^2^ = 4.2%, *p* = 0.397) and (WMD = 0.38 mmHg; 95%CI: 0.04 to 0.73; *p* = 0.028; *I*^2^ = 13.8%, *p* = 0.280), respectively] (Table 3). Between-study heterogeneity was eliminated in studies with baseline DBP of <80 mmHg (*I*^2^ = 0.0%, *p* = 0.520), duration of <24 weeks (*I*^2^ = 0.0%, *p* = 0.720), and any intervention dose (<300 and ≥ 300 mg) [(*I*^2^ = 4.2%, *p* = 0.397) and (*I*^2^ = 13.8%, *p* = 0.280), respectively] (Table 3).

### Effect of ACB intake on CRP (mg/L) and subgroup analysis

**Figure 5:**
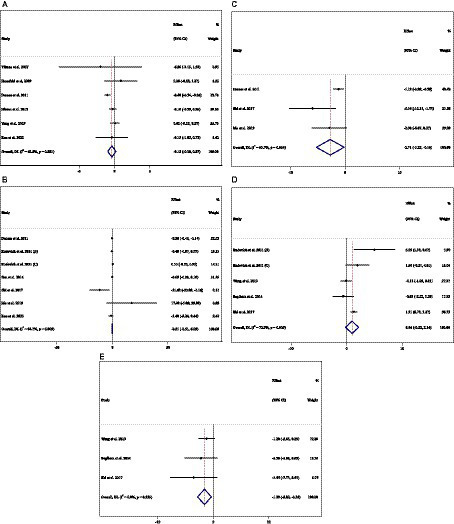
Forest plot detailing weighted mean difference and 95% confidence intervals (CIs) for the effect of acarbose consumption on **(A)** CRP (mg/L); **(B)** IL-6 (pg/mL); **(C)** TNF-α (pg/mL); **(D)** adiponectin (ng/mL); and **(E)** leptin (ng/mL).

Combining 6 effect sizes from 6 studies (*n* total = 572, *n* IG = 289, *n* CG = 283) has shown that ACB treatment had no significant effect on CRP (WMD = −0.15 mg/dL; 95%CI: −0.37, 0.07; *p* = 0.185; *I*^2^ = 62.5%, *p* = 0.021) (Figure 5A). Subgroup analyses conducted have shown that ACB treatment had no significant effect in all subgroups (Table 3). Between-study heterogeneity disappeared in studies with a duration of <24 weeks (*I*^2^ = 0.0%, *p* = 0.435) (Table 3).

### Effect of ACB intake on IL-6 (pg/mL) and subgroup analysis

Combining 7 effect sizes from 5 studies (*n* total = 594, *n* IG = 302, *n* CG = 292) has shown that ACB treatment had no significant effect on IL-6, according to the findings (WMD = −0.20 pg./mL; 95%CI: −0.50, 0.09; *p* = 0.179; *I*^2^ = 64.7%, *p* = 0.009) (Figure 5B). After subgroup analysis, heterogeneity disappeared in studies that used ACB with a duration of ≥24 weeks (*I*^2^ = 61.2%, *p* = 0.076) and normal BMI (*I*^2^ = 62.5%, *p* = 0.102) (Table 3).

### Effect of ACB intake on TNF-α (pg/mL) and subgroup analysis

Overall, 3 effect sizes from 3 clinical trials (*n* total = 294, *n* IG = 148, *n* CG = 146) in the overall population were included in this analysis. Pooled effect sizes indicated that there was a significant decrease in TNF-α (WMD = −2.70 pg./mL; 95%CI: −5.25, −0.16; *p* = 0.037; *I*^2^ = 63.7%, *p* = 0.064) (Figure 5C) after ACB consumption (Table 3). There was no significant association between subgroups and mean changes in TNF-α.

### Effect of ACB intake on adiponectin (ng/mL) and subgroup analysis

Five effect sizes from three clinical trials (*n* total = 241, *n* IG = 119, *n* CG = 122) were included in this meta-analysis. The results indicated that there was no significant effect in adiponectin (WMD = 0.95 ng/mL; 95%CI: −0.22, 2.13; *p* = 0.112; *I*^2^ = 72.9%, *p* = 0.005) (Figure 5D) after ACB consumption (Table 3). There was no significant association between subgroups and mean changes in adiponectin.

### Effect of ACB intake on leptin (ng/mL) and subgroup analysis

Overall, three effect sizes from three clinical trials (*n* total = 137, *n* IG = 67, *n* CG = 70) were included in this meta-analysis. The results indicated that there was a significant reduction effect leptin (WMD = −1.58 ng/mL; 95%CI: −2.82, −0.35; *p* = 0.012; *I*^2^ = 0.0%, *p* = 0.523) (Figure 5E) after ACB consumption (Table 3). There was no significant association between subgroups and the mean of leptin.

### Effect of ACB intake on body weight (kg) and subgroup analysis

**Figure 6:**
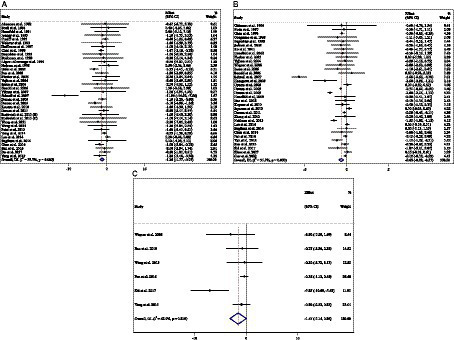
Forest plot detailing weighted mean difference and 95% confidence intervals (CIs) for the effect of acarbose consumption on **(A)** weight (kg); **(B)** BMI (kg/m^2^); and **(C)** WC (cm).

Combining 36 effect sizes from 34 studies (*n* total = 6,232, *n* IG = 3,122, *n* CG = 3,110) has shown that ACB treatment had a significant effect on body weight in an intervention group, compared with a placebo group (WMD = −1.25 kg; 95%CI: −1.79 to −0.75; *p* < 0.001; *I*^2^ = 56.7%, *p* < 0.001) (Figure 6A). Subgroup analyses conducted have shown that ACB treatment had a reduction effect on body weight in any trial duration (<24 weeks and ≥ 24 weeks) [(WMD = −1.93 kg; 95%CI: −3.31 to −0.54; *p* = 0.006; *I*^2^ = 54.6%, *p* = 0.005) and (WMD = −1.01 kg; 95%CI: −1.50 to −0.53; *p* < 0.001; *I*^2^ = 54.0%, *p* = 0.002), respectively]; ACB treatment had a reduction effect on body weight in any trial dose (<300 mg/day and ≥ 300 mg/day) [(WMD = −1.58 kg; 95%CI: −2.43 to −0.73; *p* < 0.001; *I*^2^ = 0.0%, *p* = 0.847) and (WMD = −1.24 kg; 95%CI: −1.82 to −0.66; *p* < 0.001; *I*^2^ = 62.4%, *p* < 0.001), respectively]; ACB intake had a reduction effect on body weight in T2D patients (WMD = −1.18 kg; 95%CI: −1.72 to −0.65; *p* < 0.001; *I*^2^ = 57.5%, *p* < 0.001); and ACB intake had a reduction effect on weight in Eastern status (WMD = −1.01 kg; 95%CI: −1.39 to −0.62; *p* < 0.001; *I*^2^ = 20.6%, *p* = 0.186) and Western status (WMD = −1.71 kg; 95%CI: −3.23 to −0.19; *p* = 0.027; *I*^2^ = 76.2%, *p* < 0.001) (Table 3). Subgroup analyses indicated no significant between-study heterogeneity in studies conducted in the intervention dose of <300 mg/day (*I*^2^ = 0.0%, *p* = 0.847), Eastern status (*I*^2^ = 20.6%, *p* = 0.186), and prediabetes patients (*I*^2^ = 0.0%, *p* = 0.986), which were the probable sources of heterogeneity (Table 3).

### Effect of ACB intake on BMI and subgroup analysis

Combining 34 effect sizes from 34 studies (*n* total = 3,377, *n* IG = 1,692, *n* CG = 1,685) has shown that ACB treatment had a significant effect on BMI in an intervention group, compared with a placebo group (WMD = −0.64 kg/m^2^; 95%CI: −0.92 to −0.37; *p* < 0.001; *I*^2^ = 91.9%, *p* < 0.001) (Figure 6B). Subgroup analyses conducted have shown that ACB treatment had a reduction effect on BMI in any trial duration (<24 weeks and ≥ 24 weeks), [(WMD = −0.96 kg/m^2^; 95%CI: −1.56 to −0.37; *p* = 0.001; *I*^2^ = 94.1%, *p* < 0.001) and (WMD = −0.30 kg/m^2^; 95%CI: −0.52 to −0.07; *p* = 0.009; *I*^2^ = 81.1%, *p* < 0.001), respectively]; ACB treatment had a reduction effect on BMI in trial dose of ≥300 mg/day (WMD = −0.77 kg/m^2^; 95%CI: −1.11 to −0.43; *p* < 0.001; *I*^2^ = 93.2%, *p* < 0.001); ACB intake had a reduction effect on BMI in T2D patients (WMD = −0.65 kg/m^2^; 95%CI: −0.95 to −0.36; *p* < 0.001; *I*^2^ = 92.4%, *p* < 0.001); and ACB intake had a reduction effect on BMI in Eastern status (WMD = −0.50 kg/m^2^; 95%CI: −0.75 to −0.24; *p* < 0.001; *I^2^* = 90.6%, *p* < 0.001). Subgroup analyses indicated a significant between-study heterogeneity in all subgroups, except in prediabetes patients (*I^2^* = 0.0%, *p* = 0.945) (Table 3).

### Effect of ACB intake on WC and subgroup analysis

Combining 6 effect sizes from 6 studies (*n* total = 1,063, *n* IG = 531, *n* CG = 532) has shown that ACB treatment had no significant effect on WC in an intervention group compared with a placebo group (WMD = −1.55 cm; 95%CI: −3.14 to 0.04; *p* = 0.056; *I^2^* = 62.9%, *p* = 0.019) (Figure 6C). Subgroup analyses conducted have shown that ACB treatment had no significant effect in all subgroups (Table 3). Subgroup analyses indicated no significant between-study heterogeneity in studies conducted in a trial duration of ≥24 weeks (*I^2^* = 0.0%, *p* = 0.852) (Table 3).

### Effect of ACB intake on ALT (U/L) and subgroup analysis

**Figure 7:**
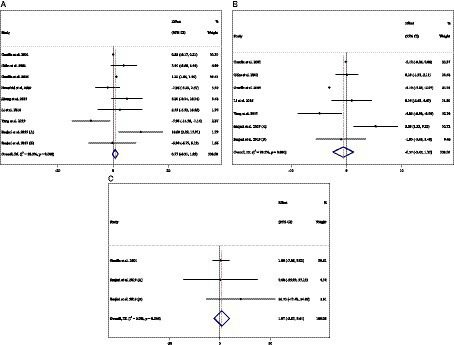
Forest plot detailing weighted mean difference and 95% confidence intervals (CIs) for the effect of acarbose consumption on **(A)** ALT (U/L); **(B)** AST (U/L); and **(C)** ALP (U/L).

Combining 9 effect sizes from 8 studies (*n* total = 905, *n* IG = 453, *n* CG = 452) has shown that ACB treatment had no significant effect on ALT (U/L) in an intervention group compared with a placebo group (WMD = 0.71 U/L; 95%CI: −0.31 to 1.85; *p* = 0.164; *I^2^* = 92.2%, *p* < 0.001) (Figure 7A). Subgroup analyses conducted have shown that ACB treatment had an increased effect on ALT (U/L) with an intervention dose of <300 mg/day (WMD = 4.53 U/L; 95%CI: 0.71 to 8.36; *p* = 0.020; *I^2^* = 11.0%, *p* = 0.338). Subgroup analyses indicated no significant between-study heterogeneity in studies conducted in trial duration of <24 weeks (*I^2^* = 54.2%, *p* = 0.068) and intervention dose of <300 mg/day (*I^2^* = 11.0%, *p* = 0.338), which were the probable sources of heterogeneity (Table 3).

### Effect of ACB intake on AST (U/L) and subgroup analysis

Combining 7 effect sizes from 6 studies (*n* total = 778, *n* IG = 391, *n* CG = 387) has shown that ACB treatment had no significant effect on AST (intervention group), compared with a placebo group (WMD = −0.57 U/L; 95%CI: −2.45 to 1.30; *p* = 0.550; *I^2^* = 99.3%, *p* < 0.001) (Figure 7B). Subgroup analyses conducted have shown that ACB treatment had no reduction effect on AST (U/L) in any subgroups. Subgroup analyses indicated no significant between-study heterogeneity in studies conducted in a trial duration of ≥24 weeks (*I^2^* = 49.4%, *p* = 0.115) and doses of <300 mg (*I^2^* = 57.6%, *p* = 0.095), which were the probable sources of heterogeneity (Table 3).

### Effect of ACB intake on ALP (U/L) and subgroup analysis

Combining 3 effect sizes from 2 studies (*n* total = 130, *n* IG = 67, *n* CG = 63) has shown that ACB treatment had no significant effect on ALP (U/L) in an intervention group, compared with a placebo group (WMD = 1.97 U/L; 95%CI: −5.67 to 9.61; *p* = 0.613; *I^2^* = 0.0%, *p* = 0.544) (Figure 7C) (Table 3). There was no significant association between subgroups and mean changes in ALP.

### Publication bias

Although the visual inspection of funnel plots showed slight asymmetries, no significant publication bias was detected for TC (mg/dL), LDL (mg/dL), HDL (mg/dL), FBG (mg/dL), insulin (pmol/L), HbA1c (%), HOMA-IR, SBP (mmHg), DBP (mmHg), CRP (mg/L), IL-6 (pg/mL), TNF-α, adiponectin, leptin, weight (kg), BMI (kg/m^2^), WC (cm), ALT (U/L), AST (U/L), and ALP (U/L). The p-value for Egger’s test including TG (mg/dL) (P_Egger’s test_ = 0.086, Supplementary Figure S1A), TC (mg/dL) (P_Egger’s test_ = 0.567, P _Begg’s test=_0.474, Supplementary Figure S1B), LDL (mg/dL) (P_Egger’s test=_ 0.448, P _Begg’s test_ = 0.477, Supplementary Figure S1C), HDL (mg/dL) (P_Egger’s test=_ 0.149, P_Begg’s test_ = 0.872, Supplementary Figure S1D), FBG (mg/dL) (P_Egger’s test_ = 0.258, P_Begg’s test=_0.835, Supplementary Figure S1E), insulin (pmol/L) (P_Egger’s test_ = 0.287, P_Begg’s test_ = 0.453, Supplementary Figure S1F), HOMA-IR (P_Egger’s test_ = 0.392, P_Begg’s test=_0.564, Supplementary Figure S1G), SBP (mmHg) (P_Egger’s test_ = 0.106, P_Begg’s test_ = 1.000, Supplementary Figure S1I), DBP (mmHg) (P_Egger’s test_ = 0.456, P _Begg’s test_ = 0.866, Supplementary Figure S1J), CRP (mg/L) (P_Egger’s test_ = 0.482, P _Begg’s test_ = 1.000, Supplementary Figure S1K), IL-6 (pg/mL) (P_Egger’s test_ = 0.707, P_Begg’s test_ = 1.000, Supplementary Figure S1L), TNF-α (P_Egger’s test_ = 0.194, P_Begg’s test_ = 0.296, Supplementary Figure S1M), adiponectin (P_Egger’s test_ = 0.885, P_Begg’s test_ = 0.462, Supplementary Figure S1N), leptin (P_Egger’s test_ = 0.070, P_Begg’s test_ = 0.296, Supplementary Figure S1O), weight (kg) (P_Egger’s test_ = 0.286, P_Begg’s test_ = 0.924, Supplementary Figure S1P), BMI (kg/m^2^) (P_Egger’s test_ = 0.740, P_Begg’s test_ = 0.116, Supplementary Figure S1Q), WC (cm) (P_Egger’s test_ = 0.179, P_Begg’s test_ = 0.260, Supplementary Figure S1R), ALT (U/L) (P_Egger’s test_ = 0.961, P_Begg’s test_ = 1.000, Supplementary Figure S1S), AST (U/L) (P_Egger’s test_ = 0.756, P_Begg’s test_ = 1.000, Supplementary Figure S1T), and ALP (U/L) (P_Egger’s test_ = 0.536, P_Begg’s test_ = 1.000, Supplementary Figure S1U). Although significant publication bias was detected for HbA1C with Egger’s test P_Egger’s test_ = 0.002 (Supplementary Figure S1H). Moreover significant publication bias was detected for TG with Begg’s test P_Begg’s test_ = 0.002 (Supplementary Figure S1A).

### Non-linear dose–response analysis

For the dose–response analysis between ACB treatment and TG (mg/dL), TC (mg/dL), LDL (mg/dL), HDL (mg/dL), FBG (mg/dL), insulin (pmol/L), HbA1c (%), HOMA-IR, SBP (mmHg), DBP (mmHg), CRP (mg/L), IL-6 (pg/mL), weight (kg), BMI (kg/m^2^), WC (cm), ALT (U/L), AST (U/L), and ALP (U/L), we used a one-stage non-linear dose–response analysis.

We did not find a significant non-linear relationship between dose (mg/day) (coefficients = −7.91, *p* = 0.457) and duration (weeks) (coefficients = 39.95, *p* = 0.399) of the intervention group and changes in TG (Supplementary Figures S2A, S3A). In addition, there was no significant non-linear relationship between dose (mg/day) (coefficients = −19.96, *p* = 0.116) and changes in TC. There was a significant non-linear relationship between the duration of the intervention (weeks) (coefficients = −18.20, *p* = 0.042) and changes in TC. ACB’s effective duration for reducing the TC was more than 50 weeks (Supplementary Figures S2B, S3B).

Furthermore, we did not find a significant non-linear relationship between dose (mg/day) (coefficients = 8.35, *p* = 0.232) and duration (weeks) (coefficients = 1.86, *p* = 0.118) of the intervention group, and changes in LDL (Supplementary Figures S2C, S3C) and HDL for dose (coefficients = 0.38, *p* = 0.189) and duration of the intervention (weeks) (coefficients = −0.08, *p* = 0.516) (Supplementary Figures S2D, S3D).

There was no significant non-linear association between dose (mg/day) (coefficients = 4.79, *p* = 0.571) and intervention duration (weeks) (coefficients = 10.35, *p* = 0.413) and changes in FBG (Supplementary Figures S2E, S3E) and insulin with dose (coefficients = 142.20, *p* = 0.290) and duration of the intervention (weeks) group (coefficients = 16.92, *p* = 0.830) (Supplementary Figures S2F, S3F). Furthermore, we did not find a significant non-linear relationship between dose (mg/day) (coefficients = −1.01, *p* = 0.583) and duration (weeks) (coefficients = 1.62, *p* = 0.525) of the intervention group and changes in HbA1C% (Supplementary Figures S2G, S3G). We did not find a significant non-linear relationship between dose (mg/day) (coefficients = 1.19, *p* = 0.188) and duration (weeks) (coefficients = −0.13, *p* = 0.131) of the intervention group and changes in HOMA-IR (Supplementary Figures S2H, S3H).

We did not find a significant non-linear relationship between dose (mg/day) (coefficients = −0.32, *p* = 0.946) and duration (weeks) (coefficients = 1.82, *p* = 0.690) of the intervention and changes in SBP (Supplementary Figures S2I, S3I). In addition, there was no significant non-linear relationship between dose (mg/day) (coefficients = −0.27, *p* = 0.908) and duration of the intervention (weeks) (coefficients = −3.54, *p* = 0.050) and changes in DBP (Supplementary Figures S2J, S3J).

In addition, we found a significant non-linear relationship between dose (mg/day) (coefficients = −12.69, *p* = 0.009) and changes in CRP, i.e., a dose of 180 mg/day has a prominent effect on the decrement of CRP. In addition, we did not find a significant non-linear relationship between the duration (weeks) (coefficients = 25.29, *p* = 0.266) of the intervention and changes in CRP (Supplementary Figures S2K, S3K). We did not find a significant non-linear relationship between dose (mg/day) (coefficients = −10.07, *p* = 0.738) and duration (weeks) (coefficients = 1.14, *p* = 0.327) of the intervention and changes in IL-6 (Supplementary Figures S2L, S3L).

Moreover, the current study indicates that there was no significant non-linear relationship between dose (mg/day) (coefficients = −0.14, *p* = 0.930) and duration (weeks) (coefficients = −2.31, *p* = 0.424) of the intervention and changes in weight (Supplementary Figures S2P, S3P), BMI changes with dose (mg/day) (coefficients = 0.83, *p* = 0.187) and duration (weeks) (coefficients = 0.56, *p* = 0.468) (Supplementary Figures S2Q, S3Q), and WC changes with dose (mg/day) (coefficients = 1.23, *p* = 0.742) and duration of the intervention (weeks) (coefficients = 0.59, *p* = 0.295) (Supplementary Figures S2R, S3R).

Moreover, liver enzymes did not find a significant non-linear relationship between dose (mg/day) (coefficients = 5.73, *p* = 0.129) and duration (weeks) (coefficients = 1.81, *p* = 0.127) of the intervention and changes in ALT (Supplementary Figures S2S, S3S), between dose (mg/day) (coefficients = 3.06, *p* = 0.290) and duration (weeks) (coefficients = −1.65, *p* = 0.368) of the intervention and changes in AST (Supplementary Figures S2T, S3T), and ALP changes with dose (mg/day) (coefficients = 15.13, *p* = 0.613) and duration of the intervention (weeks) (coefficients = −0.33, *p* = 0.613) (Supplementary Figures S2U, S3U).

### Meta-regression analysis

Meta-regression analyses were performed to assess whether TG (mg/dL), TC (mg/dL), LDL (mg/dL), HDL (mg/dL), FBG (mg/dL), insulin (pmol/L), HbA1c (%), HOMA-IR, SBP (mmHg), DBP (mmHg), CRP (mg/L), IL-6 (pg/mL), TNF-α, adiponectin, leptin, weight (kg), BMI (kg/m^2^), WC (cm), ALT (U/L), AST (U/L), and ALP (U/L) were affected by ACB doses and intervention durations.

We did not find a significant linear relationship between dose (mg/day) (coefficients = 0.07, *p* = 0.853) and duration (weeks) (coefficients = −0.30, *p* = 0.070) of the intervention group and changes in TG (Supplementary Figures S4A, S5A), TC changes with dose (mg/day) (coefficients = −2.14, *p* = 0.076) and duration (weeks) (coefficients = −0.29, *p* = 0.331) (Supplementary Figures S4B, S5B), and HDL changes with dose (mg/day) (coefficients = 5.01, *p* = 0.056) and duration (weeks) (coefficients = 0.11, *p* = 0.813) (Supplementary Figures S4D, S5D). Furthermore, there was a significant linear association between dose (mg/day) (coefficients = −2.71, *p* = 0.044) and changes in LDL but not with intervention duration (weeks) (coefficients = −0.22, *p* = 0.551) (Supplementary Figures S4C, S5C).

The present study indicated a significant linear relationship between dose (mg/day) (coefficients = −0.51, *p* = 0.298) and duration of the intervention (weeks) (coefficients = 0.001, *p* = 0.991) and changes in FBG (Supplementary Figures S4E, S5E). In addition, there was no significant linear relationship between dose (mg/day) (coefficients = −0.43, *p* = 0.396) and duration of the intervention (weeks) (coefficients = 0.09, *p* = 0.554) and changes in insulin (Supplementary Figures S4F, S5F), also between dose (mg/day) (coefficients = −10.49, *p* = 0.477) and duration (weeks) (coefficients = −0.80, *p* = 0.791) of the intervention in HbA1C% (Supplementary Figures S4G, S5G) and between dose (mg/day) (coefficients = −20.45, *p* = 0.223) and duration (weeks) (coefficients = 1.49, *p* = 0.671) of the intervention in HOMA-IR (Supplementary Figures S4H, S5H).

We did not find a significant linear relationship between dose (mg/day) (coefficients = −2.11, *p* = 0.301) and duration (weeks) (coefficients = 0.30, *p* = 0.642) of the intervention and changes in SBP (Supplementary Figures S4I, S5I) and DBP with dose (mg/day) (coefficients = 6.87, *p* = 0.382) and duration of the intervention (weeks) (coefficients = −1.25, *p* = 0.617) (Supplementary Figures S4J, S5J).

Also, inflammatory markers, such as CRP changes with dose (mg/day) (coefficients = −5.50, *p* = 0.947) and intervention duration (weeks) (coefficients = −7.15, *p* = 0.271) (Supplementary Figures S4K, S5K), IL-6 changes with dose (mg/day) (coefficients = 0.66, *p* = 0.834) and duration (weeks) (coefficients = 1.24, *p* = 0.072) (Supplementary Figures S4L, S5L), and TNF-α changes with dose (mg/day) (coefficients = −3.44, *p* = 1.000) and duration (weeks) (coefficients = 3.73, *p* = 0.711) (Supplementary Figures S4M, S5M), have not shown any significant association.

Moreover, for adipokines, we did not find a significant linear relationship between dose (mg/day) (coefficients = 0, *p* = 1.000) and duration of the intervention (weeks) (coefficients = −1.01, *p* = 0.499) and changes in adiponectin (Supplementary Figures S4N, S5N) and leptin with dose (mg/day) (coefficients = −1.70, *p* = 1.000) and duration (weeks) (coefficients = 4.93, *p* = 0.383) (Supplementary Figures S4O, S5O).

In the present study, we found that there was no significant linear association between dose (mg/day) (coefficients = −1.10, *p* = 0.854) and duration (weeks) (coefficients = 1.72, *p* = 0.445) of the intervention and changes in weight (Supplementary Figures S4P, S5P), BMI changes with dose (mg/day) (coefficients = −13.90, *p* = 0.218) and duration (weeks) (coefficients = 0.37, *p* = 0.914) (Supplementary Figures S4Q, S5Q), and WC changes with dose (mg/day) (coefficients = −7.52, *p* = 0.643) and duration (weeks) (coefficients = 2.62, *p* = 0.348) (Supplementary Figures S4R, S5R).

There was no significant linear relationship between dose (mg/day) (coefficients = −10.59, *p* = 0.116) and duration (weeks) (coefficients = −0.99, *p* = 0.229) of the intervention and changes in ALT (Supplementary Figures S4S, S5S), AST changes with dose (mg/day) (coefficients = −19.57, *p* = 0.127) and duration (weeks) (coefficients = −1.04, *p* = 0.534) of the intervention (Supplementary Figures S4T, S5T), and ALP changes with dose (mg/day) (coefficients = −4.91, *p* = 0.676) and duration (weeks) (coefficients = −0.94, *p* = 0.586) of the intervention (Supplementary Figures S4U, S5U).

### Sensitivity analysis

According to the sensitivity analysis, no study affected the overall results of TG (mg/dL), LDL (mg/dL), HDL (mg/dL), FBG (mg/dL), insulin (pmol/L), HbA1c (%), HOMA-IR, DBP (mmHg), CRP (mg/L), IL-6 (pg/mL), adiponectin (ng/mL), weight (kg), BMI (kg/m^2^), WC (cm), ALT (U/L), AST (U/L), and ALP (U/L) after removing individual study effects. Although Inoue et al. (137) (WMD = −1.67; 95%CI: −3.47, 0.11) affected the overall results of TC, Ko et al. (70) (WMD = −0.97; 95%CI: −2.08, 0.13) and Yang et al. (21) (WMD = −1.41; 95%CI: −2.86, 0.03) affected the overall results of SBP, Mo et al. (127) (WMD = −3.09; 95%CI: −7.67, 1.47) affected the overall results of TNF-a, and Sugihara et al. (110) (WMD = −1.52; 95%CI: −3.11, 0.06) affected the overall results of leptin.

### GRADE assessment

We used the GRADE evidence profile and the certainty in outcomes of ACB treatment on TG (mg/dL), TC (mg/dL), LDL (mg/dL), HDL (mg/dL), FBG (mg/dL), insulin (pmol/L), HbA1c (%), HOMA-IR, SBP (mmHg), DBP (mmHg), CRP (mg/L), IL-6 (pg/mL), TNF-α (pg/mL), adiponectin (ng/mL), leptin (ng/mL), weight (kg), BMI (kg/m^2^), WC (cm), ALT (U/L), AST (U/L), and ALP (U/L), which were shown in Table 4. The quality of evidence was moderate due to the risk of bias, inconsistency, imprecision for TG, LDL, HDL, HOMA-IR, CRP, IL-6, adiponectin, WC, ALT, and AST, and publication bias for TG. Also, the quality of evidence was low due to the risk of bias and inconsistency for TC, FBG, insulin, HbA1C, SBP, DBP, TNF-α, weight, BMI, and ALP, and imprecision for DBP and ALP. In addition, the quality of evidence was very low due to the risk of bias for leptin.

**Table 4:** GRADE profile of acarbose for cardiovascular risk factors in patients with T2D and impaired glucose tolerance.

Outcomes	Risk of bias	Inconsistency	Indirectness	Imprecision	Publication bias	WMD (95%CI)	Quality of evidence
TG	Serious limitation	Very serious limitation^a^	No serious limitation	No serious limitation	Serious limitation	−13.89 (−20.69, −7.09)	⊕ ⊕ ⊕◯ Moderate
TC	Serious limitation	Serious limitation^a^	No serious limitation	No serious limitation	No serious limitation	−2.26 (−4.18, −0.34)	⊕ ⊕ ◯◯ Low
LDL	Serious limitation	Very serious limitation^a^	No serious limitation	Serious limitation^b^	No serious limitation	0.77 (−1.19, 2.73)	⊕ ⊕ ⊕◯ Moderate
HDL	Serious limitation	Very serious limitation^a^	No serious limitation	Serious limitation^b^	No serious limitation	0.21 (−0.66, 1.10)	⊕ ⊕ ⊕◯ Moderate
FBG	Serious limitation	Very serious limitation^a^	No serious limitation	No serious limitation	No serious limitation	−3.55 (−6.29, −0.81)	⊕ ⊕ ◯◯ Low
Insulin	Serious limitation	Very serious limitation^a^	No serious limitation	No serious limitation	No serious limitation	−6.73 (−10.37, −3.10)	⊕ ⊕ ◯◯ Low
HbA1C	Serious limitation	Very serious limitation^a^	No serious limitation	No serious limitation	No serious limitation	−0.32 (−0.45, −0.20)	⊕ ⊕ ◯◯ Low
HOMA-IR	Serious limitation	Very serious limitation^a^	No serious limitation	Serious limitation^b^	No serious limitation	−0.10 (−0.57, 0.36)	⊕ ⊕ ⊕◯ Moderate
SBP	Serious limitation	Very serious limitation^a^	No serious limitation	No serious limitation	No serious limitation	−1.29 (−2.44, −0.15)	⊕ ⊕ ◯◯ Low
DBP	Serious limitation	No serious limitation	No serious limitation	Serious limitation^b^	No serious limitation	0.02 (−0.41, 0.45)	⊕ ⊕ ◯◯ Low
CRP	Serious limitation	Serious limitation^a^	No serious limitation	Serious limitation^b^	No serious limitation	−0.15 (−0.37, 0.07)	⊕ ⊕ ⊕◯ Moderate
IL-6	Serious limitation	Serious limitation^a^	No serious limitation	Serious limitation^b^	No serious limitation	−0.20 (−0.50, 0.09)	⊕ ⊕ ⊕◯ Moderate
TNF-α	Serious limitation	Serious limitation^a^	No serious limitation	No serious limitation	No serious limitation	−2.70 (−5.25, −0.16)	⊕ ⊕ ◯◯ Low
Adiponectin	Serious limitation	Serious limitation^a^	No serious limitation	Serious limitation^b^	No serious limitation	0.95 (−0.22, 2.13)	⊕ ⊕ ⊕◯ Moderate
Leptin	Serious limitation	No serious limitation	No serious limitation	No serious limitation	No serious limitation	−1.58 (−2.82, −0.35)	⊕◯◯◯ Very low
Weight	Serious limitation	Serious limitation^a^	No serious limitation	No serious limitation	No serious limitation	−1.25 (−1.79, −0.75)	⊕ ⊕ ◯◯ Low
BMI	Serious limitation	Very serious limitation^a^	No serious limitation	No serious limitation	No serious limitation	−0.64 (−0.92, −0.37)	⊕ ⊕ ◯◯ Low
WC	Serious limitation	Serious limitation^a^	No serious limitation	Serious limitation^b^	No serious limitation	−1.55 (−3.14, 0.04)	⊕ ⊕ ⊕◯ Moderate
ALT	Serious limitation	Very serious limitation^a^	No serious limitation	Serious limitation^b^	No serious limitation	0.76 (−0.31, 1.85)	⊕ ⊕ ⊕◯ Moderate
AST	Serious limitation	Very serious limitation^a^	No serious limitation	Serious limitation^b^	No serious limitation	−0.57 (−2.45, 1.30)	⊕ ⊕ ⊕◯ Moderate
ALP	Serious limitation	No serious limitation	No serious limitation	Serious limitation^b^	No serious limitation	1.97 (−5.67, 9.61)	⊕ ⊕ ◯◯ Low

a There is significant heterogeneity for TG (*I*^2^ = 86.0%), TC (*I*^2^ = 68.0%), LDL (*I*^2^ = 81.3%), HDL (*I*^2^ = 86.8%), FBG (*I*^2^ = 93.1%), insulin (*I*^2^ = 87.3%), HbA1C (*I*^2^ = 96.3%), HOMA-IR (*I*^2^ = 95.9%), SBP (*I*^2^ = 86.7%), CRP (*I*^2^ = 62.5%), IL-6 (*I*^2^ = 64.7%), TNF-α (*I*^2^ = 63.7%), adiponectin (*I*^2^ = 72.9%), weight (*I*^2^ = 56.7%), BMI (*I*^2^ = 91.9%), WC (*I*^2^ = 62.9%), ALT (*I*^2^ = 92.2%), and AST (*I*^2^ = 99.3%).

b There is no evidence of significant effects of acarbose consumption on LDL, HDL, HOMA_IR, DBP, CRP, IL-6, adiponectin, WC, ALT, AST, and ALP.

## Discussion

The present systematic review and meta-analysis investigated the effectiveness of the antidiabetic drug ACB on lipid profile, glycemic indexes, inflammatory factors, BP, and anthropometric indices among individuals with T2D, T1D, and IGT. The results showed that ACB significantly lowered HbA1c, FPG, serum insulin, BMI, body weight, leptin, SBP, TC, TG, and TNF-α but there was no significant effect between ACB intake and HOMA index, adiponectin, ALP, ALT, AST, CRP, DBP, HDL, LDL, IL-6, and WC in individuals with T2D, T1D, and IGT. Meta-regression analysis did not reveal any significant association between duration and dosage of ACB and HbA1c, FPG, serum insulin, BMI, leptin, SBP, TC, TG, TNF-α, and body weight. The findings from a non-linear dose–response analysis have indicated that the duration of ACB treatment needed to observe a significant reduction in TC levels is more than 50 weeks. Additionally, it has been observed that a daily ACB intake of 180 mg has a prominent effect on lowering CRP levels, which is a marker of inflammation. These results suggest that a longer treatment duration and a specific dosage of ACB can have notable impacts on TC and CRP levels, respectively, highlighting their potential in managing cardiovascular risk factors.

This current meta-analysis demonstrates that intake of ACB reduces HbA1c, FPG, and serum insulin by 33%, 3.56 pmol/L, and 6.74 mIU/mL, in patients with T2D, T1D, and IGT populations, respectively. In relation to HbA1c, a change of at least 0.5% is considered both statistically and clinically significant (138). Furthermore, a previous meta-analysis conducted by Hanefeld et al. examined the results of seven long-term randomized, double-blind, placebo-controlled trials involving patients with T2D. The findings of this analysis demonstrated that treatment with ACB was effective in improving glycemic control during the course of treatment (139). In a study by Wu et al., among 272 patients with T2D, 80 patients who consumed 150 mg/day of ACB for 16 weeks showed a decrease in HbA1c% level by 2% compared with the initial level, which was in line with our result (120). In the recent meta-analysis conducted by Yu et al. (24), the overall results from three studies involving 143 non-diabetic overweight or obese individuals (with a BMI of 25 kg/m^2^) did not show a significant reduction in FPG levels in the ACB group compared with the control group. These findings suggest that ACB treatment may not have a substantial impact on FPG levels in non-diabetic individuals with overweight or obesity (136).

Elevated blood sugar levels can result in disturbances in both the endothelium of blood vessels and the β-cells of the islets of Langerhans. This occurs due to the generation of oxidative stress and the release of inflammatory factors (140, 141). Among the mechanisms that can be mentioned for the harmful effects of continuous hyperglycemia, protein kinase C activation (PKC), oxidative phosphorylation, sorbitol formation, and glucose autooxidation are included (141). Activation of PKC can cause disorders such as microvascular disease in diabetic patients through the enhancement of factors such as thermoelectric generator 1 (TEG1), nuclear factor kappa B (NF-kB), and endothelin 1 (142). The consumption of acarbose (ACB) acts as a competitive inhibitor of intestinal alpha-glucosidases, including glucoamylase, sucrase, and pancreatic alpha-amylase. This mechanism leads to a delay in the absorption of glucose in the intestines, resulting in a reduction in blood sugar levels. Additionally, ACB may play a role in glucose metabolism by influencing the mitogen-activated protein kinase (MAPK) pathway. This pathway is involved in various cellular processes, including glucose regulation (143, 144). Moreover, the anti-inflammatory efficacy of ACB in the long term can reduce IL-6 and TNF-α compared with the baseline levels (127). In the hyperglycemic state, inflammation is stimulated by the activation of toll-like receptors (TLRs), as a result of which the level of IL-10 decreases, but the levels of pro-inflammatory cytokines, such as IL-6, TNF-α, and interferon γ, (IFN-γ) increases (145). Cytokines can suppress signals of insulin through the activation of kinase receptors, the arousal of NF-kB and the failure of pancreatic β-cells, and the process of apoptosis (145). In liver and muscle cells, TNF-α interferes with the action of insulin by binding to its receptors, and on the other hand, TNF-α reduces insulin-dependent glucose transporters such as glucose transporter-4 (GLUT-4) in the cell membrane, thereby reducing glucose absorption (146). IL-6 is effective in the homeostasis of glucose metabolism by inhibiting insulin secretion (147). Based on the reported cases, ACB has demonstrated potential effectiveness in controlling blood sugar levels and improving insulin sensitivity by reducing the levels of specific inflammatory factors. Another potential indirect mechanism of action could be attributed to the influence of short-chain fatty acids (SCFAs). SCFAs can enhance glucose absorption through the activation of receptors such as free fatty acid receptor 2 (FFAR2) and free fatty acid receptor 3 (FFAR3). These SCFAs can impact various factors involved in glucose homeostasis, including the activation of protein kinase activated by adenosine monophosphate (AMP), the release of the hormone glucagon-like peptide 1 (GLP-1), and the release of peptide YY (PYY). These mechanisms collectively contribute to the improvement of glucose regulation and overall glucose homeostasis (148, 149). PYY plays a role in the clearance of glucose in organs such as adipose tissue and muscle. It aids in regulating blood glucose levels. On the other hand, GLP-1 hormone is responsible for increasing insulin secretion and decreasing glucagon release (150).

Based on the results of the present study, the consumption of ACB effectively reduces the level of TNF-α and leptin by 2.71 pg./mL and 1.59 ng/mL, respectively. The results of the double-blind, RCT study by Rosenbaum on diabetic patients showed that leptin levels decreased at the end of the intervention in both ACB and plasma groups (73). The findings of the study conducted by Li et al. involving 134 patients with T2D showed that individuals receiving a combination of ACB and insulin experienced a greater decrease in TNF-α levels compared with the group receiving insulin alone (151). The results of Mo et al.’s study on newly diagnosed T2D patients showed that intake of ACB in this group for 1 year decreased TNF-α levels, but these changes were not significant compared with the group intake of metformin (127). The use of anti-diabetic drugs by reducing inflammation can decrease the risk of developing disorders and chronic diseases. Increasing insulin resistance as a pathophysiological disorder plays a role in the development and progression of diabetes and CVDs such as arteriosclerosis and is often associated with inflammation. Hyperglycemic conditions can lead to an increase in inflammatory cytokines and an increase in the expression of their genes. Thus far, the mechanisms by which ACB consumption directly affects inflammatory factors (such as TNF-α) have not been identified. The mechanisms of its indirect role include increasing insulin sensitivity in tissues and blood glucose control (127, 152–155). The results of a study on diabetic rats showed that intake of ACB through the signals regulated the MicroRNAs in the intestine, as well as controlling the blood glucose level through the MAPK pathway reduces inflammatory factors such as TNF-α (144).

The microbiota of the intestine is associated with chronic disorders such as obesity and inflammation (156). The protective efficacy of ACB versus cardiovascular disease can be due to the moderate growth of gut microbiota and inflammatory markers (157). ACB makes more SCFAs production in the intestine and stimulates potassium flow through binding to FFAR2 and GPR109A in intestinal cells, followed by hyperpolarization and activation of protein NLRP3 and release of IL-18, thus maintaining the integrity and repair of the intestinal barrier (158). Other mechanisms that play a role in reducing inflammation by SCFAs include inhibition of histone deacetylase and NF-kB in macrophages by butyrate, diminishing pro-inflammatory chemokines in dendritic cells (CXCL11, CXCL10, CXCL9, and CCL5), inhibition of cytokines induced by liposaccharides such as IL-6, and reducing PH to prevent the growth of harmful microorganisms (159–163). ACB can have immune suppressive effects through modulating the production of T helper 1 (Th1) and T helper 2 (Th2) (164). Insulin resistance in adipose tissue can cause inflammation and thus increase the agglomeration of pro-inflammatory macrophages. It can also activate pro-inflammatory macrophages through the generation of the *monocyte chemoattractant protein-1* (MCP1). In visceral adipose tissue from obese individuals, insulin resistance is associated with decreased insulin/mTORC2 signaling and increased MCP1 generation. Therefore, it seems likely that ACB can be effective in reducing the level of cytokines by improving insulin sensitivity (165). Fat hypertrophy, which occurs due to increased fat accumulation, can activate pro-inflammatory pathways such as NF-kB, which results in increased production of pro-inflammatory adipokines (166, 167). Inflammation caused by obesity can be caused by the increase in energy intake, which causes morphological and metabolic variations to appear in adipose tissue (168). TNF-α is secreted by fat tissue cells and TNF-α mRNA is associated with hyperinsulinemia. Since ACB is effective in reducing weight by preventing the storing of fats, controlling appetite (169), and decrement of energy intake (170), it can be effective in reducing the levels of TNF-α secreted from fat tissue.

The present systematic review and meta-analysis indicated that there were no significant effects between intake of ACB with ALP, AST, and ALT enzymes in T2D, T1D, and IGT patients. In double-blind RCT by Gentile et al. on 52 patients treated with 300 mg/day ACB, results have shown that there was no significant effect between intake of ACB with AST and ALT (69). ACB may have hepatic and cardiovascular safety, according to nationwide population-based longitudinal research in 32,531 T2D patients with end-stage renal disease (ESRD) who were identified from Taiwan’s National Health Insurance Research Database in 2000–2012 and followed up until 2013 (171). But some clinical trials have revealed the liver damage linked to the use of ACB in the general population with T2D, including asymptomatic increases of liver transaminases and jaundice (48, 172) and even in some case series studies (173–175). A recent meta-analysis of clinical trials found that there may be a dose–response relationship between the risk of hepatotoxicity and the use of glucosidase inhibitors (176). In these studies, it is worth noting that only laboratory measurements were reported as surrogate indicators, and no clinically significant liver damage events were observed. However, despite these findings, the underlying mechanism that would explain this result remains unclear. Further research is needed to better understand the potential effects of ACB on liver health and to elucidate the mechanisms involved.

Intake of ACB appears to be a significant diminution of body weight and BMI in T2D, T1D, and IGT populations by 1.26 kg and 0.65 kg/m^2^, respectively. According to the recommendations and guidelines, the accepted criterion for significant weight loss to achieve health benefits is a weight loss greater than or equal to 5% or 2 kg from the initial amount (177–180). In an old meta-analysis study by Hanefeld et al., the results of studies on T2D patients demonstrated that treatment with ACB can improve body weight (139). In a study by Hajiaghamohammadi et al., from a total of 62 patients with non-alcoholic steatohepatitis (NASH), 33 patients were treated with 100 mg/day ACB, and the results of this study for 10 weeks demonstrated that ACB can reduced body weight and BMI. Moreover, changes of body weight was significant between the ACB group and the group treated with ezetimibe, while BMI was not. In a recent meta-analysis study by Yu et al., overall, the results from five studies on 164 non-diabetic obese and overweight populations demonstrated that there was no significant difference in the outcome between the ACB group compared with the control group (24).

Consuming ACB can prevent the storing of fats by enhancing mRNA expression for peroxisome proliferator-activated receptor-γ (PPAR-γ), UCP-2, and abca1 in liver tissue and gain srebp1c, PPAR-γ, and PPAR-α in adipose tissue (181). The decrease in energy absorption due to the consumption of ACB is due to the fermentation of carbohydrates in the large intestine and the production of SCFAs (170). Another role of SCFAs is to regulate the mechanism of satiety; in this way, these compounds can act as signals to activate G-protein coupled receptors (such as G protein receptor 41 (Gpr41) and G protein receptor 43 (Gpr43)) and release leptin from adipose tissue, as well as the release of peptide YY and GLP-1 from the endocrine glands (182–185). In the hypothalamic arcuate nucleus GLP-1, peptide YY suppresses appetite-stimulating factors such as neuropeptide Y (NPY) and agouti-related peptide (AgRP), and on the other side, these raise cause-acting proopiomelanocortin (POMC)/cocaine and amphetamine-regulated transcript. Other roles of PYY and GLP-1 include delaying and suppressing the movements of the upper part of the digestive tract (169). SCFAs stimulate GPR41 in sympathetic system nodes, leading to an increase in norepinephrine, followed by an increase in the activity of the sympathetic system and an increase in energy expenditure (186). Carbohydrates can participate in the lipogenesis process as a substrate. Moreover, ACB reduces intestinal fatty acid synthesis by delaying glucose (52).

The findings of this study demonstrated that intake of ACB reduces TC, TG, and SBP by 2.26 mg/dL, 13.89 mg/dL, and 1.30 mmHg, respectively, in patients with T2D, T1D, and IGT. In another 2021 meta-analysis study by Wang et al., findings from 4 studies on 202 individuals showed that there is a significant effect between the reduction of SBP after a meal and the consumption of ACB (187). In a meta-analysis study by Hanefeld et al., the results of studies of randomized, double-blind, placebo-controlled T2D patients showed that treatment with ACB can ameliorate cardiovascular incidents, TG, and SBP in patients (139). In a meta-analysis study by Yu et al., overall, the results of four studies on LDL, SBP, and DBP and five studies on HDL and TG demonstrated a significant reduction in TG, whereas the reduction in HDL, LDL, SBP, and DBP was not significant in the intervention group compared with the placebo group (136).

The improvement in cardiovascular factors, such as lipid profile and blood pressure, may be attributed to several factors, including the enhancement of blood glucose levels, reduction in inflammatory factors, and weight loss. Postprandial hyperglycemia, specifically, has been associated with an increased risk of cardiovascular disease. This risk may be related to endothelial dysfunction and an increase in carotid intima-media thickness. ACB has shown promising results in ameliorating these disorders, suggesting its potential in addressing the underlying mechanisms and improving cardiovascular health (188–190). In addition, ACB can affect the activity of factor NFκB, and through this reduces the inflammatory response that is necessary for the formation of atherosclerotic plaque (191, 192). ACB can lead to a decrease in calorie intake and weight loss by reducing appetite or even inhibiting fat absorption (193), which can lead to a decrease in BP.

ACB drug can affect TG levels by reducing the generation of chylomicron remnant by defects in the synthesis of TG in the small intestine, as well as its efficacy on insulin levels and postprandial glucose levels (194, 195). In cell models of diabetes, increase glucose levels caused oxidative stress and cell damage in endothelial cells and neurons (196–199). Treating with ACB decreases the risk of CVD by improving the atherogenicity of LDL-c by alteration in fatty acid combination, reducing TG content, and decreasing oxidative susceptibility (200). Disruption of endothelial function by an inflammatory response such as oxidation of LDL-c, which causes the activation of PKC and NF-kB caused enhancement of conversion enzymes of the angiotensin II (Ang II) and inflammatory cytokines (201). SCFAs by PPARs regulate equilibrium among synthesis and oxidation of fatty acid and lipolysis in the tissues (148). SCFAs by activation of the hepatic cyclic adenosine 3′,5′-cyclic monophosphate (cAMP), protein kinase A (PKA), and enhanced oxidative metabolism inhibit the lipolysis process (202). Acetate is metabolized to acetyl-CoA and, in this way, its role in the process of lipogenesis, whereas propionate can suppress cholesterogenesis through interference with the enzyme of 3-hydroxy-3-methylglutaryl-CoA reductase (203). The intestinal microbiome plays a crucial role in enhancing the elimination and de-conjugation of bile acids. This process leads to an increased conversion of cholesterol to bile acids in the liver. As a result, serum cholesterol levels decrease (203). It appears to be another way of the efficacy of ACB in reducing BP due to weight loss. Abnormal distribution of fat-free acids in obese individuals can increase vascular adrenergic sensitivity (204). Fat-free acids suppress the Na^+^/K^+^ ATPase channel and the sodium pump increases vascular smooth muscle resistance (205). SCFAs participate in the regulation of BP through cell receptors including GPR43, GPR41, and olfactory receptor 78 (Olfr78) (206, 207). The increase in blood pressure resulting from the release of renin from the afferent arteriole, induced by short-chain fatty acids (SCFAs), is mediated by the interaction between Olfr78 and GPR43, with the vasodilator role of GPR43 counteracting its effects (208).

This study possesses several notable strengths. First, it encompassed all relevant double-blind RCTs that met the eligibility criteria. Second, it employed various analytical approaches, such as subgroup analysis, sensitivity analysis, GRADE assessment, and dose–response non-linear analysis. These methods ensured a comprehensive evaluation of the data. Third, the study took a comprehensive approach by considering all cardiovascular risk factors, enabling a thorough assessment. Additionally, the study had a substantial sample size, enhancing its statistical power. Another strength was the absence of language and time restrictions in the search strategy, ensuring inclusivity. Moreover, the study accounted for gender differences by analyzing adverse effect reports in trials. Finally, a high level of generalizability was achieved due to the inclusion of diverse studies conducted across multiple countries.

However, several limitations should be acknowledged in this study. First, some RCTs had limited follow-up periods, which may have affected the assessment of long-term effects. Second, high heterogeneity was observed among the included studies, potentially influencing the overall conclusions. Third, the study did not adequately account for important factors such as patient diet, physical activity, or smoking habits in the analyzed studies. Fourth, the study lacked information regarding participants’ full compliance with the intervention, which may have impacted the results. Additionally, variations in dosage and pharmacokinetics of ACB among individuals due to different drug manufacturers were not taken into consideration. Finally, the use of different kits and methods to measure biochemical parameters may have introduced variability, as intra- and inter-assay coefficients can differ and impact the results.

## Conclusion

The combined results from 95 randomized controlled trials (RCTs) indicate that the antidiabetic medication ACB has demonstrated positive effects on various parameters. These include reducing HbA1c, FPG, serum insulin, BMI, leptin, SBP, TC, TG, TNF-α, and body weight. Additionally, when used for more than 50 weeks, ACB has shown a significant impact in lowering TC levels. Furthermore, a dosage of 180 mg/day has proven to be particularly effective in reducing CRP levels in patients with T2D, T1D, and IGT. However, further research is required to fully understand the efficacy, mechanism, and functionality of ACB in managing metabolic disorders and different medical conditions.

## Author contributions

MoZ designed the study. MoZ and OA developed the search strategy. MoZ, MN-S, and OA extracted the data and conducted the analyses. MaZ, NR, and YA drafted the manuscript. MoZ and OA assessed the risk of bias of the meta-analyses. FS, OA, and MoZ interpreted the results. FS and OA revised manuscript. All authors contributed to the article and approved the submitted version.

## Conflict of interest

The authors declare that the research was conducted in the absence of any commercial or financial relationships that could be construed as a potential conflict of interest.

## Publisher’s note

All claims expressed in this article are solely those of the authors and do not necessarily represent those of their affiliated organizations, or those of the publisher, the editors and the reviewers. Any product that may be evaluated in this article, or claim that may be made by its manufacturer, is not guaranteed or endorsed by the publisher.

## Figures and Tables

**Table:** 

CVDs	cardiovascular disease
CRP	C reactive protein
BMI	body mass index
PICO	Participant Intervention Comparison/Control Outcome
TG	triglyceride
TC	total cholesterol
LDL	low-density lipoprotein
HDL	high-density lipoprotein
FBG	fasting blood glucose
T1D	type 1 diabetes mellitus
T2D	type 2 diabetes mellitus
HbA1c	hemoglobin A1c
HOMA-IR	homeostasis model assessment-insulin resistance
SBP	systolic blood pressure
DBP	diastolic blood pressure
IL-6	interleukin-6
WC	waist circumference
BMI	body mass index
AST	aspartate transaminase
ALT	alanine transaminase
GRADE	Grading of Recommendations Assessment, Development, and Evaluation
WMD	weighted mean difference
MAPK	mitogen-activated protein kinase
NF-kB	nuclear factor kappa B
TNF*-α*	tumor necrosis factor-alpha
PPARγ	peroxisome proliferator-activated receptor γ
cAMP	cyclic adenosine 3′,5′-cyclic monophosphate
GLUT4	glucose transporter type 4
IL6	interleukin 6
NF-kB	nuclear factor kappa B
OGTT	oral glucose tolerance test
IGT	impaired glucose tolerance
AGI	α-glucosidase-inhibiting
PRISMA	preferred reporting items for systematic reviews and meta-analyses
SEs	standard errors
SD	standard deviation
CI	confidence intervals
IQRs	interquartile ranges
PKC	protein kinase C activation
MCP1	*monocyte chemoattractant protein-1*
ESRD	end-stage renal disease
Gpr41	G protein receptor 41
Gpr43	G protein receptor 43
NPY	neuropeptide Y
Ang II	angiotensin II
PKA	protein kinase A
Olfr78	olfactory receptor 78
TEG1	thermoelectric generator 1
NF-kB	nuclear factor kappa B
TLRs	toll-like receptors
Th1	T helper 1
Th2	T helper 2
PPG	postprandial glucose

## References

[1] Roth GA Mensah GA Johnson CO Addolorato G Ammirati E Baddour LM . Global burden of cardiovascular diseases and risk factors, 1990-2019: update from the GBD 2019 study. J Am Coll Cardiol. (2020) 76:2982–3021. doi: 10.1016/j.jacc.2020.11.010, PMID: 33309175 PMC7755038

[2] Giedrimiene D King R. Abstract 207: burden of cardiovascular disease (CVD) on economic cost. Comparison of outcomes in US and Europe. Circ Cardiovasc Qual Outcomes. (2017) 10:A207. doi: 10.1161/circoutcomes.10.suppl_3.207

[3] Dal Canto E Ceriello A Rydén L Ferrini M Hansen TB Schnell O . Diabetes as a cardiovascular risk factor: an overview of global trends of macro and micro vascular complications. Eur J Prev Cardiol. (2019) 26:25–32. doi: 10.1177/2047487319878371, PMID: 31722562

[4] Ruiz-Moreno C Lara B Salinero JJ Brito de Souza D Ordovás JM Del Coso J. Time course of tolerance to adverse effects associated with the ingestion of a moderate dose of caffeine. Eur J Nutr. (2020) 59:3293–302. doi: 10.1007/s00394-019-02167-2, PMID: 31900579

[5] American Diabetes Association. Diagnosis and classification of diabetes mellitus. Diabetes Care. (2010) 33:S62–9. doi: 10.2337/dc10-S062, PMID: 20042775 PMC2797383

[6] Rao SS Disraeli P McGregor T. Impaired glucose tolerance and impaired fasting glucose. Am Fam Physician. (2004) 69:1961–8. PMID: 15117017

[7] Wu L Parhofer KG. Diabetic dyslipidemia. Metabolism. (2014) 63:1469–79. doi: 10.1016/j.metabol.2014.08.010, PMID: 25242435

[8] Forlani G Di Bonito P Mannucci E Capaldo B Genovese S Orrasch M . Prevalence of elevated liver enzymes in type 2 diabetes mellitus and its association with the metabolic syndrome. J Endocrinol Investig. (2008) 31:146–52. doi: 10.1007/BF03345581, PMID: 18362506

[9] Calle MC Fernandez ML. Inflammation and type 2 diabetes. Diabetes Metab. (2012) 38:183–91. doi: 10.1016/j.diabet.2011.11.006, PMID: 22252015

[10] Noormohammadi M Eslamian G Malek S Shoaibinobarian N Mirmohammadali SN. The association between fertility diet score and polycystic ovary syndrome: a case-control study. Health Care Women Int. (2022) 43:70–84. doi: 10.1080/07399332.2021.1886298, PMID: 33797335

[11] Shoaibinobarian N Eslamian G Noormohammadi M Malek S Rouhani S Mirmohammadali SN. Dietary total antioxidant capacity and risk of polycystic ovary syndrome: a case-control study. Int J Fertil Steril. (2022) 16:200–5. doi: 10.22074/ijfs.2021.526579.1107, PMID: 36029057 PMC9396001

[12] Gupta S Bansal S. Does a rise in BMI cause an increased risk of diabetes?: evidence from India. PloS One. (2020) 15:e0229716. doi: 10.1371/journal.pone.0229716, PMID: 32236106 PMC7112218

[13] Teufel F Seiglie JA Geldsetzer P Theilmann M Marcus ME Ebert C . Body-mass index and diabetes risk in 57 low-income and middle-income countries: a cross-sectional study of nationally representative, individual-level data in 685 616 adults. Lancet (London, England). (2021) 398:238–48. doi: 10.1016/S0140-6736(21)00844-8 34274065 PMC8336025

[14] Liu G Li Y Hu Y Zong G Li S Rimm EB . Influence of lifestyle on incident cardiovascular disease and mortality in patients with diabetes mellitus. J Am Coll Cardiol. (2018) 71:2867–76. doi: 10.1016/j.jacc.2018.04.027, PMID: 29929608 PMC6052788

[15] Wahid A Manek N Nichols M Kelly P Foster C Webster P . Quantifying the association between physical activity and cardiovascular disease and diabetes: a systematic review and meta-analysis. J Am Heart Assoc. (2016) 5:e002495. doi: 10.1161/JAHA.115.002495, PMID: 27628572 PMC5079002

[16] Archundia Herrera MC Subhan FB Chan CB. Dietary patterns and cardiovascular disease risk in people with type 2 diabetes. Curr Obes Rep. (2017) 6:405–13. doi: 10.1007/s13679-017-0284-5, PMID: 29063379

[17] Jafari N Shoaibinobarian N Dehghani A Rad A Mirmohammadali SN Alaeian MJ . The effects of purslane consumption on glycemic control and oxidative stress: a systematic review and dose–response meta-analysis. Food Sci Nutr. (2023) 11:2530–46. doi: 10.1002/fsn3.3311, PMID: 37324837 PMC10261734

[18] Mirmohammadali SN Rosenkranz SK. Dietary phytochemicals, gut microbiota composition, and health outcomes in human and animal models. Biosci Microbiota Food Health. (2023) 42:152–71. doi: 10.12938/bmfh.2022-078, PMID: 37404568 PMC10315191

[19] Patoulias D Stavropoulos K Imprialos K Athyros V Doumas M Karagiannis A. Pharmacological management of cardiac disease in patients with type 2 diabetes: insights into clinical practice. Curr Vasc Pharmacol. (2020) 18:125–38. doi: 10.2174/1570161117666190426162746, PMID: 32013815

[20] DiNicolantonio JJ Bhutani J O'Keefe JH. Acarbose: safe and effective for lowering postprandial hyperglycaemia and improving cardiovascular outcomes. Open Heart. (2015) 2:e000327. doi: 10.1136/openhrt-2015-000327, PMID: 26512331 PMC4620230

[21] Yang W Liu J Shan Z Tian H Zhou Z Ji Q . Acarbose compared with metformin as initial therapy in patients with newly diagnosed type 2 diabetes: an open-label, non-inferiority randomised trial. Lancet Diabetes Endocrinol. (2014) 2:46–55. doi: 10.1016/S2213-8587(13)70021-4, PMID: 24622668

[22] Chiasson JL Josse RG Gomis R Hanefeld M Karasik A Laakso M. Acarbose treatment and the risk of cardiovascular disease and hypertension in patients with impaired glucose tolerance: the STOP-NIDDM trial. JAMA. (2003) 290:486–94. doi: 10.1001/jama.290.4.486, PMID: 12876091

[23] Mannucci E Gallo M Pintaudi B Targher G Candido R Giaccari A . All-cause mortality and cardiovascular events in patients with type 2 diabetes treated with alpha-glucosidase inhibitors: a meta-analysis of randomized controlled trials. Nutr Metab Cardiovasc Dis. (2022) 32:511–4. doi: 10.1016/j.numecd.2021.10.010, PMID: 34893404

[24] Yu AQ Le J Huang WT Li B Liang HX Wang Q . The effects of Acarbose on non-diabetic overweight and obese patients: a meta-analysis. Adv Ther. (2021) 38:1275–89. doi: 10.1007/s12325-020-01602-9, PMID: 33421022

[25] Schnell O Weng J Sheu WH Watada H Kalra S Soegondo S . Acarbose reduces body weight irrespective of glycemic control in patients with diabetes: results of a worldwide, non-interventional, observational study data pool. J Diabetes Complicat. (2016) 30:628–37. doi: 10.1016/j.jdiacomp.2016.01.023, PMID: 26935335

[26] Hu R Li Y Lv Q Wu T Tong N. Acarbose monotherapy and type 2 diabetes prevention in eastern and Western prediabetes: an ethnicity-specific Meta-analysis. Clin Ther. (2015) 37:1798–812. doi: 10.1016/j.clinthera.2015.05.504, PMID: 26118669

[27] Liu Z Yang D Xu W Lv J Lin H Liu Z . 771-P: the effect of adding acarbose to insulin therapy in type 1 diabetes mellitus: a systematic review and meta-analysis. Diabetes. (2021) 70:771–P. doi: 10.2337/db21-771-P, PMID: 34649926

[28] Page MJ McKenzie JE Bossuyt PM Boutron I Hoffmann TC Mulrow CD . The PRISMA 2020 statement: an updated guideline for reporting systematic reviews. Int J Surg. (2021) 88:105906. doi: 10.1016/j.ijsu.2021.105906, PMID: 33789826

[29] Sciacchitano S Lavra L Morgante A Ulivieri A Magi F De Francesco GP . Galectin-3: one molecule for an alphabet of diseases, from a to Z. Int J Mol Sci. (2018) 19:379. doi: 10.3390/ijms19020379, PMID: 29373564 PMC5855601

[30] Der Simonian R Laird N. Meta-analysis in clinical trials. Control Clin Trials. (1986) 7:177–88. 3802833 10.1016/0197-2456(86)90046-2

[31] Asbaghi O Sadeghian M Mozaffari-Khosravi H Maleki V Shokri A Hajizadeh-Sharafabad F . The effect of vitamin d-calcium co-supplementation on inflammatory biomarkers: a systematic review and meta-analysis of randomized controlled trials. Cytokine. (2020) 129:155050. doi: 10.1016/j.cyto.2020.155050, PMID: 32113022

[32] Hozo S Djulbegovic B Hozo I. Estimating the mean and variance from the median, range, and the size of a sample. BMC Med Res Methodol (2005) 5:13. doi: 10.1186/1471-2288-5-13 15840177 10.1186/1471-2288-5-13 PMC1097734

[33] Berven G Bye A Hals O Blankson H Fagertun H Thom E . Safety of conjugated linoleic acid (CLA) in overweight or obese human volunteers. Eur J Lipid Sci Technol. (2000) 102:455–62. doi: 10.1002/1438-9312(200008)102:7<455::AID-EJLT455>3.0.CO;2-V, PMID: 37392524

[34] Fu R Gartlehner G Grant M Shamliyan T Sedrakyan A Wilt T . Conducting quantitative synthesis when comparing medical interventions: AHRQ and the effective health care program. J Clin Epidemiol. (2011) 64:1187–97. doi: 10.1016/j.jclinepi.2010.08.010, PMID: 21477993

[35] Naumann E Carpentier YA Saebo A Lassel TS Chardigny J-M Sébédio J-L . Cis-9, trans-11 and trans-10, cis-12 conjugated linoleic acid (CLA) do not affect the plasma lipoprotein profile in moderately overweight subjects with LDL phenotype B. Atherosclerosis. (2006) 188:167–74. doi: 10.1016/j.atherosclerosis.2005.10.019, PMID: 16289507

[36] Namazi N Larijani B Azadbakht L. Low-carbohydrate-diet score and its association with the risk of diabetes: a systematic review and meta-analysis of cohort studies. Horm Metab Res. (2017) 49:565–71. doi: 10.1055/s-0043-112347 28679144

[37] Brondani L Assmann T de Souza B Bouças A Canani L Crispim D. Meta-analysis reveals the association of common variants in the uncoupling protein (UCP) 1-3 genes with body mass index variability. PloS One. (2014) 9:e96411. doi: 10.1371/journal.pone.0096411, PMID: 24804925 PMC4013025

[38] Begg CB Mazumdar M. Operating characteristics of a rank correlation test for publication bias. Biometrics. (1994) 50:1088–101. doi: 10.2307/2533446, PMID: 7786990

[39] Egger M Davey Smith G Schneider M Minder C. Bias in meta-analysis detected by a simple, graphical test. BMJ (Clin Res Ed). (1997) 315:629–34. doi: 10.1136/bmj.315.7109.629, PMID: 9310563 PMC2127453

[40] Duval S. The trim and fill method In: Rothstein HR Sutton AJ Borenstein M, editors. Publication bias in meta-analysis: prevention, assessment and adjustments. Hoboken, NJ: John Wiley & Sons, Ltd. (2005). 127–44.

[41] Xu C Doi SA. The robust error meta-regression method for dose–response meta-analysis. Int J Evid Based Healthc. (2018) 16:138–44. doi: 10.1097/XEB.0000000000000132 29251651

[42] Xie Y Gou L Peng M Zheng J Chen L. Effects of soluble fiber supplementation on glycemic control in adults with type 2 diabetes mellitus: a systematic review and meta-analysis of randomized controlled trials. Clin Nutr. (2021) 40:1800–10. doi: 10.1016/j.clnu.2020.10.032, PMID: 33162192

[43] Guyatt G Oxman A Vist G Kunz R Falck-Ytter Y Alonso-Coello P . GRADE: an emerging consensus on rating quality of evidence and strength of recommendations. BMJ. (2008) 336:924–6. doi: 10.1136/bmj.39489.470347.AD, PMID: 18436948 PMC2335261

[44] Akazawa Y Koide M Oishi M Azuma T Tashiro S. Clinical usefulness of acarbose and fiber in the treatment of diabetes mellitus. Therapeutics. (1982) 36:848–9.

[45] Scott R Knowles R Beaven D. Treatment of poorly controlled non-insulin-dependent diabetic patients with acarbose. Aust NZ J Med. (1984) 14:649–54. doi: 10.1111/j.1445-5994.1984.tb05018.x, PMID: 6397178

[46] Hotta N Kakuta H Sano T Matsumae H Yamada H Kitazawa S . Long-term effect of acarbose on glycaemic control in non-insulin-dependent diabetes mellitus: a placebo-controlled double-blind study. Diabet Med. (1993) 10:134–8. doi: 10.1111/j.1464-5491.1993.tb00030.x, PMID: 8458189

[47] Jenney A Proietto J O'Dea K Nankervis A Traianedes K D'Embden H. Low-dose acarbose improves glycemic control in NIDDM patients without changes in insulin sensitivity. Diabetes Care. (1993) 16:499–502. doi: 10.2337/diacare.16.2.499, PMID: 8432223

[48] Coniff RF Shapiro JA Seaton TB Hoogwerf BJ Hunt JA. A double-blind placebo-controlled trial evaluating the safety and efficacy of acarbose for the treatment of patients with insulin-requiring type II diabetes. Diabetes Care. (1995) 18:928–32. doi: 10.2337/diacare.18.7.928, PMID: 7555551

[49] Coniff RF Shapiro JA Seaton TB. Long-term efficacy and safety of Acarbose in the treatment of obese subjects with non—insulin-dependent diabetes mellitus. Arch Intern Med. (1994) 154:2442–8. doi: 10.1001/archinte.1994.00420210080009, PMID: 7979840

[50] Wolever T Radmard R Chiasson JL Hunt J Josse R Palmason C . One-year acarbose treatment raises fasting serum acetate in diabetic patients. Diabet Med. (1995) 12:164–72. doi: 10.1111/j.1464-5491.1995.tb00448.x, PMID: 7743764

[51] Chiasson J-L Josse RG Leiter LA Mihic M Nathan DM Palmason C . The effect of acarbose on insulin sensitivity in subjects with impaired glucose tolerance. Diabetes Care. (1996) 19:1190–3. doi: 10.2337/diacare.19.11.1190, PMID: 8908378

[52] Bayraktar F Hamulu F Özgen A Yilmaz C Tüzün M Kabalak T. Acarbose treatment in obesity: a controlled study. Eat Weight Disord. (1998) 3:46–9. doi: 10.1007/BF03339987, PMID: 11234255

[53] Bayraktar M Van Thiel DH Adalar N. A comparison of acarbose versus metformin as an adjuvant therapy in sulfonylurea-treated NIDDM patients. Diabetes Care. (1996) 19:252–4. doi: 10.2337/diacare.19.3.252, PMID: 8742572

[54] Costa B Pinol C. Acarbose in ambulatory treatment of non-insulin-dependent diabetes mellitus associated to imminent sulfonylurea failure: a randomised-multicentric trial in primary health-care. Diabetes and Acarbose Research Group. Diabetes Res Clin Pract. (1997) 38:33–40. doi: 10.1016/S0168-8227(97)00083-1, PMID: 9347244

[55] Noda K Umeda F Nawata H. Effect of acarbose on glucose intolerance in patients with non-insulin-dependent diabetes mellitus. Diabetes Res Clin Pract. (1997) 37:129–36. doi: 10.1016/S0168-8227(97)00066-1, PMID: 9279483

[56] Hoffmann J. Efficacy of 24-week monotherapy with acarbose, metformin, or placebo in dietary-treated NIDDM patients: the Essen-II study. Am J Med. (1997) 103:483–90. doi: 10.1016/S0002-9343(97)00252-0, PMID: 9428831

[57] Soonthornpun S Rattarasarn C Thamprasit A Leetanaporn K. Effect of acarbose in treatment of type II diabetes mellitus: a double-blind, crossover, placebo-controlled trial. J Med Assoc Thail. (1998) 81:195–200. PMID: 9623011

[58] Chan JC Chan K-WA Ho LL Fuh MM Horn LC Sheaves R . An Asian multicenter clinical trial to assess the efficacy and tolerability of acarbose compared with placebo in type 2 diabetic patients previously treated with diet. Diabetes Care. (1998) 21:1058–61. doi: 10.2337/diacare.21.7.1058, PMID: 9653595

[59] Guagnano MT Loggia FD Pace-Palitti V Spoltore R Capitanio R Sensi S. Case-control study in non-insulin-dependent diabetes mellitus (NIDDM) subjects treated with acarbose. Drug Dev Res. (1998) 43:128–31. doi: 10.1002/(SICI)1098-2299(199802)43:2<128::AID-DDR6>3.0.CO;2-M, PMID: 26331290

[60] Lam KS Tiu S Tsang M Ip T Tam SC. Acarbose in NIDDM patients with poor control on conventional oral agents: a 24-week placebo-controlled study. Diabetes Care. (1998) 21:1154–8. doi: 10.2337/diacare.21.7.1154, PMID: 9653611

[61] Sels J Verdonk H Wolffenbuttel B. Effects of acarbose (Glucobay®) in persons with type 1 diabetes: a multicentre study. Diabetes Res Clin Pract. (1998) 41:139–45. doi: 10.1016/S0168-8227(98)00070-9, PMID: 9789720

[62] Fischer S Patzak A Rietzsch H Schwanebeck U Köhler C Wildbrett J . Influence of treatment with acarbose or glibenclamide on insulin sensitivity in type 2 diabetic patients. Diabetes Obes Metab. (2003) 5:38–44. doi: 10.1046/j.1463-1326.2003.00239.x, PMID: 12542723

[63] Fischer S Hanefeld M Spengler M Boehme K Temelkova-Kurktschiev T. European study on dose-response relationship of acarbose as a first-line drug in non-insulin-dependent diabetes mellitus: efficacy and safety of low and high doses. Acta Diabetol. (1998) 35:34–40. doi: 10.1007/s005920050098, PMID: 9625287

[64] Standl E Baumgartl H Füchtenbusch M Stemplinger J. Effect of acarbose on additional insulin therapy in type 2 diabetic patients with late failure of sulphonylurea therapy. Diabetes Obes Metab. (1999) 1:215–20. doi: 10.1046/j.1463-1326.1999.00021.x, PMID: 11228756

[65] Lopez-Alvarenga J Aguilar-Salinas C Velasco-Perez M Arita-Melzer O Guillen L Wong B . Acarbose vs. bedtime NPH insulin in the treatment of secondary failures to sulphonylurea-metformin therapy in type 2 diabetes mellitus. Diabetes Obes Metab. (1999) 1:29–35. doi: 10.1046/j.1463-1326.1999.00007.x, PMID: 11221809

[66] Salman S Salman F Satman I Sengül A Demirel HÖ Karsıdag K . Comparison of acarbose and gliclazide as first-line agents in patients with type 2 diabetes. Curr Med Res Opin. (2000) 16:296–306. doi: 10.1185/0300799019117009, PMID: 11268714

[67] Meneilly GS Ryan EA Radziuk J Lau D Yale J-F Morais J . Effect of acarbose on insulin sensitivity in elderly patients with diabetes. Diabetes Care. (2000) 23:1162–7. doi: 10.2337/diacare.23.8.1162, PMID: 10937515

[68] Halimi S Le Berre M Grange V. Efficacy and safety of acarbose add-on therapy in the treatment of overweight patients with type 2 diabetes inadequately controlled with metformin: a double-blind, placebo-controlled study. Diabetes Res Clin Pract. (2000) 50:49–56. doi: 10.1016/S0168-8227(00)00163-7, PMID: 10936668

[69] Gentile S Turco S Guarino G Oliviero B Annunziata S Cozzolino D . Effect of treatment with acarbose and insulin in patients with non-insulin-dependent diabetes mellitus associated with non-alcoholic liver cirrhosis. Diabetes Obes Metab. (2001) 3:33–40. doi: 10.1046/j.1463-1326.2001.00103.x, PMID: 11213597

[70] Ko GT Tsang C-C Ng C-W Wai HP Kan EC. Use of acarbose or bedtime insulin after failure of treatment with conventional oral antidiabetics. Clin Drug Investig. (2001) 21:401–8. doi: 10.2165/00044011-200121060-00002, PMID: 37407175

[71] Takei I Miyamoto K Funae O Ohashi N Meguro S Tokui M . Secretion of GIP in responders to acarbose in obese type 2 (NIDDM) patients. J Diabetes Complicat. (2001) 15:245–9. doi: 10.1016/S1056-8727(01)00148-9, PMID: 11522498

[72] Vichayanrat A Ploybutr S Tunlakit M Watanakejorn P. Efficacy and safety of voglibose in comparison with acarbose in type 2 diabetic patients. Diabetes Res Clin Pract. (2002) 55:99–103. doi: 10.1016/S0168-8227(01)00286-8, PMID: 11796175

[73] Rosenbaum P Peres RB Zanella MT Ferreira SRG. Improved glycemic control by acarbose therapy in hypertensive diabetic patients: effects on blood pressure and hormonal parameters. Braz J Med Biol Res. (2002) 35:877–84. doi: 10.1590/S0100-879X2002000800004, PMID: 12185379

[74] Hanefeld M Haffner S Menschikowski M Koehler C Temelkova-Kurktschiev T Wildbrett J . Different effects of acarbose and glibenclamide on proinsulin and insulin profiles in people with type 2 diabetes. Diabetes Res Clin Pract. (2002) 55:221–7. doi: 10.1016/S0168-8227(01)00347-3, PMID: 11850098

[75] Rosenthal JH Mauersberger H. Effects on blood pressure of the α-glucosidase inhibitor acarbose compared with the insulin enhancer glibenclamide in patients with hypertension and type 2 diabetes mellitus. Clin Drug Investig. (2002) 22:695–701. doi: 10.2165/00044011-200222100-00006

[76] Göke B. Improved glycemic control and lipid profile in a randomized study of pioglitazone compared with acarbose in patients with type 2 diabetes mellitus. Treat Endocrinol. (2002) 1:329–36. doi: 10.2165/00024677-200201050-00005, PMID: 15832486

[77] Pan C-Y Gao Y Chen J-W Luo B-Y Fu Z-Z Lu J-M . Efficacy of acarbose in Chinese subjects with impaired glucose tolerance. Diabetes Res Clin Pract. (2003) 61:183–90. doi: 10.1016/S0168-8227(03)00117-7, PMID: 12965108

[78] Bachmann W Petzinna D Raptis SA Wascher T Westermeier T. Long-term improvement of metabolic control by acarbose in type 2 diabetes patients poorly controlled with maximum sulfonylurea therapy. Clin Drug Investig. (2003) 23:679–86. doi: 10.2165/00044011-200323100-00007, PMID: 17535083

[79] Lin BJ Wu H-P Huang H Huarng J Sison A Bin Abdul Kadir DK . Efficacy and tolerability of acarbose in Asian patients with type 2 diabetes inadequately controlled with diet and sulfonylureas. J Diabetes Complicat. (2003) 17:179–85. doi: 10.1016/S1056-8727(02)00258-1, PMID: 12810240

[80] Josse R Chiasson J-L Ryan E Lau D Ross S Yale J-F . Acarbose in the treatment of elderly patients with type 2 diabetes. Diabetes Res Clin Pract. (2003) 59:37–42. doi: 10.1016/S0168-8227(02)00176-6, PMID: 12482640

[81] Hwu C-M Ho L-T Fuh MM Siu SC Sutanegara D Piliang S . Acarbose improves glycemic control in insulin-treated Asian type 2 diabetic patients: results from a multinational, placebo-controlled study. Diabetes Res Clin Pract. (2003) 60:111–8. doi: 10.1016/S0168-8227(03)00015-9, PMID: 12706319

[82] Watanabe K Uchino H Ohmura C Tanaka Y Onuma T Kawamori R. Different effects of two α-glucosidase inhibitors, acarbose and voglibose, on serum 1, 5-anhydroglucitol (1, 5AG) level. J Diabetes Complicat. (2004) 18:183–6. doi: 10.1016/S1056-8727(03)00055-2, PMID: 15145332

[83] van de Laar FA Lucassen PL Kemp J van de Lisdonk EH van Weel C Rutten GE. Is acarbose equivalent to tolbutamide as first treatment for newly diagnosed type 2 diabetes in general practice?: a randomised controlled trial. Diabetes Res Clin Pract. (2004) 63:57–65. doi: 10.1016/j.diabres.2003.08.006, PMID: 14693413

[84] Göke B Lübben G Bates P. Coefficient of β-cell failure in patients with type 2 diabetes treated with pioglitazone or acarbose. Exp Clin Endocrinol Diabetes. (2004) 112:115–7. doi: 10.1055/s-2004-815767, PMID: 15031778

[85] Yajima K Shimada A Hirose H Kasuga A Saruta T. "Low dose" metformin improves hyperglycemia better than acarbose in type 2 diabetics. Rev Diabet Stud. (2004) 1:89. doi: 10.1900/RDS.2004.1.89, PMID: 17491670 PMC1783540

[86] Gentile S Guarino G Romano M Alagia IA Fierro M Annunziata S . A randomized controlled trial of acarbose in hepatic encephalopathy. Clin Gastroenterol Hepatol. (2005) 3:184–91. doi: 10.1016/S1542-3565(04)00667-6, PMID: 15704053

[87] Wagner H Degerblad M Thorell A Nygren J Stahle A Kuhl J . Combined treatment with exercise training and acarbose improves metabolic control and cardiovascular risk factor profile in subjects with mild type 2 diabetes. Diabetes Care. (2006) 29:1471–7. doi: 10.2337/dc05-2513, PMID: 16801564

[88] Suzuki T Oba K Futami S Suzuki K Ouchi M Igari Y . Blood glucose-lowering activity of colestimide in patients with type 2 diabetes and hypercholesterolemia: a case-control study comparing colestimide with acarbose. J Nippon Med Sch. (2006) 73:277–84. doi: 10.1272/jnms.73.277, PMID: 17106179

[89] Schnell O Mertes G Standl E Group AICS. Acarbose and metabolic control in patients with type 2 diabetes with newly initiated insulin therapy. Diabetes Obes Metab. (2007) 9:853–8. doi: 10.1111/j.1463-1326.2006.00666.x, PMID: 17924867

[90] Yilmaz H Gursoy A Sahin M Guvener Demirag N. Comparison of insulin monotherapy and combination therapy with insulin and metformin or insulin and rosiglitazone or insulin and acarbose in type 2 diabetes. Acta Diabetol. (2007) 44:187–92. doi: 10.1007/s00592-007-0004-9, PMID: 17726570

[91] Oyama T Saiki A Endoh K Ban N Nagayama D Ohhira M . Effect of acarbose, an alpha-glucosidase inhibitor, on serum lipoprotein lipase mass levels and common carotid artery intima-media thickness in type 2 diabetes mellitus treated by sulfonylurea. J Atheroscler Thromb. (2008) 15:154–9. doi: 10.5551/jat.E549, PMID: 18603822

[92] Hasegawa G Kajiyama S Tanaka T Imai S Kozai H Fujinami A . The α-glucosidase inhibitor acarbose reduces the net electronegative charge of low-density lipoprotein in patients with newly diagnosed type 2 diabetes. Clin Chim Acta. (2008) 390:110–4. doi: 10.1016/j.cca.2008.01.005, PMID: 18230353

[93] Nijpels G Boorsma W Dekker J Kostense P Bouter L Heine R. A study of the effects of acarbose on glucose metabolism in patients predisposed to developing diabetes: the Dutch acarbose intervention study in persons with impaired glucose tolerance (DAISI). Diabetes Metab Res Rev. (2008) 24:611–6. doi: 10.1002/dmrr.839, PMID: 18756586

[94] Pan C Yang W Barona J Wang Y Niggli M Mohideen P . Comparison of vildagliptin and acarbose monotherapy in patients with type 2 diabetes: a 24-week, double-blind, randomized trial. Diabet Med. (2008) 25:435–41. doi: 10.1111/j.1464-5491.2008.02391.x, PMID: 18341596

[95] Derosa G D'Angelo A Salvadeo SA Ferrari I Fogari E Gravina A . Modulation of adipokines and vascular remodelling markers during OGTT with acarbose or pioglitazone treatment. Biomed Pharmacother. (2009) 63:723–33. doi: 10.1016/j.biopha.2009.04.044, PMID: 19906504

[96] Derosa G Salvadeo SA D’Angelo A Ferrari I Mereu R Palumbo I . Metabolic effect of repaglinide or acarbose when added to a double oral antidiabetic treatment with sulphonylureas and metformin: a double-blind, cross-over, clinical trial. Curr Med Res Opin. (2009) 25:607–15. doi: 10.1185/03007990802711024, PMID: 19232035

[97] Hanefeld M Schaper F Koehler C Bergmann S Ugocsai P Stelzer J . Effect of acarbose on postmeal mononuclear blood cell response in patients with early type 2 diabetes: the AI (I) DA study. Horm Metab Res. (2009) 41:132–6. doi: 10.1055/s-0028-1119407 19214923

[98] Koyasu M Ishii H Watarai M Takemoto K Inden Y Takeshita K . Impact of acarbose on carotid intima-media thickness in patients with newly diagnosed impaired glucose tolerance or mild type 2 diabetes mellitus: a one-year, prospective, randomized, open-label, parallel-group study in Japanese adults with established coronary artery disease. Clin Ther. (2010) 32:1610–7. doi: 10.1016/j.clinthera.2010.07.015 20974318

[99] Bao YQ Zhou J Zhou M Cheng YJ Lu W Pan XP . Glipizide controlled-release tablets, with or without acarbose, improve glycaemic variability in newly diagnosed type 2 diabetes. Clin Exp Pharmacol Physiol. (2010) 37:564–8. doi: 10.1111/j.1440-1681.2010.05361.x, PMID: 20082624

[100] Rudovich NN Weickert MO Pivovarova O Bernigau W Pfeiffer AF. Effects of acarbose treatment on markers of insulin sensitivity and systemic inflammation. Diabetes Technol Ther. (2011) 13:615–23. doi: 10.1089/dia.2010.0235, PMID: 21488802

[101] Derosa G Maffioli P Ferrari I Fogari E D'Angelo A Palumbo I . Acarbose actions on insulin resistance and inflammatory parameters during an oral fat load. Eur J Pharmacol. (2011) 651:240–50. doi: 10.1016/j.ejphar.2010.11.015, PMID: 21118681

[102] Wang J-S Lin S-D Lee W-J Su S-L Lee I-T Tu S-T . Effects of acarbose versus glibenclamide on glycemic excursion and oxidative stress in type 2 diabetic patients inadequately controlled by metformin: a 24-week, randomized, open-label, parallel-group comparison. Clin Ther. (2011) 33:1932–42. doi: 10.1016/j.clinthera.2011.10.014, PMID: 22078152

[103] Hirano M Nakamura T Obata J-e Fujioka D Saito Y Kawabata K-i . Early improvement in carotid plaque echogenicity by acarbose in patients with acute coronary syndromes. Circ J. (2012) 76:1452–60. doi: 10.1253/circj.cj-11-1524 22453003

[104] Hajiaghamohammadi AA Miroliaee A Samimi R Alborzi F Ziaee A. A comparison of ezetimibe and acarbose in decreasing liver transaminase in nonalcoholic fatty liver disease: a randomized clinical trial. Govaresh. (2013) 18:186–90.

[105] Zheng F Yin X Lu W Zhou J Yuan H Li H. Improved post-prandial ghrelin response by nateglinide or acarbose therapy contributes to glucose stability in type 2 diabetic patients. J Endocrinol Investig. (2013) 36:489–96. doi: 10.3275/8811, PMID: 23324437

[106] Wang H Ni Y Yang S Li H Li X Feng B. The effects of gliclazide, metformin, and acarbose on body composition in patients with newly diagnosed type 2 diabetes mellitus. Curr Ther Res. (2013) 75:88–92. doi: 10.1016/j.curtheres.2013.10.002, PMID: 24465050 PMC3898190

[107] Patel Y Kirkman M Considine R Hannon T Mather K. Effect of acarbose to delay progression of carotid intima–media thickness in early diabetes. Diabetes Metab Res Rev. (2013) 29:582–91. doi: 10.1002/dmrr.2433, PMID: 23908125 PMC4062388

[108] Lee MY Choi DS Lee MK Lee HW Park TS Kim DM . Comparison of acarbose and voglibose in diabetes patients who are inadequately controlled with basal insulin treatment: randomized, parallel, open-label, active-controlled study. J Korean Med Sci. (2014) 29:90–7. doi: 10.3346/jkms.2014.29.1.90, PMID: 24431911 PMC3890482

[109] Chen P-H Tsai Y-T Wang J-S Lin S-D Lee W-J Su S-L . Post-meal β-cell function predicts the efficacy of glycemic control in patients with type 2 diabetes inadequately controlled by metformin monotherapy after addition of glibenclamide or acarbose. Diabetol Metab Syndr. (2014) 6:68. doi: 10.1186/1758-5996-6-68 24932223 PMC4057801

[110] Sugihara H Nagao M Harada T Nakajima Y Tanimura-Inagaki K Okajima F . Comparison of three α-glucosidase inhibitors for glycemic control and bodyweight reduction in Japanese patients with obese type 2 diabetes. J Diabetes Investig. (2014) 5:206–12. doi: 10.1111/jdi.12135, PMID: 24843762 PMC4023585

[111] Su B Liu H Li J Sunli Y Liu B Liu D . Acarbose treatment affects the serum levels of inflammatory cytokines and the gut content of bifidobacteria in C hinese patients with type 2 diabetes mellitus: 阿卡波糖对中国 2 型糖尿病患者炎症因子及粪便双歧杆菌水平的作用. J Diabetes. (2015) 7:729–39. doi: 10.1111/1753-0407.12232, PMID: 25327485

[112] Pan Q Xu Y Yang N Gao X Liu J Yang W . Comparison of acarbose and metformin on albumin excretion in patients with newly diagnosed type 2 diabetes: a randomized controlled trial. Medicine. (2016) 95:e3247. doi: 10.1097/MD.0000000000003247, PMID: 27057866 PMC4998782

[113] Li M Huang X Ye H Chen Y Yu J Yang J . Randomized, double-blinded, double-dummy, active-controlled, and multiple-dose clinical study comparing the efficacy and safety of mulberry twig (Ramulus Mori, Sangzhi) alkaloid tablet and acarbose in individuals with type 2 diabetes mellitus. Evid Based Complement Alternat Med. (2016) 2016:7121356. doi: 10.1155/2016/7121356, PMID: 27547230 PMC4980533

[114] Yun P Du A-m Chen X-j Liu J-c Xiao H. Effect of acarbose on long-term prognosis in acute coronary syndromes patients with newly diagnosed impaired glucose tolerance. J Diabetes Res. (2016) 2016:1602083. doi: 10.1155/2016/1602083, PMID: 26770983 PMC4684859

[115] Chen Y-H Tarng D-C Chen H-S. Renal outcomes of pioglitazone compared with acarbose in diabetic patients: a randomized controlled study. PLoS One. (2016) 11:e0165750. doi: 10.1371/journal.pone.0167321, PMID: 27812149 PMC5094682

[116] Sun W Zeng C Liao L Chen J Wang Y. Comparison of acarbose and metformin therapy in newly diagnosed type 2 diabetic patients with overweight and/or obesity. Curr Med Res Opin. (2016) 32:1389–96. doi: 10.1080/03007995.2016.1176013, PMID: 27052634

[117] Shi L Zhu J Yang P Tang X Yu W Pan C . Comparison of exenatide and acarbose on intra-abdominal fat content in patients with obesity and type-2 diabetes: a randomized controlled trial. Obes Res Clin Pract. (2017) 11:607–15. doi: 10.1016/j.orcp.2017.01.003, PMID: 28161303

[118] Ziaee A Esmailzadehha N Honardoost M. Comparison of adjunctive therapy with metformin and acarbose in patients with type-1 diabetes mellitus. Pakistan J Med Sci. (2017) 33:686. doi: 10.12669/pjms.333.12669, PMID: 28811795 PMC5510127

[119] Du J Liang L Fang H Xu F Li W Shen L . Efficacy and safety of saxagliptin compared with acarbose in Chinese patients with type 2 diabetes mellitus uncontrolled on metformin monotherapy: Results of a Phase IV open-label randomized controlled study (the SMART study). Diabetes Obes Metab. (2017) 19:1513–20. doi: 10.1111/dom.12942 28296055

[120] Wu H Liu J Lou Q Liu J Shen L Zhang M . Comparative assessment of the efficacy and safety of acarbose and metformin combined with premixed insulin in patients with type 2 diabetes mellitus. Medicine. (2017):96:e7533. doi: 10.1097/MD.0000000000007533 28858080 PMC5585474

[121] Sanjari M Gholamhoseinian Najar A Asadikaram G Mashayekhi M Ghaseminejad TA. The safety and efficacy of Rosa damascena extract in patients with type II diabetes: preliminary report of a triple blind randomized acarbose controlled clinical trial. J Kerman Univ Med Sci. (2019) 26:22–35. doi: 10.22062/jkmu.2019.87271

[122] Yang HK Lee S-H Shin J Choi Y-H Ahn Y-B Lee B-W . Acarbose add-on therapy in patients with type 2 diabetes mellitus with metformin and sitagliptin failure: a multicenter, randomized, double-blind, placebo-controlled study. Diabetes Metab J. (2019) 43:287–301. doi: 10.4093/dmj.2018.0054, PMID: 30604599 PMC6581543

[123] Gao F Ma X Peng J Lu J Lu W Zhu W . The effect of Acarbose on glycemic variability in patients with type 2 diabetes mellitus using premixed insulin compared to metformin (AIM): an open-label randomized trial. Diabetes Technol Ther. (2020) 22:256–64. doi: 10.1089/dia.2019.0290, PMID: 31638433

[124] Ren G Ma X Jiao P. Effect of liraglutide combined with metformin or acarbose on glucose control in type 2 diabetes mellitus and risk factors of gastrointestinal adverse reactions. Am J Transl Res. (2022) 14:3207. PMID: 35702127 PMC9185051

[125] Gao B Gao W Wan H Xu F Zhou R Zhang X . Efficacy and safety of alogliptin versus acarbose in Chinese type 2 diabetes patients with high cardiovascular risk or coronary heart disease treated with aspirin and inadequately controlled with metformin monotherapy or drug-naive: a multicentre, randomized, open-label, prospective study (ACADEMIC). Diabetes Obes Metab. (2022) 24:991–9. doi: 10.1111/dom.14661 35112779 PMC9314577

[126] Li J Ji J Liu F Wang L. Insulin glargine and Acarbose in the treatment of elderly patients with diabetes. Pakistan J Med Sci. (2019) 35:609. doi: 10.12669/pjms.35.3.86, PMID: 31258562 PMC6572939

[127] Mo D Liu S Ma H Tian H Yu H Zhang X . Effects of acarbose and metformin on the inflammatory state in newly diagnosed type 2 diabetes patients: a one-year randomized clinical study. Drug Des Devel Ther. (2019) 13:2769. doi: 10.2147/DDDT.S208327 PMC6691948 31496653

[128] Derosa G Maffioli P D'Angelo A Fogari E Bianchi L Cicero AF. Retracted: acarbose on insulin resistance after an oral fat load: a double-blind, placebo controlled study. J Diabetes Complications. (2011) 25:258–66. doi: 10.1016/j.jdiacomp.2011.01.003 21367625

[129] Gao H-w Xie C Wang H-N Lin Y-j Hong T-P. Beneficial metabolic effects of nateglinide versus acarbose in patients with newly-diagnosed type 2 diabetes. Acta Pharmacol Sin. (2007) 28:534–9. doi: 10.1111/j.1745-7254.2007.00534.x, PMID: 17376293

[130] Hanefeld M Fischer S Schulze J Spengler M Wargenau M Schollberg K . Therapeutic potentials of acarbose as first-line drug in NIDDM insufficiently treated with diet alone. Diabetes Care. (1991) 14:732–7. doi: 10.2337/diacare.14.8.732, PMID: 1954810

[131] Hoffmann J Spengler M. Efficacy of 24-week Monotnerapy with Acarbose, Glibenclamide, or placebo in NIDDM patients: the Essen study. Diabetes Care. (1994) 17:561–6. doi: 10.2337/diacare.17.6.561, PMID: 8082525

[132] Li H Xu W Liu J Chen A Liao Z Li Y. Effects of nateglinide and acarbose on glycemic excursions in standardized carbohydrate and mixed-meal tests in drug-naïve type 2 diabetic patients. Biomed Rep. (2013) 1:913–7. doi: 10.3892/br.2013.156, PMID: 24649052 PMC3917008

[133] Nakhaee A Sanjari M. Evaluation of effect of acarbose consumption on weight losing in non-diabetic overweight or obese patients in Kerman. J Res Med Sci. (2013) 18:391. PMID: 24174943 PMC3810572

[134] Jayaram S Hariharan R Madhavan R Periyandavar I Samra S. A prospective, parallel group, open-labeled, comparative, multi-centric, active controlled study to evaluate the safety, tolerability and benefits of fixed dose combination of acarbose and metformin versus metformin alone in type 2 diabetes. J Assoc Physicians India. (2010) 58:679–82. 21510461

[135] Zhou J Deng Z Lu J Li H Zhang X Peng Y . Differential therapeutic effects of nateglinide and acarbose on fasting and postprandial lipid profiles: a randomized trial. Diabetes Technol Ther. (2015) 17:229–34. doi: 10.1089/dia.2014.0299, PMID: 25781235

[136] Holman RR Cull CA Turner RC. A randomized double-blind trial of acarbose in type 2 diabetes shows improved glycemic control over 3 years (UK prospective diabetes study 44). Diabetes Care. (1999) 22:960–4. doi: 10.2337/diacare.22.6.960, PMID: 10372249

[137] Inoue I Shinoda Y Nakano T Sassa M Goto S-i Awata T . Acarbose ameliorates atherogenecity of low-density lipoprotein in patients with impaired glucose tolerance. Metabolism. (2006) 55:946–52. doi: 10.1016/j.metabol.2006.03.002, PMID: 16784969

[138] Little RR Rohlfing CL Sacks DB. Status of hemoglobin A1c measurement and goals for improvement: from chaos to order for improving diabetes care. Clin Chem. (2011) 57:205–14. doi: 10.1373/clinchem.2010.148841, PMID: 21148304

[139] Hanefeld M Cagatay M Petrowitsch T Neuser D Petzinna D Rupp M. Acarbose reduces the risk for myocardial infarction in type 2 diabetic patients: meta-analysis of seven long-term studies. Eur Heart J. (2004) 25:10–6. doi: 10.1016/S0195-668X(03)00468-8, PMID: 14683737

[140] Monnier L Mas E Ginet C Michel F Villon L Cristol J-P . Activation of oxidative stress by acute glucose fluctuations compared with sustained chronic hyperglycemia in patients with type 2 diabetes. JAMA. (2006) 295:1681–7. doi: 10.1001/jama.295.14.1681, PMID: 16609090

[141] Robertson RP. Chronic oxidative stress as a central mechanism for glucose toxicity in pancreatic islet beta cells in diabetes. J Biol Chem. (2004) 279:42351–4. doi: 10.1074/jbc.R400019200, PMID: 15258147

[142] Inoguchi T Battan R Handler E Sportsman JR Heath W King GL. Preferential elevation of protein kinase C isoform beta II and diacylglycerol levels in the aorta and heart of diabetic rats: differential reversibility to glycemic control by islet cell transplantation. Proc Natl Acad Sci USA. (1992) 89:11059–63. doi: 10.1073/pnas.89.22.11059, PMID: 1438315 PMC50483

[143] Martin AE Montgomery PA. Acarbose: An α-glucosidase inhibitor. Am J Health Syst Pharm. (1996) 53:2277–90. doi: 10.1093/ajhp/53.19.2277, PMID: 8893066

[144] Zhang Q Xiao X Li M Li W Yu M Zhang H . Acarbose reduces blood glucose by activating mi R-10a-5p and mi R-664 in diabetic rats. PloS One. (2013) 8:e79697. doi: 10.1371/journal.pone.0079697 24260283 PMC3832586

[145] Kristiansen OP Mandrup-Poulsen T. Interleukin-6 and diabetes: the good, the bad, or the indifferent? Diabetes. (2005) 54:S114–24. doi: 10.2337/diabetes.54.suppl_2.S114, PMID: 16306329

[146] Ruan H Lodish HF. Insulin resistance in adipose tissue: direct and indirect effects of tumor necrosis factor-α. Cytokine Growth Factor Rev. (2003) 14:447–55. doi: 10.1016/S1359-6101(03)00052-2, PMID: 12948526

[147] Choi S-E Choi K-M Yoon I-H Shin J-Y Kim J-S Park W-Y . IL-6 protects pancreatic islet beta cells from pro-inflammatory cytokines-induced cell death and functional impairment in vitro and in vivo. Transpl Immunol. (2004) 13:43–53. doi: 10.1016/j.trim.2004.04.001, PMID: 15203128

[148] Canfora EE Jocken JW Blaak EE. Short-chain fatty acids in control of body weight and insulin sensitivity. Nat Rev Endocrinol. (2015) 11:577–91. doi: 10.1038/nrendo.2015.128, PMID: 26260141

[149] Zadeh-Tahmasebi M Duca FA Rasmussen BA Bauer PV Côté CD Filippi BM . Activation of short and long chain fatty acid sensing machinery in the ileum lowers glucose production in vivo. J Biol Chem. (2016) 291:8816–24. doi: 10.1074/jbc.M116.718460, PMID: 26896795 PMC4861449

[150] Chambers ES Viardot A Psichas A Morrison DJ Murphy KG Zac-Varghese SE . Effects of targeted delivery of propionate to the human colon on appetite regulation, body weight maintenance and adiposity in overweight adults. Gut. (2015) 64:1744–54. doi: 10.1136/gutjnl-2014-307913, PMID: 25500202 PMC4680171

[151] Li FF Fu LY Xu XH Su XF Wu JD Ye L . Analysis of the add-on effect of α-glucosidase inhibitor, acarbose in insulin therapy: a pilot study. Biomed Rep. (2016) 5:461–6. doi: 10.3892/br.2016.744, PMID: 27699014 PMC5038828

[152] Båvenholm PN Efendic S. Postprandial hyperglycaemia and vascular damage-the benefits of acarbose. Diab Vasc Dis Res. (2006) 3:72–9. doi: 10.3132/dvdr.2006.017, PMID: 17058626

[153] Esposito K Nappo F Marfella R Giugliano G Giugliano F Ciotola M . Inflammatory cytokine concentrations are acutely increased by hyperglycemia in humans: role of oxidative stress. Circulation. (2002) 106:2067–72. doi: 10.1161/01.CIR.0000034509.14906.AE, PMID: 12379575

[154] Fernandez-Real J Pickup J. Innate immunity, insulin resistance and type 2 diabetes. Diabetologia. (2012) 55:273–8. doi: 10.1007/s00125-011-2387-y, PMID: 22124608

[155] Hotamisligil GS. Inflammation and metabolic disorders. Nature. (2006) 444:860–7. doi: 10.1038/nature05485, PMID: 17167474

[156] Bäckhed F Ding H Wang T Hooper LV Koh GY Nagy A . The gut microbiota as an environmental factor that regulates fat storage. Proc Natl Acad Sci USA. (2004) 101:15718–23. doi: 10.1073/pnas.0407076101, PMID: 15505215 PMC524219

[157] De Vuyst L Leroy F. Cross-feeding between bifidobacteria and butyrate-producing colon bacteria explains bifdobacterial competitiveness, butyrate production, and gas production. Int J Food Microbiol. (2011) 149:73–80. doi: 10.1016/j.ijfoodmicro.2011.03.003, PMID: 21450362

[158] Macia L Tan J Vieira AT Leach K Stanley D Luong S . Metabolite-sensing receptors GPR43 and GPR109A facilitate dietary fibre-induced gut homeostasis through regulation of the inflammasome. Nat Commun. (2015) 6:6734. doi: 10.1038/ncomms7734 25828455

[159] Lührs H Gerke T Müller J Melcher R Schauber J Boxberger F . Butyrate inhibits NF-κB activation in lamina propria macrophages of patients with ulcerative colitis. Scand J Gastroenterol. (2002) 37:458–66. doi: 10.1080/003655202317316105 11989838

[160] Maeda T Towatari M Kosugi H Saito H. Up-regulation of costimulatory/adhesion molecules by histone deacetylase inhibitors in acute myeloid leukemia cells. Blood. (2000) 96:3847–56. doi: 10.1182/blood.V96.12.3847 PMID: 11090069

[161] Nastasi C Candela M Bonefeld CM Geisler C Hansen M Krejsgaard T . The effect of short-chain fatty acids on human monocyte-derived dendritic cells. Sci Rep. (2015) 5:16148. doi: 10.1038/srep16148 26541096 PMC4635422

[162] Slavin J. Fiber and prebiotics: mechanisms and health benefits. Nutrients. (2013) 5:1417–35. doi: 10.3390/nu5041417, PMID: 23609775 PMC3705355

[163] Glauben R Siegmund B. Inhibition of histone deacetylases in inflammatory bowel diseases. Mol Med. (2011) 17:426–33. doi: 10.2119/molmed.2011.00069, PMID: 21365125 PMC3105130

[164] Lin Y-C Chen Y-C Hsiao H-P Kuo C-H Chen B-H Chen Y-T . The effects of acarbose on chemokine and cytokine production in human monocytic THP-1 cells. Hormones. (2019) 18:179–87. doi: 10.1007/s42000-019-00101-z, PMID: 30827017

[165] Shimobayashi M Albert V Woelnerhanssen B Frei IC Weissenberger D Meyer-Gerspach AC . Insulin resistance causes inflammation in adipose tissue. J Clin Invest. (2018) 128:1538–50. doi: 10.1172/JCI96139, PMID: 29528335 PMC5873875

[166] Maury E Brichard S. Adipokine dysregulation, adipose tissue inflammation and metabolic syndrome. Mol Cell Endocrinol. (2010) 314:1–16. doi: 10.1016/j.mce.2009.07.031, PMID: 19682539

[167] Jernås M Palming J Sjöholm K Jennische E Svensson P-A Gabrielsson BG . Separation of human adipocytes by size: hypertrophic fat cells display distinct gene expression. FASEB J. (2006) 20:1540–2. doi: 10.1096/fj.05-5678fje, PMID: 16754744

[168] Brooks GC Blaha MJ Blumenthal RS. Relation of C-reactive protein to abdominal adiposity. Am J Cardiol. (2010) 106:56–61. doi: 10.1016/j.amjcard.2010.02.017, PMID: 20609648

[169] Chambers ES Morrison DJ Frost G. Control of appetite and energy intake by SCFA: what are the potential underlying mechanisms? Proc Nutr Soc. (2015) 74:328–36. doi: 10.1017/S0029665114001657, PMID: 25497601

[170] McNeil NI Cummings J James W. Short chain fatty acid absorption by the human large intestine. Gut. (1978) 19:819–22. doi: 10.1136/gut.19.9.819, PMID: 30683 PMC1412179

[171] Lin W-H Yang C-Y Kuo S Kuo T-H Roan J-N Li C-Y . Hepatic and cardiovascular safety of acarbose among type 2 diabetes patients with end-stage renal disease: a nationwide population-based longitudinal study. Diabetes Res Clin Pract. (2021) 172:108489. doi: 10.1016/j.diabres.2020.108489, PMID: 33035600

[172] Balfour JA McTavish D. Acarbose. An update of its pharmacology and therapeutic use in diabetes mellitus. Drugs. (1993) 46:1025–54. doi: 10.2165/00003495-199346060-00007, PMID: 7510610

[173] Andrade RJ Lucena MI Rodriguez-Mendizabal M. Hepatic injury caused by acarbose. Ann Intern Med. (1996) 124:931. doi: 10.7326/0003-4819-124-10-199605150-00030, PMID: 8610937

[174] Hsiao P-J Wu K-L Chiu S-H Chan J-S Lin Y-F Wu C-Z . Impact of the use of anti-diabetic drugs on survival of diabetic dialysis patients: a 5-year retrospective cohort study in Taiwan. Clin Exp Nephrol. (2017) 21:694–704. doi: 10.1007/s10157-016-1330-4, PMID: 27599981

[175] Carrascosa M Pascual F Aresti S. Acarbose-induced acute severe hepatotoxicity. Lancet (British Edition). (1997) 349:698–9. doi: 10.1016/S0140-6736(05)60134-1, PMID: 9078205

[176] Zhang L Chen Q Li L Kwong JS Jia P Zhao P . Alpha-glucosidase inhibitors and hepatotoxicity in type 2 diabetes: a systematic review and meta-analysis. Sci Rep. (2016) 6:32649. doi: 10.1038/srep32649 27596383 PMC5011653

[177] Wing RR Lang W Wadden TA Safford M Knowler WC Bertoni AG . Benefits of modest weight loss in improving cardiovascular risk factors in overweight and obese individuals with type 2 diabetes. Diabetes Care. (2011) 34:1481–6. doi: 10.2337/dc10-2415, PMID: 21593294 PMC3120182

[178] Expert Panel Members Jensen MD Ryan DH Donato KA Apovian CM Ard JD Comuzzie AG . Executive summary: guidelines (2013) for the management of overweight and obesity in adults: a report of the American College of Cardiology/American Heart Association Task Force on Practice Guidelines and the Obesity Society published by the Obesity Society and American College of Cardiology/American Heart Association Task Force on Practice Guidelines. Based on a systematic review from the The Obesity Expert Panel, 2013. Obesity. (2014) 22:S5–S39. doi: 10.1002/oby.20821 24961825

[179] Gaddey HL Holder KK. Unintentional weight loss in older adults. Am Fam Physician. (2014) 89:718–22. PMID: 24784334

[180] Stevens J Truesdale KP McClain JE Cai J. The definition of weight maintenance. Int J Obes. (2006) 30:391–9. doi: 10.1038/sj.ijo.0803175, PMID: 16302013

[181] Okada K Yanagawa T Warabi E Yamastu K Uwayama J Takeda K . The α-glucosidase inhibitor acarbose prevents obesity and simple steatosis in sequestosome 1/A170/p 62 deficient mice. Hepatol Res. (2009) 39:490–500. doi: 10.1111/j.1872-034X.2008.00478.x, PMID: 19207582

[182] Tolhurst G Heffron H Lam YS Parker HE Habib AM Diakogiannaki E . Short-chain fatty acids stimulate glucagon-like peptide-1 secretion via the G-protein–coupled receptor FFAR2. Diabetes. (2012) 61:364–71. doi: 10.2337/db11-1019, PMID: 22190648 PMC3266401

[183] Xiong Y Miyamoto N Shibata K Valasek MA Motoike T Kedzierski RM . Short-chain fatty acids stimulate leptin production in adipocytes through the G protein-coupled receptor GPR41. Proc Natl Acad Sci. (2004) 101:1045–50. doi: 10.1073/pnas.2637002100, PMID: 14722361 PMC327148

[184] Samuel BS Shaito A Motoike T Rey FE Backhed F Manchester JK . Effects of the gut microbiota on host adiposity are modulated by the short-chain fatty-acid binding G protein-coupled receptor, Gpr 41. Proc Natl Acad Sci. (2008) 105:16767–72. doi: 10.1073/pnas.0808567105, PMID: 18931303 PMC2569967

[185] Brown AJ Goldsworthy SM Barnes AA Eilert MM Tcheang L Daniels D . The orphan G protein-coupled receptors GPR41 and GPR43 are activated by propionate and other short chain carboxylic acids. J Biol Chem. (2003) 278:11312–9. doi: 10.1074/jbc.M211609200, PMID: 12496283

[186] Inoue D Tsujimoto G Kimura I. Regulation of energy homeostasis by GPR41. Front Endocrinol (Lausanne). (2014) 5:81. doi: 10.3389/fendo.2014.00081, PMID: 24904531 PMC4033597

[187] Wang B Zhao J Zhan Q Wang R Liu B Zhou Y . Acarbose for postprandial hypotension with glucose metabolism disorders: a systematic review and Meta-analysis. Front Cardiovasc Med. (2021) 8:663635. doi: 10.3389/fcvm.2021.759563, PMID: 34095252 PMC8172613

[188] Ando T Okada S Niijima Y Hashimoto K Shimizu H Tsuchiya T . Impaired glucose tolerance, but not impaired fasting glucose, is a risk factor for early-stage atherosclerosis. Diabet Med. (2010) 27:1430–5. doi: 10.1111/j.1464-5491.2010.03144.x, PMID: 21059096

[189] Tominaga M Eguchi H Manaka H Igarashi K Kato T Sekikawa A. Impaired glucose tolerance is a risk factor for cardiovascular disease, but not impaired fasting glucose. The Funagata Diabetes Study. Diabetes Care. (1999) 22:920–4. doi: 10.2337/diacare.22.6.920, PMID: 10372242

[190] Hanefeld M Chiasson JL Koehler C Henkel E Schaper F Temelkova-Kurktschiev T. Acarbose slows progression of intima-media thickness of the carotid arteries in subjects with impaired glucose tolerance. Stroke. (2004) 35:1073–8. doi: 10.1161/01.STR.0000125864.01546.f2, PMID: 15073402

[191] Rudofsky G Jr Reismann P Schiekofer S Petrov D von Eynatten M Humpert PM . Reduction of postprandial hyperglycemia in patients with type 2 diabetes reduces NF-kappa B activation in PBMCs. Horm Metab Res. (2004) 36:630–8. doi: 10.1055/s-2004-825904, PMID: 15486815

[192] Gordon JW Shaw JA Kirshenbaum LA. Multiple facets of NF-κB in the heart: to be or not to NF-κB. Circ Res. (2011) 108:1122–32. doi: 10.1161/CIRCRESAHA.110.226928, PMID: 21527742

[193] Araujo Penna I Canella PR Vieira CS Silva de Sá MF dos Reis RM Ferriani RA. Cardiovascular risk factors are reduced with a low dose of acarbose in obese patients with polycystic ovary syndrome. Fertil Steril. (2007) 88:519–22. doi: 10.1016/j.fertnstert.2006.11.073, PMID: 17418836

[194] Bischoff H. The mechanism of alpha-glucosidase inhibition in the management of diabetes. Clin Invest Med. (1995) 18:303–11. PMID: 8549017

[195] Malaguarnera M Giugno I Panebianco M Pistone G. Beneficial effects of acarbose on familiar hypertriglyceridemias. Int J Clin Pharmacol Ther. (1998) 36:441–5. PMID: 9726698

[196] Hou J Chong ZZ Shang YC Maiese K. Fox O3a governs early and late apoptotic endothelial programs during elevated glucose through mitochondrial and caspase signaling. Mol Cell Endocrinol. (2010) 321:194–206. doi: 10.1016/j.mce.2010.02.037, PMID: 20211690 PMC2857725

[197] Zhang C Zhang L Chen S Feng B Lu X Bai Y . The prevention of diabetic cardiomyopathy by non-mitogenic acidic fibroblast growth factor is probably mediated by the suppression of oxidative stress and damage. PLoS One. (2013) 8:e82287. doi: 10.1371/journal.pone.0085170, PMID: 24349248 PMC3857250

[198] Lee Y Hong Y Lee S-R Chang K-T Hong Y. Autophagy contributes to retardation of cardiac growth in diabetic rats. Lab Anim Res. (2012) 28:99–107. doi: 10.5625/lar.2012.28.2.99, PMID: 22787483 PMC3389845

[199] Zhao Z Huang G Wang B Zhong Y. Inhibition of NF-kappa B activation by pyrrolidine dithiocarbamate partially attenuates hippocampal MMP-9 activation and improves cognitive deficits in streptozotocin-induced diabetic rats. Behav Brain Res. (2013) 238:44–7. doi: 10.1016/j.bbr.2012.10.018, PMID: 23089644

[200] Haffner SM Stern MP Hazuda HP Mitchell BD Patterson JK. Cardiovascular risk factors in confirmed prediabetic individuals: does the clock for coronary heart disease start ticking before the onset of clinical diabetes? JAMA. (1990) 263:2893–8. doi: 10.1001/jama.1990.03440210043030, PMID: 2338751

[201] Behrendt D Ganz P. Endothelial function: from vascular biology to clinical applications. Am J Cardiol. (2002) 90:L40–8. doi: 10.1016/S0002-9149(02)02963-6, PMID: 12459427

[202] Den Besten G Bleeker A Gerding A van Eunen K Havinga R van Dijk TH . Short-chain fatty acids protect against high-fat diet–induced obesity via a PPARγ-dependent switch from lipogenesis to fat oxidation. Diabetes. (2015) 64:2398–408. doi: 10.2337/db14-1213 25695945

[203] Fava F Lovegrove J Gitau R Jackson K Tuohy K. The gut microbiota and lipid metabolism: implications for human health and coronary heart disease. Curr Med Chem. (2006) 13:3005–21. doi: 10.2174/092986706778521814, PMID: 17073643

[204] Stepniakowski KT Goodfriend TL Egan BM. Fatty acids enhance vascular α-adrenergic sensitivity. Hypertension. (1995) 25:774–8. doi: 10.1161/01.HYP.25.4.774, PMID: 7721431

[205] Oishi K Zheng B Kuo J. Inhibition of Na, K-ATPase and sodium pump by protein kinase C regulators sphingosine, lysophosphatidylcholine, and oleic acid. J Biol Chem. (1990) 265:70–5. doi: 10.1016/S0021-9258(19)40196-8, PMID: 2152929

[206] Jose PA Raj D. Gut microbiota in hypertension. Curr Opin Nephrol Hypertens. (2015) 24:403. doi: 10.1097/MNH.0000000000000149, PMID: 26125644 PMC4578629

[207] Furness JB Rivera LR Cho H-J Bravo DM Callaghan B. The gut as a sensory organ. Nat Rev Gastroenterol Hepatol. (2013) 10:729–40. doi: 10.1038/nrgastro.2013.180 24061204

[208] Pluznick J. A novel SCFA receptor, the microbiota, and blood pressure regulation. Gut Microbes. (2014) 5:202–7. doi: 10.4161/gmic.27492, PMID: 24429443 PMC4063845

