# Supplementary material for: The effects of acarbose treatment on cardiovascular risk factors in impaired glucose tolerance and diabetic patients: a systematic review and dose–response meta-analysis of randomized clinical trials
Source: Front Nutr. 2023 Aug 1;10:1084084. doi: 10.3389/fnut.2023.1084084 (PMC10433190; doi:10.3389/fnut.2023.1084084)
Supplement: Supplementary file 1 [file Data_Sheet_1.docx]

**Supplementary file 1**

| PubMed | (Acarbose[Title/Abstract]) AND (Intervention[Title/Abstract] OR "Intervention Study"[Title/Abstract] OR "Intervention Studies"[Title/Abstract] OR "controlled trial"[Title/Abstract] OR randomized[Title/Abstract] OR randomized[Title/Abstract] OR random[Title/Abstract] OR randomly[Title/Abstract] OR placebo[Title/Abstract] OR "clinical trial"[Title/Abstract] OR Trial[Title/Abstract] OR "randomized controlled trial"[Title/Abstract] OR "randomized clinical trial"[Title/Abstract] OR RCT[Title/Abstract] OR blinded[Title/Abstract] OR "double blind"[Title/Abstract] OR "double blinded"[Title/Abstract] OR trial[Title/Abstract] OR "clinical trial"[Title/Abstract] OR trials[Title/Abstract] OR "Pragmatic Clinical Trial"[Title/Abstract] OR "Cross-Over Studies"[Title/Abstract] OR "Cross-Over"[Title/Abstract] OR "Cross-Over Study"[Title/Abstract] OR parallel[Title/Abstract] OR "parallel study"[Title/Abstract] OR "parallel trial"[Title/Abstract]) | 1415 |
| --- | --- | --- |
| WOS | **TOPIC**: (Acarbose) *AND* **TOPIC**: (Intervention  OR "Intervention Study"  OR "Intervention Studies"  OR "controlled trial"  OR randomized  OR randomized  OR random  OR randomly  OR placebo  OR "clinical trial"  OR Trial  OR "randomized controlled trial"  OR "randomized clinical trial"  OR RCT  OR blinded  OR "double blind"  OR "double blinded"  OR trial  OR "clinical trial"  OR trials  OR "Pragmatic Clinical Trial"  OR "Cross-Over Studies"  OR "Cross-Over"  OR "Cross-Over Study"  OR parallel  OR "parallel study"  OR "parallel trial") | 939 |
| Scopus | TITLE-ABS-KEY ( acarbose ) AND TITLE-ABS-KEY ( intervention OR "Intervention Study" OR "Intervention Studies" OR "controlled trial" OR randomized OR randomized OR random OR randomly OR placebo OR "clinical trial" OR trial OR "randomized controlled trial" OR "randomized clinical trial" OR rct OR blinded OR "double blind" OR "double blinded" OR trial OR "clinical trial" OR trials OR "Pragmatic Clinical Trial" OR "Cross-Over Studies" OR "Cross-Over" OR "Cross-Over Study" OR parallel OR "parallel study" OR "parallel trial" ) ) | 3126 |
| All |  | 5480 |
| Duplicates |  | 1238 |
| Remained |  | 4242 |
| Animals |  |  |
| review |  |  |
| unrelated |  |  |
| Full-text |  |  |
|  |  |  |
