# Supplementary material for: The effects of acarbose treatment on cardiovascular risk factors in impaired glucose tolerance and diabetic patients: a systematic review and dose–response meta-analysis of randomized clinical trials
Source: Front Nutr. 2023 Aug 1;10:1084084. doi: 10.3389/fnut.2023.1084084 (PMC10433190; doi:10.3389/fnut.2023.1084084)
Supplement: Supplementary file 2 [file Data_Sheet_2.docx]

A)

B)

C)

D)

E)

F)

G)

H)

I)

J)

K)

L)

M)

N)

O)

P)

Q)

R)

S)

T)

U)

Figure S1. Funnel plots for the effect of acarbose consumption on A) TG (mg/dl); B) TC (mg/dl); C) LDL (mg/dl); D) HDL (mg/dl) ; E) FBG (mg/dl); F) Insulin (pmol/l); G) HbA1c (%); H) HOMA-IR; I) SBP (mmHg); J) DBP (mmHg); K) CRP (mg/l); L) ; IL-6 (pg/ml); M) TNF-α (pg/ml); N) Adiponectin (ng/ml); O) Leptin (ng/ml); P)weight (kg); Q) BMI (kg/m^2^); R) WC (cm); S) ALT (U/L); T) AST (U/L) and U) ALP (U/L).

A)

B)

C)

D)

E)

F)

G)

H)

I)

J)

K)

L)

M)

N)

O)

P)

Q)

R)

S)


T)

U)

Figure S2. Non-linear dose-response relations between acarbose consumption and absolute mean differences. Dose-response relations between dose (mg/day) and absolute mean differences in A) TG (mg/dl); B) TC (mg/dl); C) LDL (mg/dl); D) HDL (mg/dl) ; E) FBG (mg/dl); F) Insulin (pmol/l); G) HbA1c (%); H) HOMA-IR; I) SBP (mmHg); J) DBP (mmHg); K) CRP (mg/l); L) ; IL-6 (pg/ml); M) TNF-α (pg/ml); N) Adiponectin (ng/ml); O) Leptin (ng/ml); P)weight (kg); Q) BMI (kg/m^2^); R) WC (cm); S) ALT (U/L); T) AST (U/L) and U) ALP (U/L).

A)

B)

C)

D)

E)

F)

G)

H)

I)

J)

K)

L)

M)

N)

O)

P)

Q)

R)

S)

T)

U)

Figure S3. Non-linear dose-response relations between acarbose consumption and absolute mean differences. Dose-response relations between duration of intervention (week) and absolute mean differences in A) TG (mg/dl); B) TC (mg/dl); C) LDL (mg/dl); D) HDL (mg/dl) ; E) FBG (mg/dl); F) Insulin (pmol/l); G) HbA1c (%); H) HOMA-IR; I) SBP (mmHg); J) DBP (mmHg); K) CRP (mg/l); L) ; IL-6 (pg/ml); M) TNF-α (pg/ml); N) Adiponectin (ng/ml); O) Leptin (ng/ml); P)weight (kg); Q) BMI (kg/m^2^); R) WC (cm); S) ALT (U/L); T) AST (U/L) and U) ALP (U/L).

A)

B)

C)

D)

E)

F)

G)

H)

I)

J)

K)

L)

M)

N)

O)

P)

Q)

R)

S)

T)

U)

Figure S4. linear dose-response relations between acarbose consumption and absolute mean differences. Dose-response relations between dose (mg/day) and absolute mean differences in A) TG (mg/dl); B) TC (mg/dl); C) LDL (mg/dl); D) HDL (mg/dl) ; E) FBG (mg/dl); F) Insulin (pmol/l); G) HbA1c (%); H) HOMA-IR; I) SBP (mmHg); J) DBP (mmHg); K) CRP (mg/l); L) ; IL-6 (pg/ml); M) TNF-α (pg/ml); N) Adiponectin (ng/ml); O) Leptin (ng/ml); P)weight (kg); Q) BMI (kg/m^2^); R) WC (cm); S) ALT (U/L); T) AST (U/L) and U) ALP (U/L).

A)

B)

C)

D)

E)

F)

G)

H)

I)

J)

K)

L)

M)

N)

O)

P)

Q)

R)

S)

T)

U)

Figure S5. linear dose-response relations between acarbose consumption and absolute mean differences. Dose-response relations between duration of intervention (week) and absolute mean differences in A) TG (mg/dl); B) TC (mg/dl); C) LDL (mg/dl); D) HDL (mg/dl) ; E) FBG (mg/dl); F) Insulin (pmol/l); G) HbA1c (%); H) HOMA-IR; I) SBP (mmHg); J) DBP (mmHg); K) CRP (mg/l); L) ; IL-6 (pg/ml); M) TNF-α (pg/ml); N) Adiponectin (ng/ml); O) Leptin (ng/ml); P)weight (kg); Q) BMI (kg/m^2^); R) WC (cm); S) ALT (U/L); T) AST (U/L) and U) ALP (U/L).
